# Lower-temperature bulk depolymerization of commercial polymethacrylates

**DOI:** 10.1038/s41557-026-02232-4

**Published:** 2026-09-02

**Authors:** Richard Whitfield, Victoria Lohmann, Mikhail Agrachev, Hongsik Kim, Hyun Suk Wang, Nethmi De Alwis Watuthanthrige, Nghia P. Truong, Tae-Lim Choi, Gunnar Jeschke, Athina Anastasaki

**Affiliations:** 1 Laboratory of Sustainable Polymers, Department of Materials, ETH Zurich, Zurich, Switzerland; 2 Institute for Molecular Physical Science, Department of Chemistry and Applied Biosciences, ETH Zurich, Zurich, Switzerland; 3 Laboratory of Polymer Chemistry, Department of Materials, ETH Zurich, Zurich, Switzerland

**Keywords:** Polymer synthesis, Polymerization mechanisms

## Abstract

Chemical recycling back to monomer represents an attractive methodology to tackle plastic waste. However, current reports focus on either thermally unstable ‘designer’ polymers or highly diluted solution depolymerizations. Here we report a simple, yet efficient, bulk main-chain scission pathway, whereby the presence of *N*-hydroxyphthalimides triggers a hydrogen-atom-transfer reaction on the polymer backbone, followed by the rapid and near-quantitative depolymerization of polymethacrylates. Notably, this strategy can be directly applied to crude commercial polymers, does not rely on preinstalled end-groups or sacrificial co-monomers, operates at relatively low temperatures in the absence of light irradiation and precludes the use of a solvent, therefore enabling direct isolation of pristine monomers in high yields (∼90%). The methodology is compatible with polymethacrylates synthesized by either anionic, free radical or controlled radical polymerization and can also be applied to ultrahigh-molecular-weight materials (>10^6^ Da).

**Figure:**
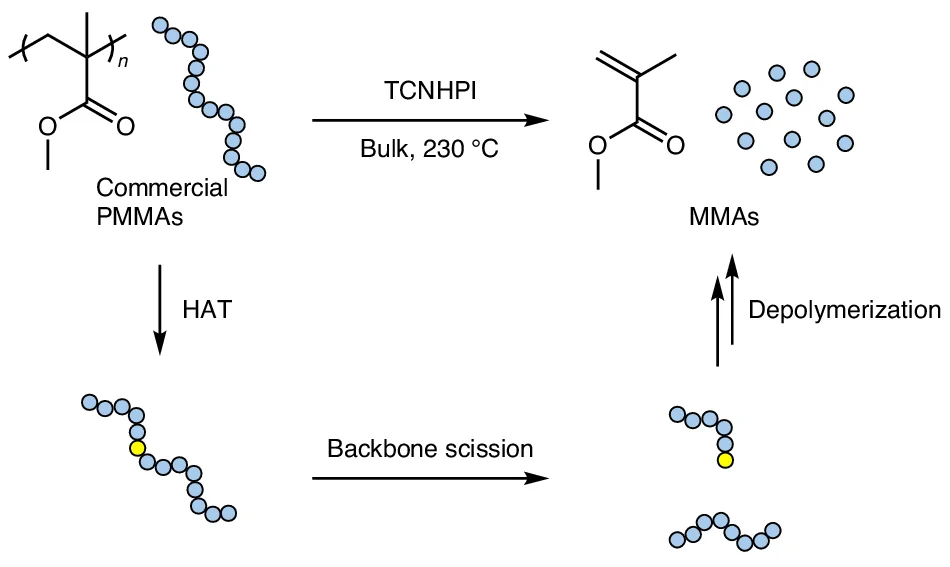

## Abstract (web summary)

Chemical recycling of polymers has largely relied on thermally unstable tailor-made materials or highly diluted depolymerization conditions. Now, a bulk main-chain scission pathway enables the rapid, low-temperature depolymerization of polymethacrylates. This methodology can be applied to commercial polymers without the need for preinstalled end-groups or sacrificial co-monomers, allowing high-yield recovery of monomers.

## Main

Polymers are ubiquitous in everyday life in the twenty-first century; however, despite the incredible benefits of these materials, their excessive use and limited recyclability are causing an environmental crisis with long-lasting repercussions on health and ecology^1,2^. Unfortunately, the most common recycling method, mechanical recycling, essentially involves heating and reprocessing plastics into lower-quality products, often with diminished mechanical properties and performance^3–5^. To overcome product deterioration and preserve natural resources, depolymerization offers an attractive proposition of breaking down and converting polymers back to their constituent monomers, which can then be repolymerized to give pristine materials^6–8^. Although this chemical recycling strategy is already being commercialized for certain polyesters, such as poly(ethylene terephthalate) (PET), it is much more difficult to implement for vinyl polymers containing all-carbon backbones, which lack heteroatoms and are less susceptible to chemical attack and chain breakage^8–11^. To break such stable bonds, pyrolysis is typically employed, but the very high temperatures required (∼450 °C) result in energy waste, side reactions and odorous by-products that contaminate the purity of the recovered monomer^12–17^.

To circumvent these barriers and lower the temperature of depolymerization, reversible deactivation radical polymerization (RDRP), also known as controlled radical polymerization^18,19^, has been utilized as an alternative strategy^8^. By installing labile end-groups, such as halogens or thiocarbonylthio compounds, depolymerization can proceed at much lower temperatures. For example, polymers synthesized by atom-transfer radical polymerization (ATRP) can undergo a rapid depolymerization at 170 °C, resulting in high percentages of recovered monomer^20–23^. In a similar vein, materials prepared by reversible addition fragmentation chain-transfer (RAFT) polymerization can be efficiently depolymerized at even lower temperatures, usually 120 °C, under either strictly thermal conditions or by combining heat and light^24–28^. Notwithstanding these exciting achievements, current solution RDRP depolymerization methodologies rely on high dilutions to effectively operate (that is, to reach high conversions), while the necessity of preinstalled end-groups increases associated costs and limits the applicability of depolymerization to certain designed materials (that is, high-molecular-weight polymers cannot undergo efficient depolymerization)^29–31^. Recently, our group showed that polymethacrylates synthesized by free radical polymerization can be recycled back to monomer when dissolved in a chlorinated solvent and irradiated with visible light^32^. Apart from the use of a highly toxic chlorinated solvent and undesirable photochemical reaction conditions that hinder scalability, the system still required high dilution to yield high percentages of recovered monomer.

**Fig. 1:**
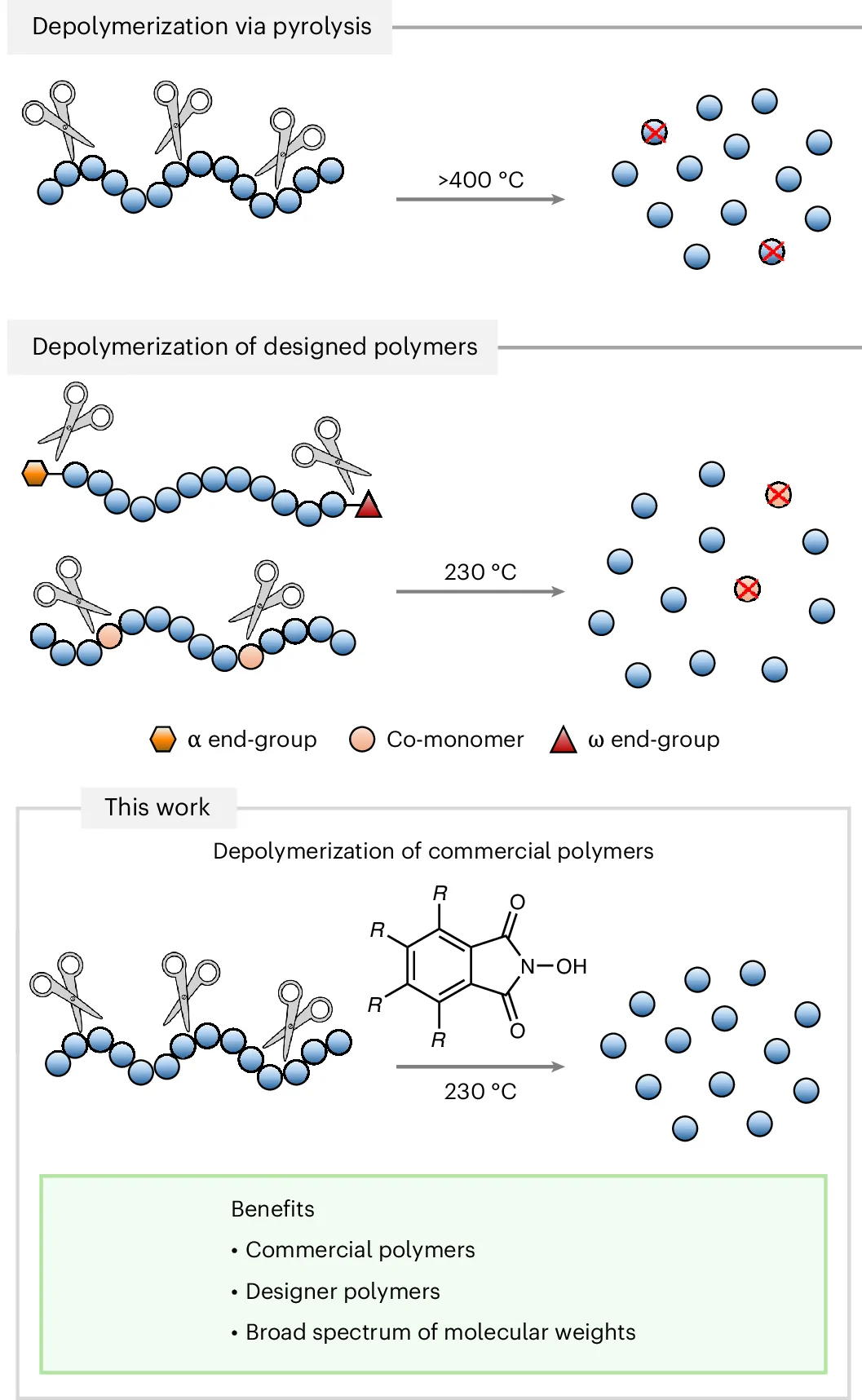
Schematic comparing previous methods and the approach in this study for bulk depolymerization. The current approach allows lower-temperature bulk depolymerization to be achieved for commercial polymers without the need for preinstalled labile groups. The balls containing the red cross symbolize degraded monomers.

To avoid the use of a solvent, bulk depolymerization strategies have also been explored. Once again, RDRP materials demonstrated their potential to dramatically lower the bulk depolymerization temperature when compared to traditional pyrolysis (Fig. 1, top and middle). For instance, Sumerlin and co-workers cleverly installed *N*-hydroxyphthalimide esters and trithiocarbonates at the α- and ω-chain ends, allowing an efficient depolymerization to occur at 230 °C (ref. ^33^). In a completely different approach, Matyjaszewski’s group reported the bulk depolymerization of chlorine-terminated polymers synthesized by ATRP in the presence of a copper catalyst^34^. In a similar fashion, our group leveraged the transformation of ATRP and RAFT end-groups to macromonomers, resulting in a lower-temperature, solvent-free depolymerization^35^. More recently, Summerlin and co-workers used mechanochemically induced chain scission in the presence of a methacrylic co-monomer to end-functionalize polymers, prior to an efficient bulk depolymerization^36^. Nevertheless, all these approaches necessitate the presence of labile end-groups to be able to operate at reduced temperatures. To expand the scope of depolymerizable materials, the so-called ‘co-monomer approach’ has also been investigated (Fig. 1, middle). Diao and co-workers reported the incorporation of a small amount of *α*-methylstyrene during the polymerization of poly(methyl methacrylate) (PMMA), with subsequent depolymerization resulting in 50% monomer recovery at 210 °C (ref. ^37^). On the same day, Summerlin’s group independently presented an alternative strategy whereby phthalimide ester-containing monomers were chosen to be copolymerized with MMA^38^. It is noted that even though both strategies utilize polymers prepared by free radical polymerization, they rely on sacrificial co-monomers, which enable a lower-temperature depolymerization at the expense of a decreased thermal stability of the original materials, therefore limiting the potential application of these copolymers to address the plastic waste challenge.

In this work, we report a solvent-free main-chain-initiated pathway that can be directly applied to both ‘designer’ and commercial polymers, enabling a lower-temperature depolymerization. In the absence of any co-monomers, and on the addition of a *N*-hydroxyimide, a commercially available and inexpensive reagent, a hydrogen-atom transfer (HAT) reaction on the polymer backbone takes place prior to a near-quantitative bulk depolymerization (Fig. 1, bottom).

## Results and discussion

**Fig. 2:**
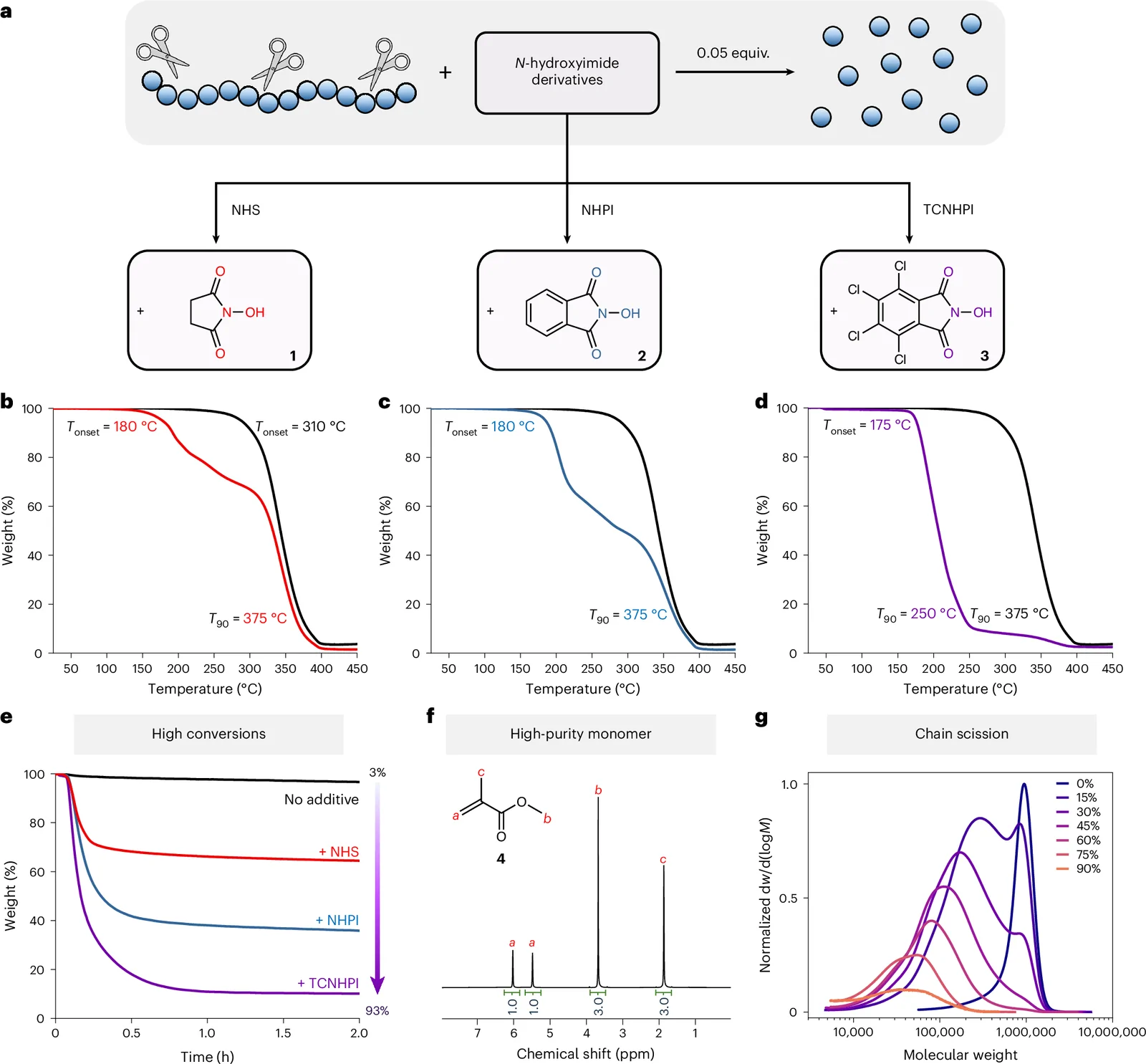
Lower-temperature bulk depolymerization of PMMA facilitated by *N*-hydroxyimide derivatives. **a**, Depolymerization scheme and the structures of various *N*-hydroxyimides. **b**–**d**, Dynamic TGA experiments illustrating the depolymerization profile in the presence of NHS (**1**) (**b**), NHPI (**2**) (**c**) and TCNHPI (**3**) (**d**). Experiments were performed with a heating rate of 1 °C min^−1^ from 25 °C to 450 °C. **e**, Analogous isothermal TGA experiments for the various *N*-hydroxyimide derivatives. Experiments were performed with 0.05 equiv. of additive with respect to the repeat unit at 230 °C for 2 h. **f**, ^1^H NMR spectrum of the monomer obtained from a distillation experiment, referenced to deuterated chloroform 7.26 ppm. Experiments were performed with 0.05 equiv. of **3** with respect to the repeat unit at 230 °C. **g**, SEC traces at various extents of depolymerization. Source data

The use of *N*-hydroxy species, such as *N*-hydroxyphthalimide (NHPI, **2**), in conjunction with metal catalysts, radical initiators or external stimuli, has been widely used as a versatile tool for the functionalization and degradation of organic compounds^39–41^. Inspired by previous work with both small molecule modifications and polymer degradation^42–47^, we sought to develop a depolymerization methodology that would not rely on preinstalled end-groups or co-monomers. In particular, we initially envisioned utilizing NHPI in the presence of a radical stimulus to trigger a hydrogen-atom abstraction from the polymer backbone, as a means of facilitating lower-temperature bulk depolymerization of unfunctionalized polymers (Fig. 2a). To start our investigation, PMMA (**5**) prepared by anionic polymerization (*M*_n_ ≈ 700 kDa) was selected as the model polymer due to its inert hydrogen end-groups and high molecular weight (Supplementary Fig. 1). By simply heating the polymer alone in a thermogravimetric analysis (TGA) pan from 25 °C to 450 °C (heating rate, 1 °C min^−1^), a mass-loss onset (*T*_onset_) temperature of 310 °C was recorded and a temperature of 380 °C was required for 90% polymer mass loss (*T*_90_, Fig. 2b, black, and Supplementary Fig. 2). This control experiment highlights the high temperatures required for the bulk depolymerization of unfunctionalized polymers. In a second control experiment in which NHPI and PMMA were gradually heated together, we serendipitously discovered a much earlier depolymerization onset at 180 °C, followed by 60% of mass loss at 310 °C (Fig. 2c, blue). Although 375 °C was still a prerequisite to achieve 90% mass loss, the earlier mass-loss onset reveals that, in stark contrast to previous literature reports that necessitated an external radical trigger or stimulus, NHPI alone can initiate a lower-temperature depolymerization pathway^40^ (Supplementary Fig. 3). In fact, the addition of the initiator di-*tert*-butyl peroxide as a radical initiator slightly reduced the extent of the low-temperature mass loss, demonstrating a clear benefit of using just a single additive (Supplementary Figs. 4 and 5). It should be highlighted that in our initial experiments, 0.05 equiv. of NHPI with respect to the repeat unit concentration was used; however, when the concentration was increased eight-fold, no meaningful improvement was recorded (Supplementary Table 1).

These results prompted us to explore commercially available alternatives with the aim of further increasing the low-temperature mass loss. In particular, alternative structures containing N–O–H bonds were selected, namely, *N*-hydroxysuccinimide (NHS, **1**) and *N*-hydroxytetrachlorophthalimide (TCNHPI, **3**), because these have been reported to act as effective HAT agents^48,49^. When depolymerization was performed with NHS, as in the case of NHPI, an earlier onset of 180 °C was evident, albeit only 33% of mass loss was recorded by 310 °C, suggesting that this compound was less effective than NHPI in achieving HAT (Fig. 2b, red, and Supplementary Fig. 3). However, when TCNHPI was utilized, a slightly lower depolymerization onset of 175 °C followed by a large drop of the *T*_90_ to 250 °C was observed, thus demonstrating an efficient lower-temperature reaction (Fig. 2d, green, and Supplementary Fig. 3).

To validate these initial findings, and to potentially further lower the reaction temperature, isothermal measurements were performed in the presence of each of the three compounds with the temperature kept constant at 230 °C for 2 h. As expected, TCNHPI generated the highest mass loss at 93%, followed by NHPI at 63% and NHS at 35% (Fig. 2e, green, blue and red, respectively, Supplementary Table 2 and Supplementary Fig. 6). We attribute this better performance of the TCNHPI to the lower volatility of this compound at our reaction temperatures, resulting in a longer time in which the HAT reaction can occur (Supplementary Fig. 7). This was supported by an additional experiment in which after the reaction with NHPI had plateaued, the addition of a second batch of NHPI resulted in further mass loss (83% in total; Supplementary Fig. 8). To further strengthen this point, we also performed experiments with a dimeric NHPI derivative *N*,*N*′-dihydroxypyromellitimide, which enabled monomer recoveries exceeding 90% (Supplementary Fig. 9). It should be highlighted that these experiments demonstrate very high monomer recoveries while using a non-chlorinated HAT agent, which is a clear benefit over previous literature results^32^. Altogether, these results contrast the data obtained in the absence of any additive, where negligible, if any, mass loss occurred, further illustrating the importance of the addition of the *N*-hydroxyimide species (Fig. 2e, black).

Our next aim was to ascertain the extent and purity of the monomer obtained from the process. A distillation experiment was performed and ^1^H NMR spectroscopy of the distillate revealed that the observed mass loss was due to the exclusive regeneration of MMA monomer in high purity (**4**, Fig. 2f). It should be highlighted that the monomer obtained was colourless and this high purity was only possible due to the lower depolymerization temperatures used, which highlights once more the importance of the *N*-hydroxyimide for this process^13^. Following this, reactions were performed at even lower temperatures, with 91%, 88% and 86% mass loss achieved at 220 °C, 210 °C and 200 °C, respectively (Supplementary Table 3 and Supplementary Fig. 10). Interestingly, 70% of mass loss occurred at as low a temperature as 180 °C, which represents a >250 °C reduction compared with traditional pyrolysis methodologies^12–15^. We also highlight the benefit of our approach being purely thermal, rather than requiring an additional stimulus, as this makes it much simpler to implement, more easily scalable and creates the possibility of future compatibility with existing pyrolysis infrastructure.

To further investigate the potential advantages of the low-temperature nature of this depolymerization, experiments were conducted with Al(OH)_3_-filled PMMA mixtures. This is a material that is produced on a large scale, mainly in kitchen surfaces, and has previously been shown to be incompatible with pyrolysis due to thermal degradation of the Al(OH)_3_. This is because at high temperatures, Al(OH)_3_ releases water that can subsequently react with MMA and PMMA, resulting in large amounts of undesirable side products^50^. To investigate this, stability studies were performed to investigate the compatibility of the Al(OH)_3_ with our lower reaction temperatures. While degradation and mass loss was observed at 450 °C, pleasingly, at 180 °C, the Al(OH)_3_ remained fully stable with negligible mass (Supplementary Fig. 11). We therefore selected 180 °C as the reaction temperature and conducted experiments with PMMA and a range of concentrations of Al(OH)_3_ (10–50 wt%). In all cases, high extents of polymer scission and around 70% depolymerization were successfully achieved (Supplementary Figs. 12 and 13). These results were comparable to the reaction performed in the absence of the Al(OH)_3_, confirming that our process is compatible with filled PMMA. This offers a clear contrast to conventional pyrolysis, where successful chemical recycling of this material has yet to be realized^50^. In addition, there are also further benefits over previously reported low-temperature approaches, whereby the presence of preinstalled end-groups or the incorporation of co-monomers could both compromise the thermal stability of the resulting polymers^37,38,51^. In contrast, this methodology offers a promising and direct route toward the depolymerization of highly stable polymers. Importantly, the amount of TCNHPI could be successfully decreased from 0.05 equiv. to 0.004 equiv. without compromising the final yield (Supplementary Table 4 and Supplementary Fig. 14). This last result is of particular importance because using smaller amounts of the additive reduces the cost and increases the potential for utility of the process.

We wanted next to investigate the pathway of the reaction and confirm our hypothesis that the depolymerization reaction was proceeding via a HAT scission pathway. Detailed kinetic experiments were therefore performed by sampling the reaction after every 15% of depolymerization and conducting size exclusion chromatography (SEC) (Fig. 2g). As the reaction proceeded, a gradual reduction in the molecular weight was observed over time with a drop from 700 kDa of the starting polymer to 20 kDa after depolymerization was complete. This is consistent with a midchain scission mechanistic pathway: it is expected that the HAT step will generate a backbone radical that can subsequently trigger β scission and produce two lower-molecular-weight fragments—only one of which contains a radical that can trigger depropagation, while the other one is stable and is hence visible in the molecular weight distribution (Supplementary Table 5 and Supplementary Scheme 1). As the reaction progresses and more scission events occur, the molecular weight of the remaining fragments will gradually decrease, which is what is observed in the experimental data. To further confirm this, MatLab simulations were performed to evaluate the evolution of the molecular weight distribution in midchain- and end-group-initiated depolymerization processes (Supplementary Fig. 15). For midchain scission, a steep reduction in molecular weight was observed as the number of random backbone scission events increased, which mirrored our experimental data. We would like to highlight that these results are in contrast to previously reported end-group-triggered approaches in which activation of the end-group results in all the monomer from a given chain being directly obtained^28^. This results in a gradual reduction in the intensity of the molecular weight distribution rather than a shift in molecular weight. Midchain scission approaches therefore offer the potential benefit of chemical recycling of polymers independent of their end-groups, and this will be explored later in the paper.

**Fig. 3:**
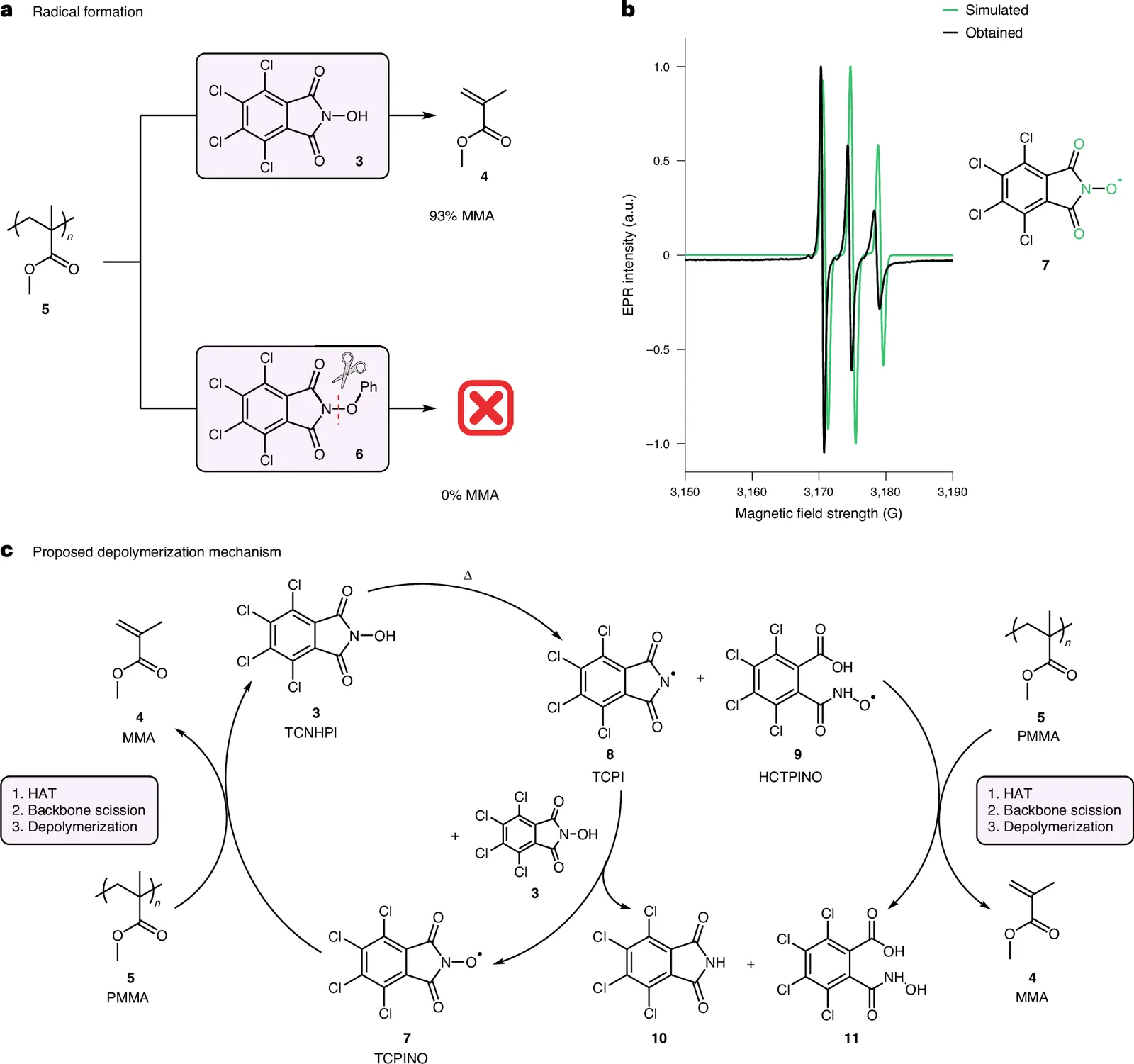
The mechanism of the low-temperature, midchain-induced bulk depolymerization of PMMA (5) to MMA (4) facilitated by *N*-hydroxyimide derivatives. **a**, Depolymerization data comparing TCNHPI (**3**) and TCNPhPI (**6**). **b**, EPR spectrum comparing the obtained data from **3** (black) to those simulated for the TCPINO (**7**) radical. **c**, Scheme for the proposed depolymerization pathway. Source data

To further elucidate the reaction mechanism, an additional series of experiments was performed. Although, based on literature precedence^40^, it is reasonable to assume that the N–OH group is responsible for the radical formation required for the HAT reaction, we initially proposed two likely scenarios for where the specific radical is initially formed: one possibility is a potential breakage of the N–O bond forming both nitrogen and hydroxy radicals; or, alternatively, a breakage of the O–H bond resulting in a nitroxide radical (tetrachlorophthalimide-*N*-oxyl, TCPINO, **7**). To investigate this, we used an additional compound, namely *N*-phenoxytetrachlorophthalimide (TCNPhPI; Supplementary Scheme 2 and Supplementary Figs. 16–18). The presence of the phenyl substitution should, in principle, favour the breakage of the N–O bond. However, when PMMA was mixed with TCNPhPI and a depolymerization experiment performed under the optimized reaction conditions, no mass loss could be observed, suggesting that the homolytic cleavage of the N–O bond was not triggering the HAT pathway (Fig. 3a and Supplementary Fig. 19). Next, electron paramagnetic resonance (EPR) measurements were conducted. As shown in Fig. 3b, the obtained spectrum demonstrates a much better agreement between the experimental and the simulated signals (experimental, *a*_iso_(^14^N) = 4.0 G; simulation (nitroxide), *a*_iso_(^14^N) = 4.1 G), based on density functional theory (DFT)-calculated parameters for the nitroxide radical, as opposed to the nitrogen radical (nitrogen, *a*_iso_(^14^N) = 11.0 G), thus indicating the formation of the TCPINO species at 230 °C. However, these data raise the question of how nitroxide radicals form at high temperatures. It seemed unlikely that homolytic cleavage of an O–H bond would occur under our reaction conditions, but to firmly exclude this possibility, we performed DFT calculations to evaluate the strength of the various bonds. Considering that the bond dissociation energies (BDEs) of the various O–H bonds were calculated to be in the range of 70–100 kcal mol^−1^, it was concluded that breaking these very strong bonds at temperatures between 180 and 230 °C was not realistically feasible and that our initial hypotheses were not correct (Supplementary Tables 6–8). An alternative possibility was that TCNHPI could partially degrade, resulting in radical formation. This scenario was inspired by a recent publication, wherein the decomposition of NHPI revealed the formation of radicals^52^. Indeed, upon heating TCNHPI alone, EPR measurements demonstrated that radical formation was only possible at high temperatures (>190 °C; Supplementary Fig. 20) and notable mass loss was observed by TGA, suggesting a range of volatile products had formed and evaporated (Supplementary Figs. 21 and 22). Additional degradation products were subsequently analysed by gas chromatography–mass spectrometry (GC–MS) and the identification of tetrachlorophthalimide (**10**) and tetrachlorophthalic anhydride (Supplementary Figs. 23–27), indicated the potential formation and HAT reaction of an imidyl radical (TCPI, **8**) and a ring-opened nitroxide radical (HTCPINO, **9**; Supplementary Scheme 3, purple). In particular, GC–MS shows the formation of notable amounts of tetrachlorophthalimide, potentially indicating a greater role of the imidyl radical (Supplementary Fig. 28). However, this conflicted with the EPR data, which showed that the TCPINO had dominated (Supplementary Fig. 29). We attributed this difference to the very fast formation and subsequent reaction of the TCPI and HCTPINO species, and hence the lifetimes of these radicals were too short to be measured by EPR. Instead, the TCPI and HCTPINO species could rapidly abstract a hydrogen atom either from a polymer or from another TCNHPI, generating the observed and more stable TCPINO radicals. It should be noted that it was not possible to use spin traps for these more reactive radical species, for example, 5,5-dimethyl-1-pyrroline *N*-oxide, *N*-*tert*-butyl-α-phenylnitrone or 2,4,6-tri-*tert*-butylnitrosobenzene, because these all decomposed before the depolymerization temperature had been reached (Supplementary Fig. 25). To investigate further, DFT calculations were performed to verify the relative reactivities of the various radical forms (TCPINO, HTCPINO and TCPI) and these demonstrated that the most effective HAT agent is the TCPI (N–H BDE = 123.0 kcal mol^−1^), while lower BDEs were observed for both the open and closed nitroxides (O–H BDE = 83.6 kcal mol^−1^ for TCPINO versus 80.2 kcal mol^−1^ for HTCPINO; Supplementary Fig. 30 and Supplementary Table 9). Altogether, this indicates that there may be a greater contribution from the TCPI radical, but it cannot be excluded that HTCPINO and TCPINO play a role at these elevated temperatures.

**Fig. 4:**
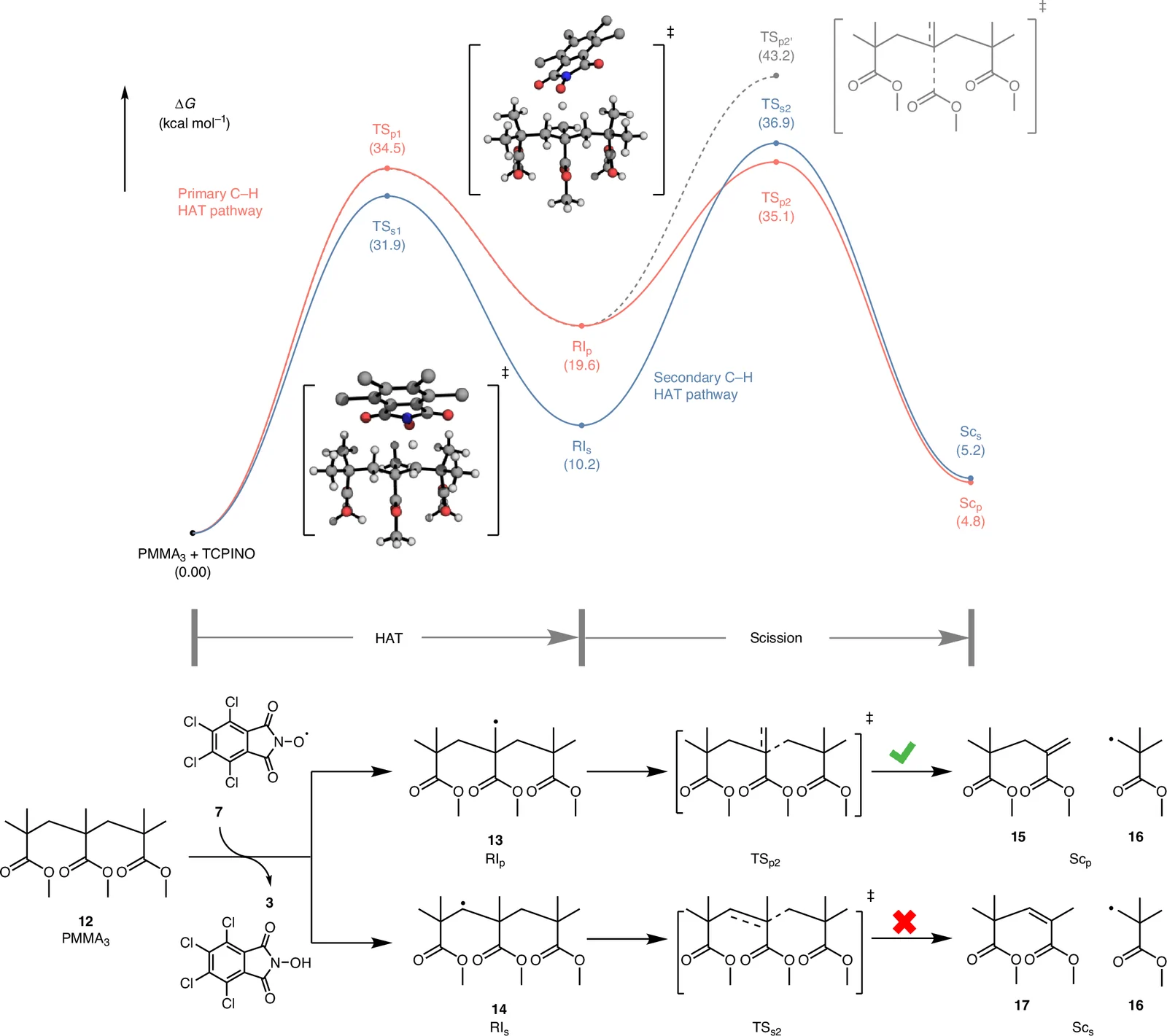
DFT calculations to show the feasibility of the TCPINO (7) radical to do primary or secondary HAT reactions on the PMMA backbone. Calculations were performed at the M06-2X/6-311++G(d,p)//M06-2X/6-31G(d,p) level at a temperature of 503.15 K. Blue atoms represent nitrogen, and red atoms represent oxygen. Δ*G*, Gibbs free energy; RI, radical intermediate; TS, transition state; p, primary; s, secondary; Sc, scission. Source data

We next sought to establish the potential site of the HAT reaction on the polymer; it can either take place on the side chain via the methyl ester or on the polymer backbone. To exclude the role of HAT activation on the side reaction, we examined the bulk depolymerization of poly(phenyl methacrylate), a polymer whose side chain consists of substantially stronger C(*sp*^2^)–H bonds that should strongly disfavour HAT (Supplementary Fig. 31). High percentages of monomer generation alongside a high extent of scission were observed by TGA and SEC (Supplementary Figs. 32 and 33), thereby strongly suggesting that HAT takes place on the polymer backbone triggering depolymerization (Fig. 4). Additional DFT calculations were then conducted to further assess the feasibility of the HAT reaction on the backbone. More specifically, TCPINO was selected as the HAT agent and calculations indicated that the primary radical pathway is favoured, with the transition-state free-energy barrier for chain scission only marginally lower than that for the initial HAT (35.1 kcal mol^−1^ versus 34.5 kcal mol^−1^). This demonstrated that once formed, the primary radical readily triggers chain scission, while, in contrast, the secondary radical pathway shows the opposite trend (36.9 kcal mol^−1^ versus 31.9 kcal mol^−1^), suggesting that the secondary radical intermediate preferentially undergoes reverse HAT rather than chain scission (Fig. 4, Supplementary Fig. 34 and Supplementary Table 10). Last, but not least, EPR studies were also evaluated to confirm that the radicals formed upon heating TCNHPI were indeed responsible for the HAT reaction. TCNHPI (0.05 equiv.) was mixed with PMMA and heated to 230 °C, and EPR showed negligible, if any, signal, thus indicating the rapid consumption of TCPINO in the presence of polymer (Supplementary Fig. 35a). To further validate this finding, fourfold TCNHPI (0.2 equiv.) was subsequently used alongside PMMA, resulting in an initial short-lived signal of the TCPINO radical, which had fully vanished after 5 min (Supplementary Fig. 35b). Taken altogether, these results highlight the active role of TCNHPI-derived radicals in achieving HAT on the polymer backbone.

**Fig. 5:**
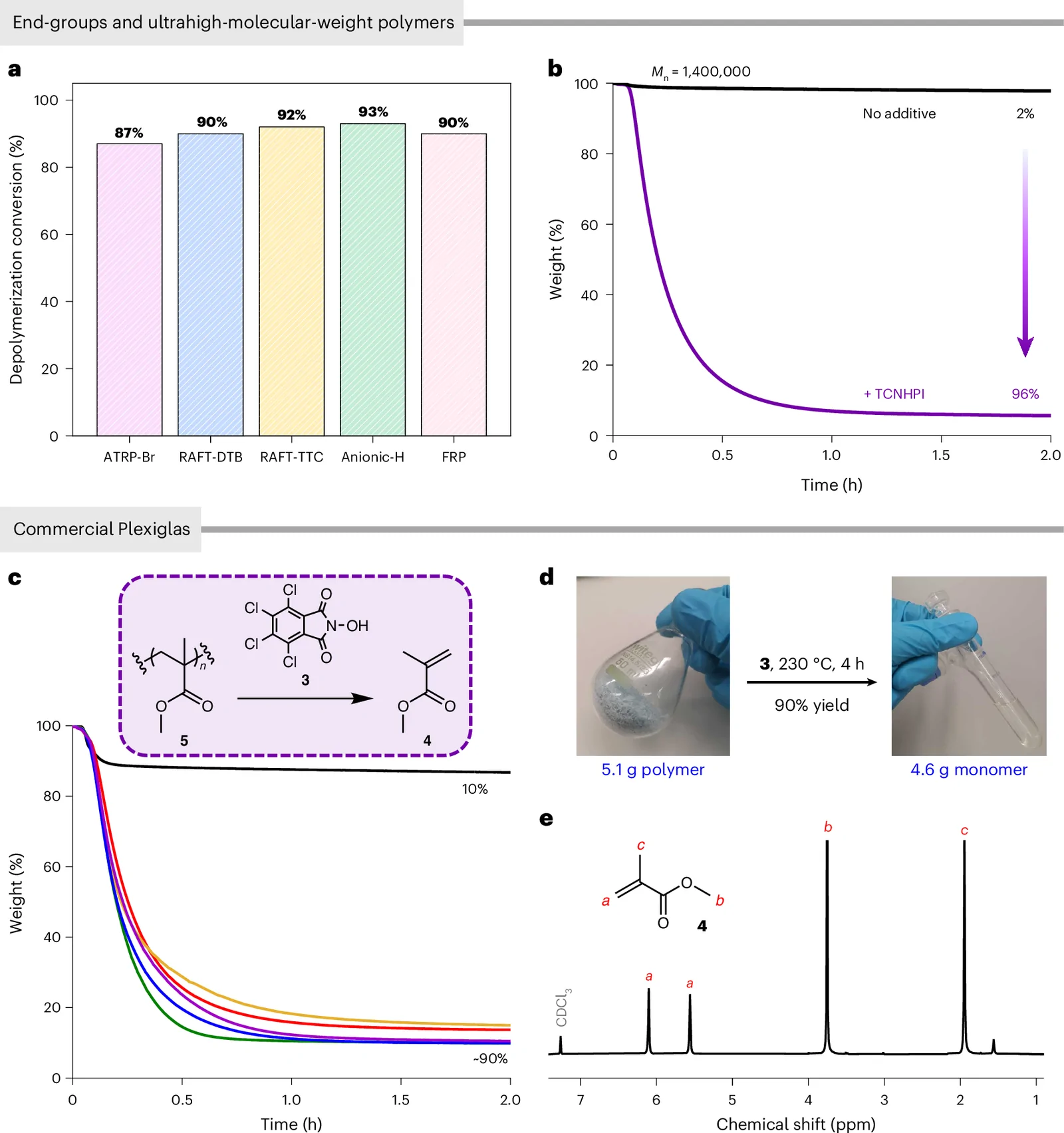
The scope of lower-temperature bulk depolymerization triggered by TCNHPI (3). **a**, Extent of depolymerization for polymers with various end-groups (bromine, dithiobenzoate (DTB), trithiocarbonate (TTC), non-functional) synthesized by ATRP, RAFT polymerization, anionic polymerization and free radical polymerization (FRP). **b**, Depolymerization of ultrahigh-molecular-weight polymer (*M*_n_ = 1,400 kDa). **c**, Depolymerization of various colours of commercial Plexiglas in the presence and absence of **3**. The colours of the lines represent the colours of the Plexiglas used, except for purple which was a mixed Plexiglas reaction. All reactions were performed with 0.05 equiv. of **3** with respect to repeat unit at 230 °C for 2 h. **d**, Scheme and photographs for the blue commercial Plexiglas starting material and the monomer obtained from the distillation. **e**, ^1^H NMR spectrum of the monomer obtained from the distillation. Source data

As indicated by the proposed mechanism, the developed midchain depolymerization pathway should, in principle, be tolerant of different end-groups. A polymer synthesized by anionic polymerization (that is, C–H end-group) was used as the model material, resulting in up to 93% depolymerization. A series of polymers with various end-groups were then subjected to our judiciously optimized conditions, including a Br-terminated polymer synthesized by ATRP, two RAFT polymers bearing either a dithiobenzoate or a trithiocarbonate chain-end, and a free radical polymer containing a mixture of end-groups arising from the different termination pathways (*M*_n_ = 90–180 kDa; Fig. 5a, Supplementary Figs. 36–39 and Supplementary Table 11). Notably, in all cases, very high percentages of regenerated monomer were obtained (up to 92%), thus highlighting the versatility of the approach (Supplementary Table 11 and Supplementary Figs. 40–43). It should be noted that the 87% of depolymerization for the ATRP polymer was slightly lower than that of the other polymers, which was attributed to the lower molecular weight of the starting material. However, by adjusting the reaction conditions, it was possible to increase the depolymerization further (94%; Supplementary Figs. 44 and 45). Further experiments were then conducted to probe the potential of this technique in affording the bulk depolymerization of a broader range of molecular weight polymers. First, lower-molecular-weight materials were investigated. Polymers of 100 kDa, 33 kDa and 25 kDa were synthesized and, on depolymerization, substantial monomer regeneration was observed, namely, 91%, 82% and 66%, respectively (Supplementary Figs. 46–48). This was, however, lower than that achieved with higher-molecular-weight materials, a finding attributed to the greater ratio of polymer chains to HAT agent and a reduced extent of monomer generation after each HAT activation event due to the shorter average length of the resulting polymer fragments. To confirm this, additional experiments were performed with the 25-kDa polymer with a higher concentration of HAT agent; the depolymerization proceeded to 87%, demonstrating that even lower-molecular-weight PMMAs can undergo a highly efficient reaction (Supplementary Fig. 49). In addition, experiments were conducted with an ultrahigh-molecular-weight PMMA (*M*_n_ = 1,400 kDa; Supplementary Fig. 50). Pleasingly, in the presence of TCNHPI, as much as 96% depolymerization conversion was obtained (Fig. 5b). The kinetics of the reaction and subsequent SEC analysis demonstrated a midchain scission depolymerization pathway with a similar profile to that of the lower-molecular-weight materials. It is noted that the remaining 4% of unreacted material corresponded to low-molecular-weight PMMA (22 kDa; Supplementary Table 12 and Supplementary Figs. 51–53), which could be further depolymerized on addition of a second batch of TCNHPI, allowing a near-quantitative total yield to be achieved (>99%; Supplementary Fig. 54). Altogether these results demonstrate that this depolymerization is compatible with a wide molecular weight range (25–1,400 kDa), fully covering and extending beyond the molecular weight range of commercially produced materials. It should be highlighted that if the polymer structure is changed from a polymethacrylate to a polyacrylate, polymer scission can also be achieved, but monomer was not regenerated (Supplementary Figs. 55 and 56). This is attributed to the lower radical stability of the backbone radical formed and is currently the subject of further investigation.

Lastly, we were keen to explore the applicability of the system directly to commercial products. More specifically, Plexiglas materials of various colours (Supplementary Fig. 57) were successfully depolymerized up to 90% conversion despite containing co-monomers and other impurities such as plasticizers, dies, radical inhibitors, etc. (Fig. 5c, Supplementary Fig. 58 and Supplementary Table 13). The blue Plexiglas material was then selected to undergo a larger-scale distillation depolymerization (5-g scale), resulting in a 90% yield and the generation of high-purity monomer, as evidenced by ^1^H NMR (Fig. 5d and Supplementary Table 14). Taken altogether, these results showcase the robustness of the methodology to successfully operate with a wide range of commercial materials. Although the process has so far been demonstrated at laboratory scale, several features give it credible industrial potential: it operates at much lower temperatures than traditional pyrolysis, produces higher-purity colourless monomer and may be compatible with existing pyrolysis infrastructure. In addition, it requires only small amounts of a single inexpensive and industrially relevant additive^53^, and can be applied to previously non-recycled materials polymer composites, such as filled PMMA^50^. Nevertheless, future studies will be necessary to assess the feasibility of scale-up and further development would be required to advance towards practical implementation.

## Conclusion

We have presented a simple-to-perform, one-step main-chain bulk depolymerization methodology which can be applied to both ‘designer’ and commercial polymeric materials. Key to the approach is the use of *N*-hydroxyimides, which can trigger a HAT reaction followed by a rapid depolymerization at temperatures substantially lower than traditional pyrolysis (180–230 °C). Irrespective of the polymer end-group and the initial molecular weight, up to 99% of regenerated monomer can be obtained in a facile manner. Importantly, this strategy does not require sacrificial co-monomers or preinstalled end-groups and enables the direct acquisition of pristine monomer in high yields. This methodology creates many new opportunities for the lower-temperature chemical recycling of polymeric materials.

## Methods

### Midchain-induced depolymerization: dynamic TGA experiment

A method was created with the desired conditions, that is, a 1 °C min^−1^ heating rate from 25 °C to 450 °C, and a background measurement was performed with an empty platinum crucible. These background data were then subtracted from all the obtained measurements. To perform the depolymerization reactions typically a stock mixture of 43.6 mg of ground PMMA was mixed with either 2.44 mg of NHS, 3.44 mg of NHPI or 6.40 mg of TCNHPI (0.05 equiv. with respect to repeat unit) and ∼6 mg of this mixture was added to a platinum crucible. The crucible was then inserted into a Mettler Toledo TGA/DSC 3+ STARe instrument via the autosampler and measurement was performed with the aforementioned method and a 100 ml min^−1^ flow rate of nitrogen.

### Midchain-induced depolymerization: isothermal TGA experiment

A method was created with the desired conditions, that is, a 50 °C min^−1^ heating rate from 25 °C to 230 °C followed by a 2-h hold at 230 °C, and a background measurement was performed with an empty platinum crucible. These background data were then subtracted from all the obtained measurements. To perform the depolymerization reactions typically a stock mixture of 43.6 mg of ground polymer was mixed with either 2.44 mg of NHS, 3.44 mg of NHPI or 6.40 mg of TCNHPI (0.05 equiv. with respect to repeat unit) and ∼6 mg of this mixture was added to a platinum crucible. The crucible was then inserted into a Mettler Toledo TGA/DSC 3+ STARe instrument via the autosampler and measurement was performed with the aforementioned method and a 100 ml min^−1^ flow rate of nitrogen. The sample was then removed from the TGA, for possible ^1^H NMR or SEC analysis.

The amount of the *N*-hydroxyimide derivative, the temperature and the polymer were adjusted depending on the desired reaction conditions.

### Midchain-induced depolymerization: kinetics

Six isothermal depolymerization reactions were performed. Typically, a stock mixture of 43.6 mg of ground polymer was mixed with 6.40 mg of TCNHPI (0.05 equiv. with respect to repeat unit) and this mixture was separated into six platinum crucibles. These were inserted one after the other into a Mettler Toledo TGA/DSC 3+ STARe instrument via the autosampler and heated to 230 °C under a 100 ml min^−1^ flow rate of nitrogen. The reaction was stopped and the sample removed from the TGA when the desired mass loss had been reached (that is, 15%, 30%, 45%, 60%, 75%, 90%). The remaining sample was then measured by SEC.

### Midchain-induced depolymerization: distillation experiment

A vacuum distillation apparatus was set up with a sand bath on a stirrer hot plate and a single collection flask. Then, 5.1 g of commercial polymer was ground, mixed with 0.74 g of TCNHPI (0.05 equiv. with respect to repeat unit) and placed in a round-bottomed flask. The flask was placed in the sand bath under vacuum and gradually heated up to 230 °C; the temperature was measured using a thermocouple at the boundary between the sand and the glass flask. Once the desired temperature had been reached, the distillation was allowed to proceed for 4 h and the monomer collected was characterized by ^1^H NMR.

## Online content

Any methods, additional references, Nature Portfolio reporting summaries, source data, extended data, supplementary information, acknowledgements, peer review information; details of author contributions and competing interests; and statements of data and code availability are available at https://doi.org/10.1038/s41557-026-02232-4.

## Supplementary information

Supplementary Information Supplementary Figs. 1–58, Tables 1–14, Schemes 1–3 and References.

## Source data

Source Data Fig. 2 Source data for Fig. 2.

Source Data Fig. 3 Source data for Fig. 3.

Source Data Fig. 4 Source data for Fig. 4.

Source Data Fig. 5 Source data for Fig. 5.

## Data Availability

All methods, additional references, Nature Research reporting summaries, source data, extended data, supplementary information, acknowledgements, peer review information; details of author contributions and competing interests; and statements of data have been provided by the authors and are freely available. Source data are provided with this paper.
