# Supplementary material for: Lower-temperature bulk depolymerization of commercial polymethacrylates
Source: Nat Chem. 2026 Sep 2;18(11):1979–87. doi: 10.1038/s41557-026-02232-4 (PMC13719524; doi:10.1038/s41557-026-02232-4)
Supplement: Supplementary file 1 [file 41557_2026_2232_MOESM1_ESM.pdf]

nature chemistry

Supplementary information https://doi.org/10.1038/s41557-026-02232-4

Lower-temperature bulk depolymerization of commercial polymethacrylates

In the format provided by the authors and unedited

Contents

Experimental **2**

Materials and Instrumentation **2**

^{1}H Nuclear magnetic resonance 2

Size exclusion chromatography 2

Thermal Gravimetric Analysis 2

Mass Spectrometry 2

Electron Paramagnetic Resonance 2

Density Functional Theory 3

MATLAB Simulations 3

General Procedures **4**

Depolymerization 4

Mid-Chain Induced Depolymerization: Dynamic TGA Experiment 4

Mid-Chain Induced Depolymerization: Isothermal TGA Experiment 4

Mid-Chain Induced Depolymerization: Kinetics 4

Mid-Chain Induced Depolymerization: Distillation Experiment 4

Polymer Synthesis 5

Free Radical Polymerization of PhMA 5

Atom Transfer Radical Polymerization of MMA 5

Reversible-Addition Fragmentation Chain Transfer Polymerization of MMA 5

Small Molecule Synthesis 6

Synthesis of *N*-phenoxytetrachlorophthalimide (4,5,6,7-tetrachloro-2-phenoxyisoindoline-1,3-dione, TCNPhPI) 6

Additional Schemes and Figures **7**

Development of Mid-Chain Bulk Depolymerization 7

Mechanism of Mid-Chain Bulk Depolymerization 17

Expanding the Scope of Mid-Chain Bulk Depolymerization 40

References **54**

Experimental

Materials and Instrumentation

All materials were purchased from Sigma Aldrich or Fischer Scientific and used as received unless otherwise stated. All monomers were filtered through a column of basic alumina before usage. PMMA prepared by anionic and free radical polymerization were purchased from Supelco and Sigma Aldrich, respectively, and were used as received.

^{1}H Nuclear magnetic resonance

Spectra were recorded on a Bruker DPX-300 spectrometer in deuterated chloroform ($CDCl_3$). Chemical shifts are given in ppm downfield from tetramethylsilane referenced to residual $CHCl_3$ protons.

Size exclusion chromatography

SEC analysis of polymer samples was performed using a Shimadzu modular system comprising of a CBM-20A system controller, an SIL-20A automatic injector, a 10.0 μm beadsize guard column (50 × 7.5 mm) followed by three KF-805L columns (300 × 8 mm, bead size: 10 μm, pore size maximum: 5000 Å), an SPD-20A ultraviolet detector, and an RID-20A differential refractive-index detector. The temperature of the columns was maintained at 40 °C using a CTO-20A oven. The eluent was *N,N*-dimethylacetamide (HPLC grade, with 0.03% w/v LiBr) and the flow rate was kept at 1 mL/minute using an LC-20AD pump. A molecular weight calibration curve was produced using commercial narrow molecular weight distribution poly(methyl methacrylate) standards with molecular weights ranging from 5000 to 1.5×10^6 Da (Agilent Technologies EasiVials).

Thermal Gravimetric Analysis

TGA measurements were performed on a Mettler Toledo TGA/DSC 3+ stare system equipped with a Mettler Toledo ultra-micro balance. Dynamic measurements were performed with a 1 °C/minute heating rate and isothermal measurements were performed with a 50 °C/minute heating rate until the desired temperature was reached. This temperature was then maintained for 2 or 5 hours. All experiments were performed under an inert atmosphere with a 100 ml/minute nitrogen flow rate. We should highlight that not all weight loss is necessarily attributed to depolymerization. There can also be minor contributions from the loss of polymer end-groups or small amounts of residual solvent present from the synthesis or purification. However, efforts were made to minimise these, by preparing polymers with a high degree of polymerization and drying all polymers under vacuum prior to the measurement of depolymerization.

Mass Spectrometry

Measurements were performed on a Thermo Scientific QExactive GC Orbitrap (GC-S2) with Ion-Trap (OrbiTrap) and electron impact (EI) as ion source. These measurements were performed by the Molecular and Biomolecular Analysis Service (MoBiAS) at ETH Zürich. The N-hydroxyimide derivative was preheated in a TGA for 20 minutes at 230 °C prior to GC-MS measurement.

Electron Paramagnetic Resonance

EPR spectroscopy experiments were performed using a custom-built setup (microwave frequency = 9.2 GHz, center field = 3172 G, sweep width = 150 G, modulation amplitude = 0.5 G, modulation frequency = 100 kHz, microwave power = 1.983 mW, power attenuation = 20 dB, conversion time = 12.05 ms, time constant = 10.24 ms). A quartz capillary (inner diameter = 0.8 mm) was placed inside an EPR quartz tube (Wilmad; inner diameter = 2.8 mm), which was loaded with the sample. The EPR tube was housed at the center of a homemade water-cooled high-temperature resonator,[1] which was installed into a continuous wave EPR spectrometer (Bruker EMX) operating at X-band frequencies. Helium gas was directed through the central capillary during the measurement. *In situ* experiments were carried out while continuously recording EPR spectra using a 2D acquisition mode enabling a time-resolved monitoring of the process. All of the spectra were simulated using the EasySpin package running in Matlab.[2] The simulations of the hyperfine interactions of different molecular structures were performed applying Kohn-Sham density functional theory, using a B3LYP functional with a spin-unrestricted shell and a 6-31G+(d,p) basis set and a continuum solvent model in the Gaussian software.[3]

Density Functional Theory

DFT calculations were performed using the Gaussian 09 suite.[3] Geometry optimizations, modredundant scans, and vibrational frequency calculations were carried out with the B3LYP[4,5] functional and the 6-31G(d,p)[6] basis set. After geometry optimization, single-point energies were re-evaluated using the triple-ζ 6-311++G(d,p) basis set.[6] For the bond dissociation energy (BDE) calculations, the following enthalpy of formation values were used (Tables S6-S7).[7] The depolymerization simulations presented in Figure 3e were performed at a temperature of 503.15 K (230 °C). Unless otherwise noted, all other calculations were conducted at a temperature of 298.15 K.

MATLAB Simulations

To simulate the evolution of size exclusion chromatography (SEC) chromatograms during polymer degradation, a custom Monte Carlo algorithm was developed in MATLAB R2024b. The initial mass distribution (d w/ d log M) was rigorously derived from the experimental detector signal using the exact numerical Jacobian of the calibration curve to prevent interpolation artifacts. From this continuous distribution, a statistically representative ensemble of 108 discrete polymer chains was generated via inverse transform sampling. Two degradation mechanisms were modeled. In the unzipping model, chains were completely removed from the detectable ensemble with a uniform probability corresponding to the conversion parameter c, preserving the shape of the distribution while scaling the total mass. For the random scission model, the number of scission events per chain followed a Poisson distribution with a mean scaling linearly with initial chain mass (λ = n · M0/Mn0, where n is the scission severity parameter). To account for localized volatilization or the generation of undetectable oligomers, each scission event explicitly induced a targeted mass loss (e.g., 25%) in the cleaved fragment. Following the stochastic degradation process, the surviving polymer fragments were rebinned onto a high-resolution, logarithmically spaced grid (3,000 bins) and mathematically transformed back into height-equivalent SEC detector signals using the original calibration Jacobian. This ensured strict mass conservation within the model limits and enabled direct, mathematically perfectly aligned comparisons with the raw experimental chromatograms.

General Procedures

Depolymerization

Mid-Chain Induced Depolymerization: Dynamic TGA Experiment

A method was created with the desired conditions, i.e. a 1 °C/minute heating rate from 25 °C to 450 °C and a background measurement was performed with an empty platinum crucible. This background data was then subtracted from all the obtained measurements. Subsequently, depolymerization reactions were performed. Typically, a stock mixture of 43.6 mg of ground PMMA (poly(methyl methacrylate) was mixed with either 2.44 mg of NHS, 3.44 mg of NHPI or 6.40 mg of TCNHPI (0.05 equivalents w.r.t. repeat unit) and approximately 6 mg added to a platinum crucible. This crucible was then inserted into the TGA/DSC 3+ star instrument via the autosampler and measurement was performed with the aforementioned method and a 100 mL/minute flow rate of nitrogen.

Mid-Chain Induced Depolymerization: Isothermal TGA Experiment

A method was created with the desired conditions, i.e. a 50 °C/minute heating rate from 25 °C to 230 °C followed by a 2 hour hold at 230 °C and a background measurement was performed with an empty platinum crucible. This background data was then subtracted from all the obtained measurements. Subsequently, depolymerization reactions were performed. Typically, a stock mixture of 43.6 mg of ground polymer were mixed with either 2.44 mg of NHS, 3.44 mg of NHPI or 6.40 mg of TCNHPI (0.05 equivalents w.r.t. repeat unit) and approximately 6 mg added to a platinum crucible. This crucible was then inserted into the TGA/DSC 3+ star instrument via the autosampler and measurement was performed with the aforementioned method and a 100 mL/minute flow rate of nitrogen. The sample was then removed from the TGA, for possible ^{1}H NMR or SEC analysis.

The amount of the N-hydroxyimide derivative, the temperature and the polymer were adjusted depending on the desired reaction conditions.

Mid-Chain Induced Depolymerization: Kinetics

Six isothermal depolymerization reactions were performed. Typically, a stock mixture of 43.6 mg of ground polymer was mixed with 6.40 mg of TCNHPI (0.05 equivalents w.r.t. repeat unit) and this mixture was separated into 6 platinum crucibles. One after another these were inserted into the TGA/DSC 3+ star instrument via the autosampler and heated to 230 °C under a 100 mL/minute flow rate of nitrogen. Reaction was stopped and the sample removed from the TGA when the desired mass loss had been reached (i.e. 15%, 30%, 45%, 60%, 75%, 90%). The remaining sample was then measured by SEC.

Mid-Chain Induced Depolymerization: Distillation Experiment

A vacuum distillation was set-up with a sand bath on a stirrer hot plate and a single collection flask. 5.1 g of commercial polymer was ground, mixed with 0.74 g of TCNHPI (0.05 equivalents w.r.t. repeat unit)) and placed in a round-bottomed flask. The flask was placed in the sand bath under vacuum and was gradually heated up to 230 °C. This was measured using a thermocouple at the boundary between the sand and the glass flask. Once the desired temperature had been reached, the distillation was allowed to proceed for 4 hours and the monomer collected was characterized by ^{1}H NMR.

Polymer Synthesis

Free Radical Polymerization of PhMA

5.54 mg of AIBN was placed in a glass vial and a stock solution was formed on its dissolution in 5 mL of MeCN. 500 µL of this stock solution (1 equiv.) was collected and added to a 15 mL glass vial. In addition, a further 4.5 mL of MeCN, a stirrer bar and 2.5 mL of PhMA (4800 equiv.) were added to the vial. It was sealed with a septum prior to bubbling with nitrogen for 15 minutes. The reaction was then placed in an oil bath at 70 °C on a magnetic stirrer hot plate and reaction was performed for 18 hours with a 100 rpm stirring rate. After the reaction was complete, the vial was removed from the heat and opened to air. It was sampled for ^{1}H NMR and SEC, with SEC samples passed through a syringe filter (0.45 µM PTFE membrane) prior to SEC analysis. Purification was performed with 3 precipitations in methanol and the polymer was dried under vacuum for 48 hours.

Atom Transfer Radical Polymerization of MMA

0.252 mg of $CuCl_2$ (0.2 equiv.) and 5.8 µL of N,N,N',N",N"-pentamethyldiethylenetriamine (PMDETA) (3 equiv.) were weighed into a 25 mL round-bottom flask. They were subsequently dissolved in 3 mL of anisole and the flask was sealed with a rubber septum. In a glass vial, 1.61 µL of α-chlorophenylacetate (1 equiv.) and 4 mL methyl methacrylate (MMA, 4000 equiv.) were added, and this was similarly sealed with a rubber septum. In another glass vial, 11.4 mg of tin(II) 2-ethylhexanoate ($Sn(EH)_2$, 3 equiv.) was dissolved in 1 mL anisole and this was also sealed with a rubber septum. All three vessels were deoxygenated by nitrogen bubbling for 15 minutes. After deoxygenation, the monomer/initiator solution was transferred to the round bottom flask (containing the catalyst) and the flask was heated to 70°C in an oil bath. Subsequently, the $Sn(EH)_2$ solution was transferred into the round bottom flask and the reaction was allowed to proceed for 4 hours. It was then stopped by removing the flask from the oil bath and diluting the reaction mixture with acetone in open air. The solution was quickly passed through basic alumina and precipitated in cold methanol twice and subsequently dried under vacuum for 48 hours.

Reversible-Addition Fragmentation Chain Transfer Polymerization of MMA

2.21 mg of 2-cyanoprop-2-yl dithiobenzoate (1 equiv.) was weighed into a 25 mL round bottom flask. 1 mL of acetonitrile was added and the 2-cyanoprop-2-yl dithiobenzoate was subsequently dissolved. In a separate glass vial, a stock solution of AIBN (20.0 mg) was prepared in 2 mL acetonitrile, and 3.28 µL of this solution (0.328 mg, 0.2 equiv.) was transferred to the flask. Next, 4.26 mL of MMA (4.00 g, 4000 equiv.) and a stirrer bar were added, and the flask was sealed with a septum, prior to deoxygenation by nitrogen bubbling for 15 minutes. Polymerization was conducted in an oil bath at 70 °C for 3 days with a 400-rpm stirring rate. Samples were taken periodically under a nitrogen blanket for ^{1}H NMR analysis and passed through a syringe filter (0.45 µM PTFE membrane) prior to SEC analysis. The polymerization was stopped by removing the reaction from the oil bath and removing the septum. The polymer was purified by precipitating in methanol at least twice and dried under reduced pressure at least overnight. For the synthesis of the trithiocarbonate the same procedure was used albeit with 2-cyano-2-propyl dodecyl trithiocarbonate as the CTA.

Small Molecule Synthesis

Synthesis of *N*-phenoxytetrachlorophthalimide (4,5,6,7-tetrachloro-2-phenoxyisoindoline-1,3-dione, TCNPhPI)

The synthesis of TCNHPhPI was carried out using a modified method based on a published procedure for the synthesis of *N*-aryloxyimides.[8] A solution of t-BuOK (0.91 mmol, 1.1 equiv., 103 mg) and 4,5,6,7-tetrachloro-2-hydroxyisoindoline-1,3-dione (0.83 mmol, 1 equiv., 250 mg) in dry DMF (3 mL) was stirred for 10 minutes at room temperature. Afterwards, diphenyliodonium chloride (0.91 mmol, 1.1 equiv., 290 mg) was added in one portion. The reaction was then stirred for 2 hours in an oil bath preheated to 60 °C. The mixture was then cooled to room temperature, extracted with ethyl acetate, washed successively with water and brine, dried over anhydrous Na_2SO_4, and concentrated under reduced pressure. The resulting residue was purified by flash column chromatography on silica gel, using a hexane:ethyl acetate (6:1) mixture as the eluent and collected as a yellowish-white solid. ^{1}H NMR (300 MHz, Acetone) δ 7.27 (ddd, *J* = 9.7, 5.9, 2.4 Hz, 2H), 7.21 – 7.11 (m, 2H), 7.11 – 7.01 (m, 1H). ^{13}C NMR (126 MHz, Acetone) δ 158.85, 158.68, 139.55, 129.84, 129.41, 126.06, 124.49, 113.70.

Additional Schemes and Figures

Development of Mid-Chain Bulk Depolymerization

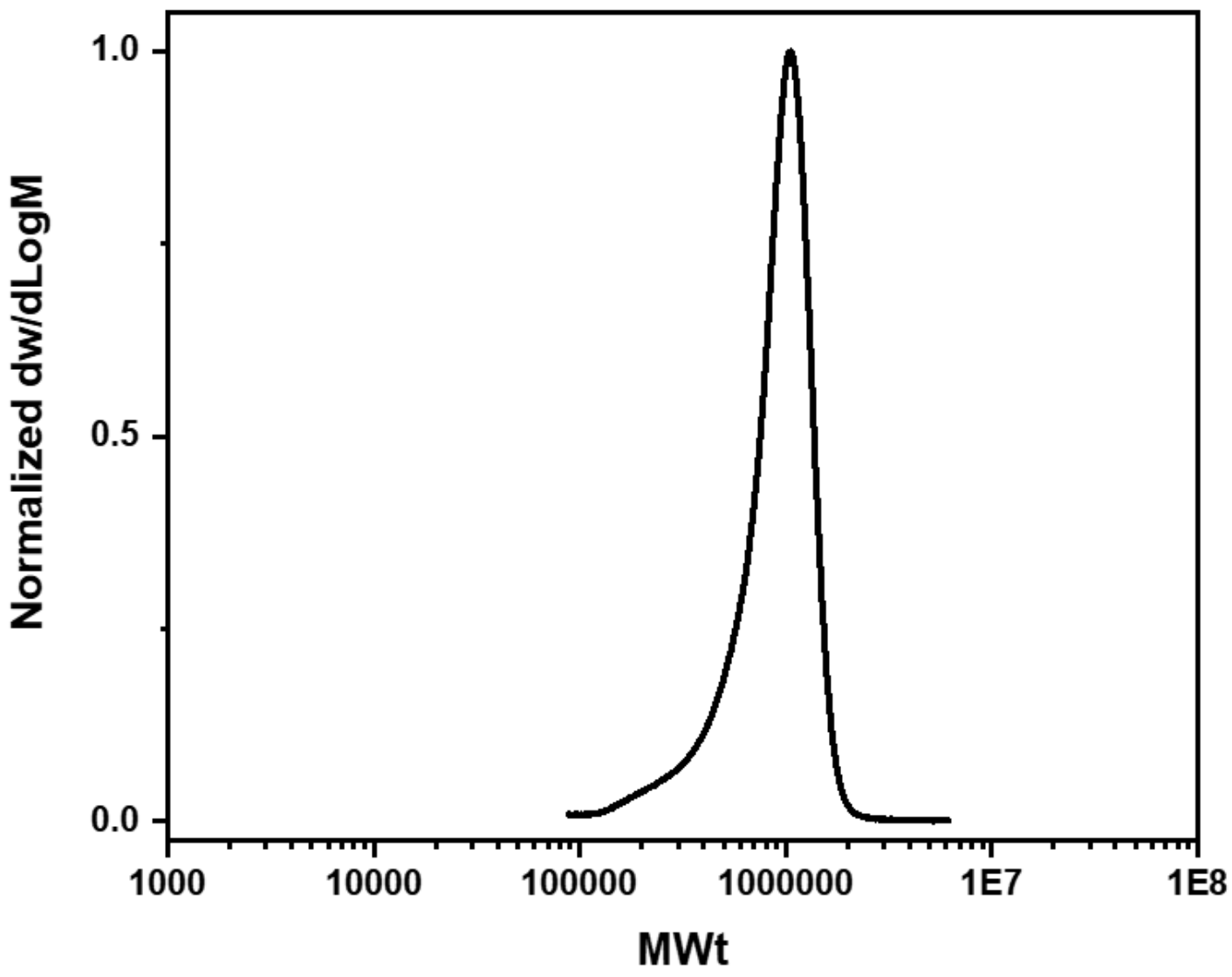

Supplementary Figure 1: SEC trace of PMMA prepared by anionic polymerization. The M_n of this polymer was 715 kDa.

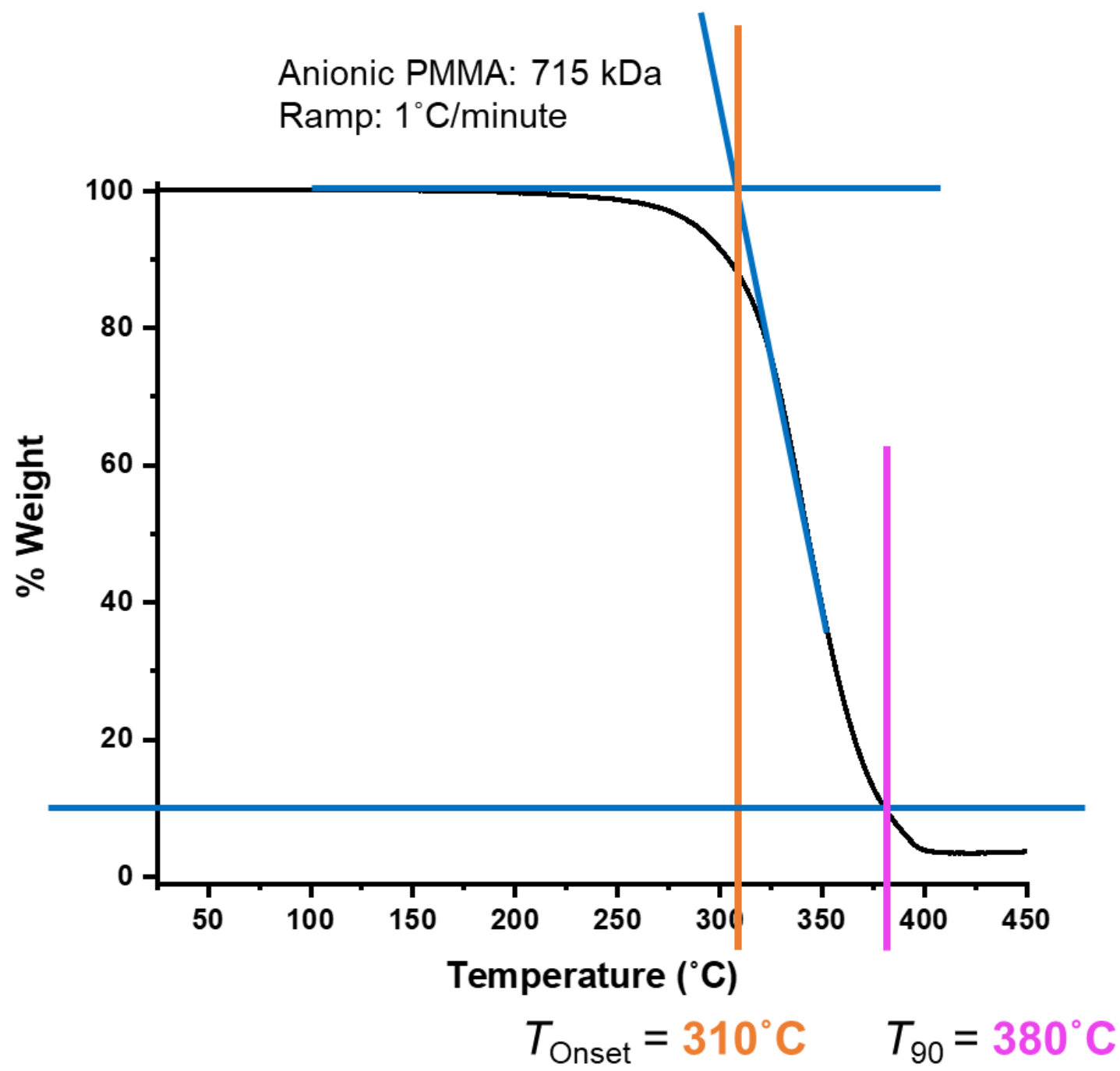

Supplementary Figure 2: TGA trace showing a depolymerization of PMMA prepared by anionic polymerization (black). This is an example demonstrating how the onset (T_{Onset}, orange) and 90% mass loss (T_{90}, pink) points are calculated. The blue lines illustrate the values used for the calculations. Dynamic experiments were performed with a 1 °C/minute heating rate from 25 °C to 450 °C.

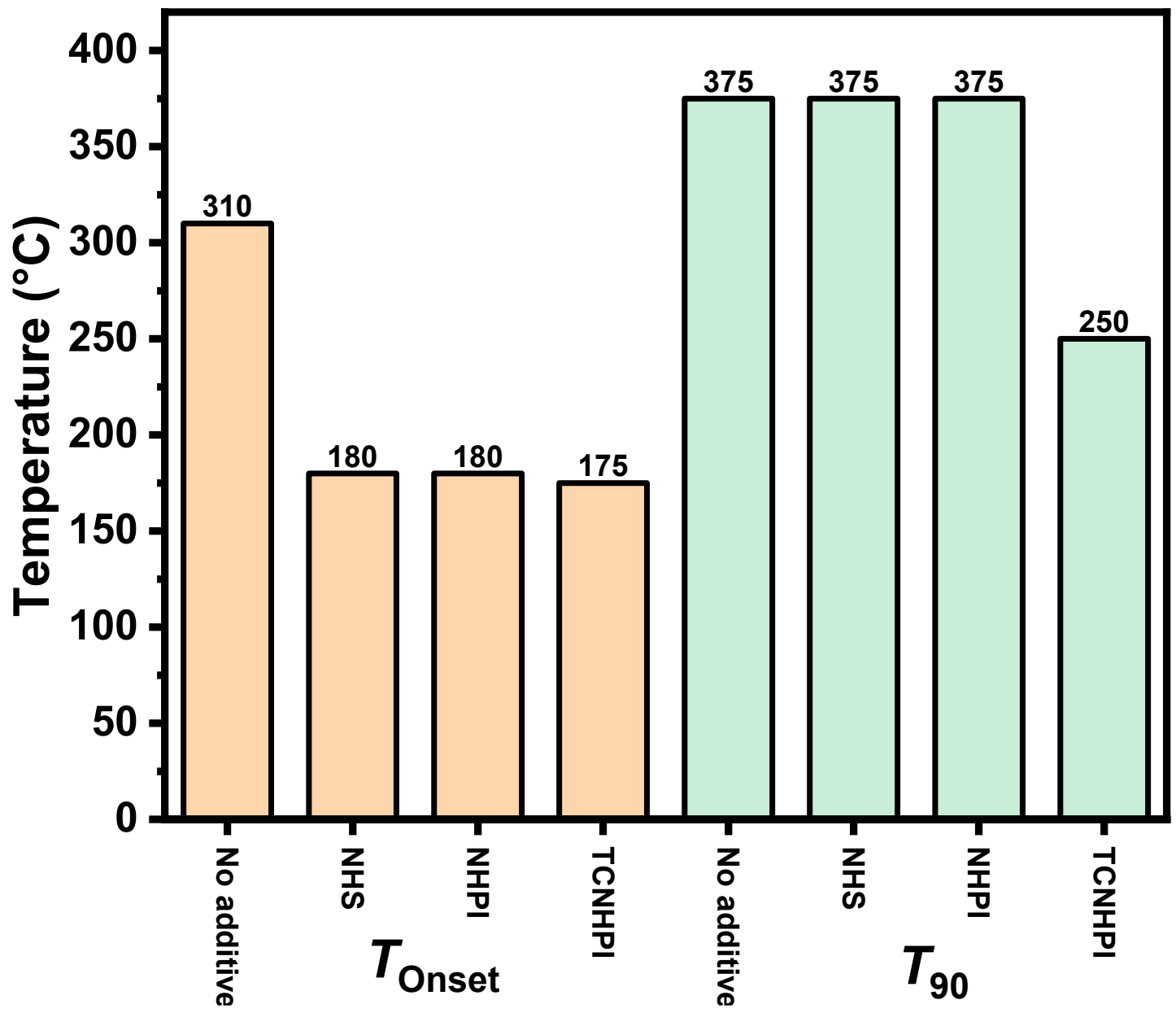

Supplementary Figure 3: Bar graph showing the onset temperature (orange) and the 90% depolymerization temperature (green) for TGA experiments performed with either no additive or 0.05 equivalents of the various n-hydroxyimide derivatives (w.r.t repeat unit concentration). PMMA prepared by anionic polymerization was used for these experiments and dynamic measurements were performed with a 1 °C/minute heating rate from 25 °C to 450 °C.

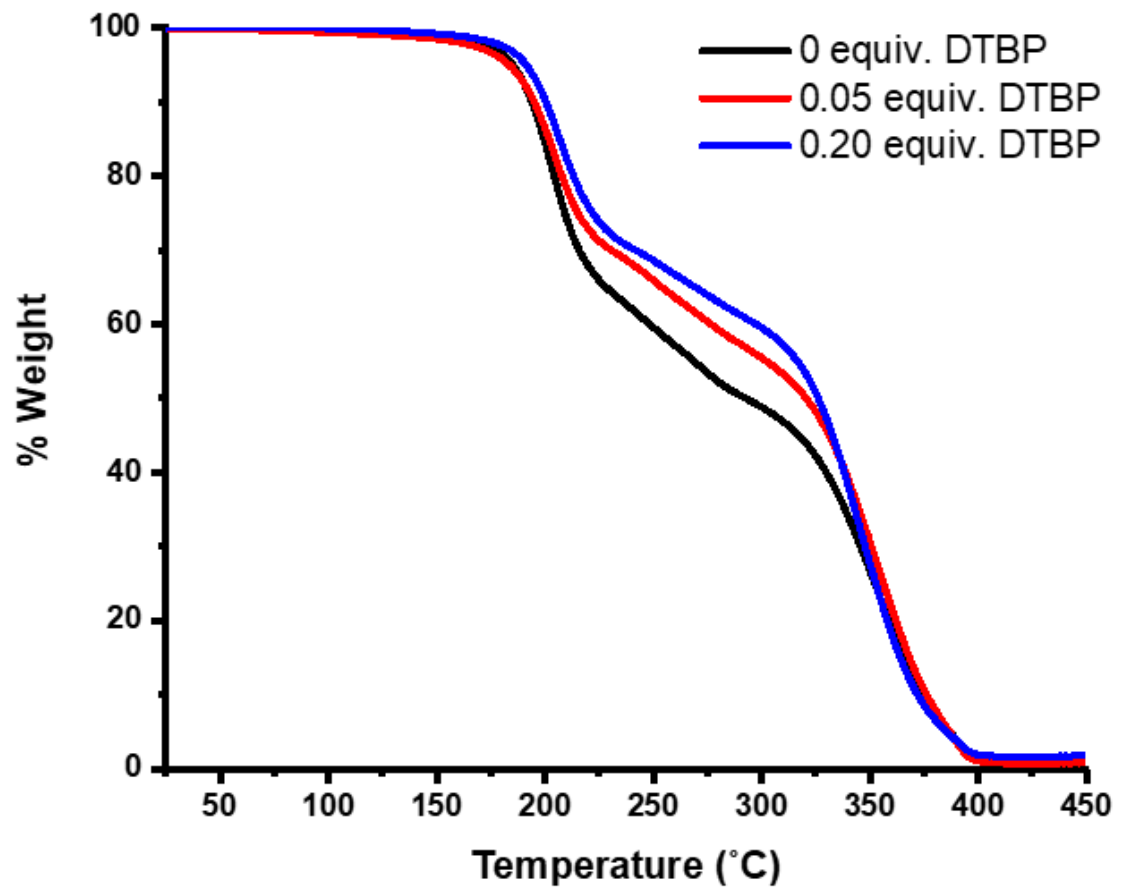

Supplementary Figure 4: TGA data showing the effect of adding a radical initiator to the depolymerization of PMMA in the presence of NHPI. Dynamic experiments were performed with 0.05 equivalents of NHPI and either 0, 0.05 or 0.2 equivalents of DTBP w.r.t. polymer repeat unit concentration.

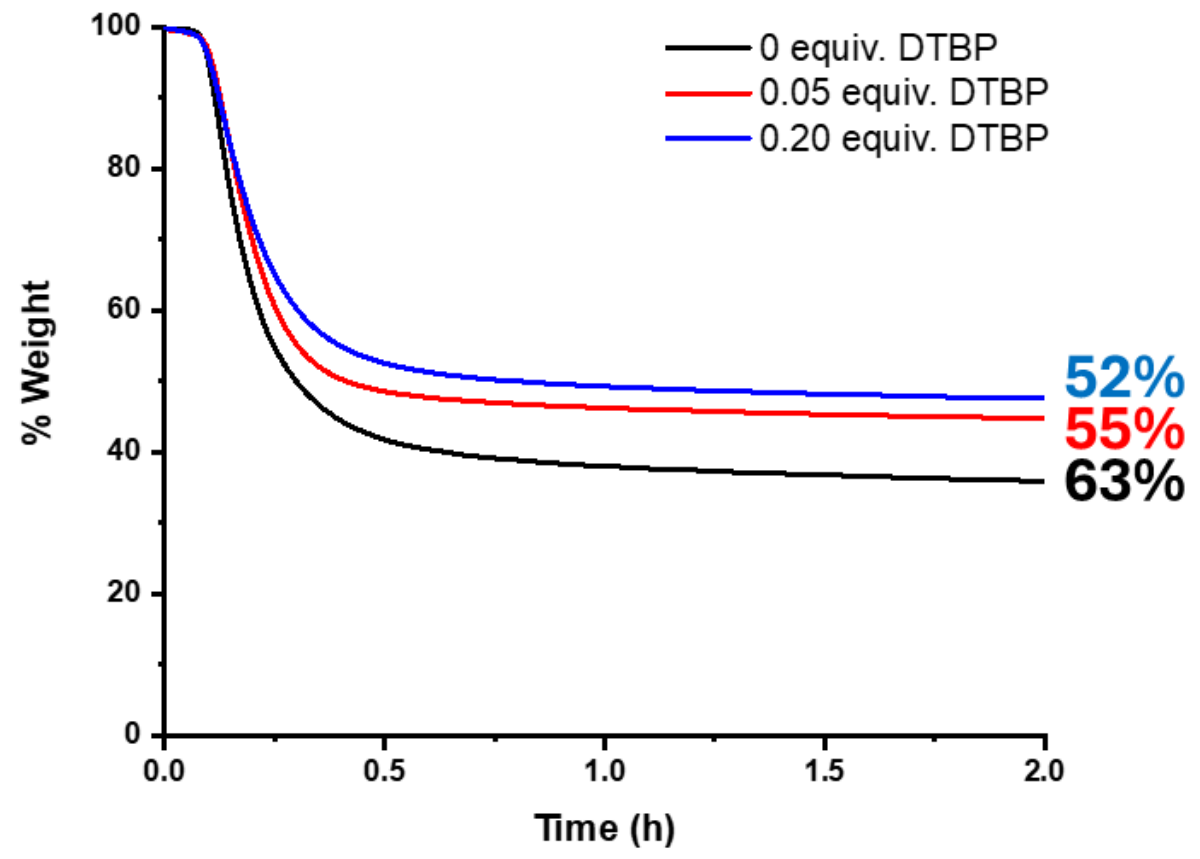

Supplementary Figure 5: TGA data showing the effect of adding a radical initiator to the depolymerization of PMMA in the presence of NHPI. Isothermal experiments were performed at 230 °C with 0.05 equivalents of NHPI and either 0, 0.05 or 0.2 equivalents of DTBP w.r.t. polymer repeat unit concentration.

Supplementary Table 1: TGA data showing the effect of increasing the concentration of NHPI on the depolymerization of PMMA prepared by anionic polymerization. Dynamic experiments were performed with a 1 °C/minute heating rate from 25 °C to 300 °C.

Equivalents of NHPI w.r.t $[RU]_0$	Depolymerization %
0.05	58
0.2	63
0.4	64

Supplementary Table 2: TGA data showing the effect of changing the *N*-hydroxyimide on the extent of depolymerization. Reactions were performed for 2 hours at 230 °C with 0.05 equivalents of *N*-hydroxyimide w.r.t. polymer repeat unit concentration. The molecular weight of the remaining polymer is also shown in column 4.

Additive	Time (h)	Depolymerization %	M_n
-	0	0	715000
NHS	2	35	234000
NHPI	2	63	84000
TCNHPI	2	93	21500

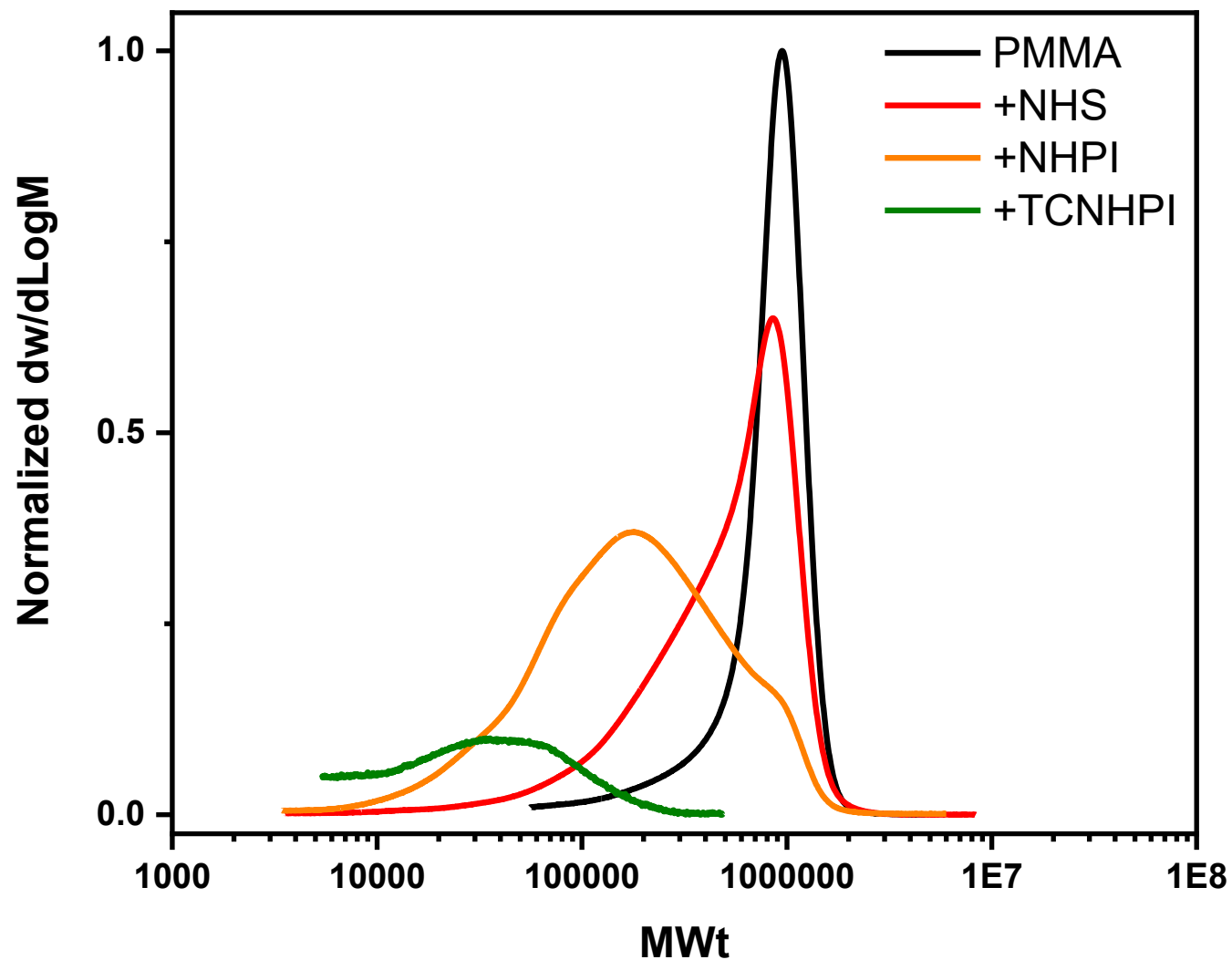

Supplementary Figure 6: SEC data showing the molecular weight distribution of the remaining polymer after depolymerization was complete. Reactions were performed for 2 hours at 230 °C with 0.05 equivalents of the various *N*-hydroxyimide derivatives w.r.t polymer repeat unit.

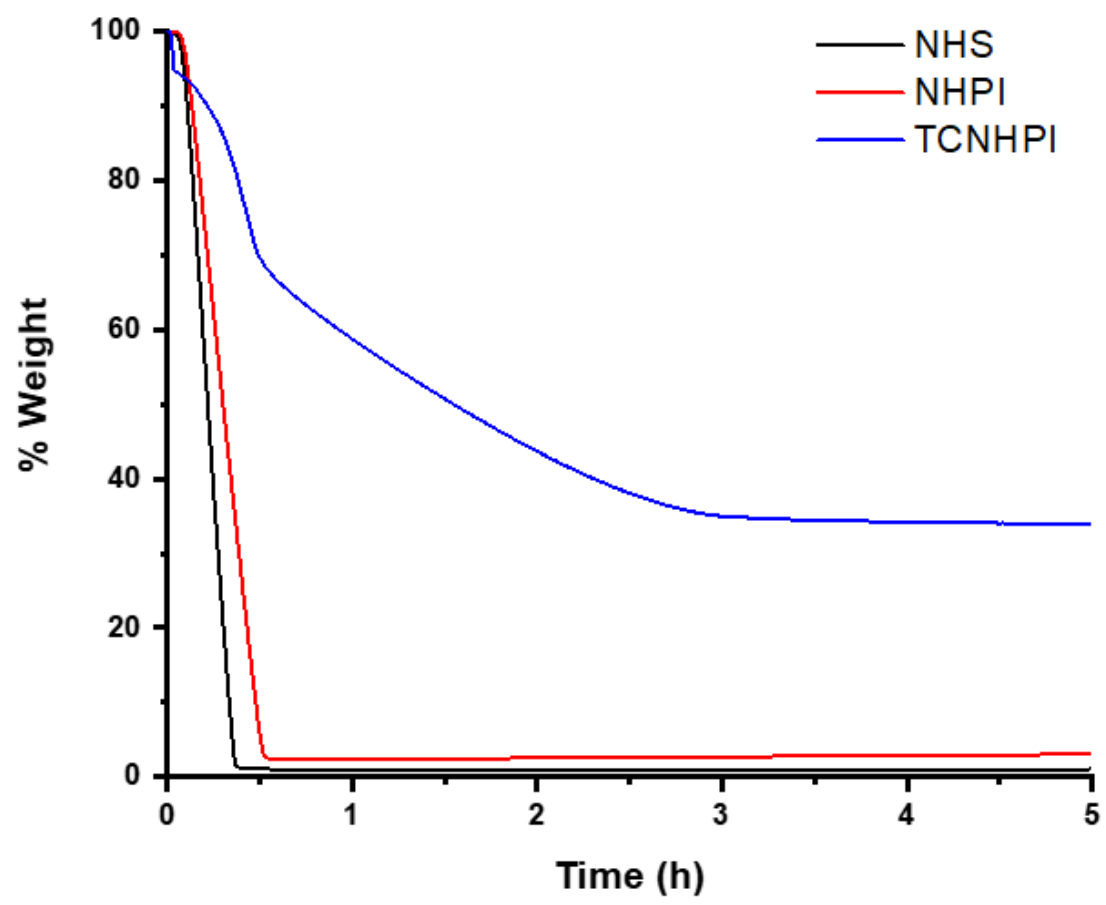

Supplementary Figure 7: TGA data showing the stability of NHS, NHPI and TCNHPI on heating to 230°C for 5 hours.

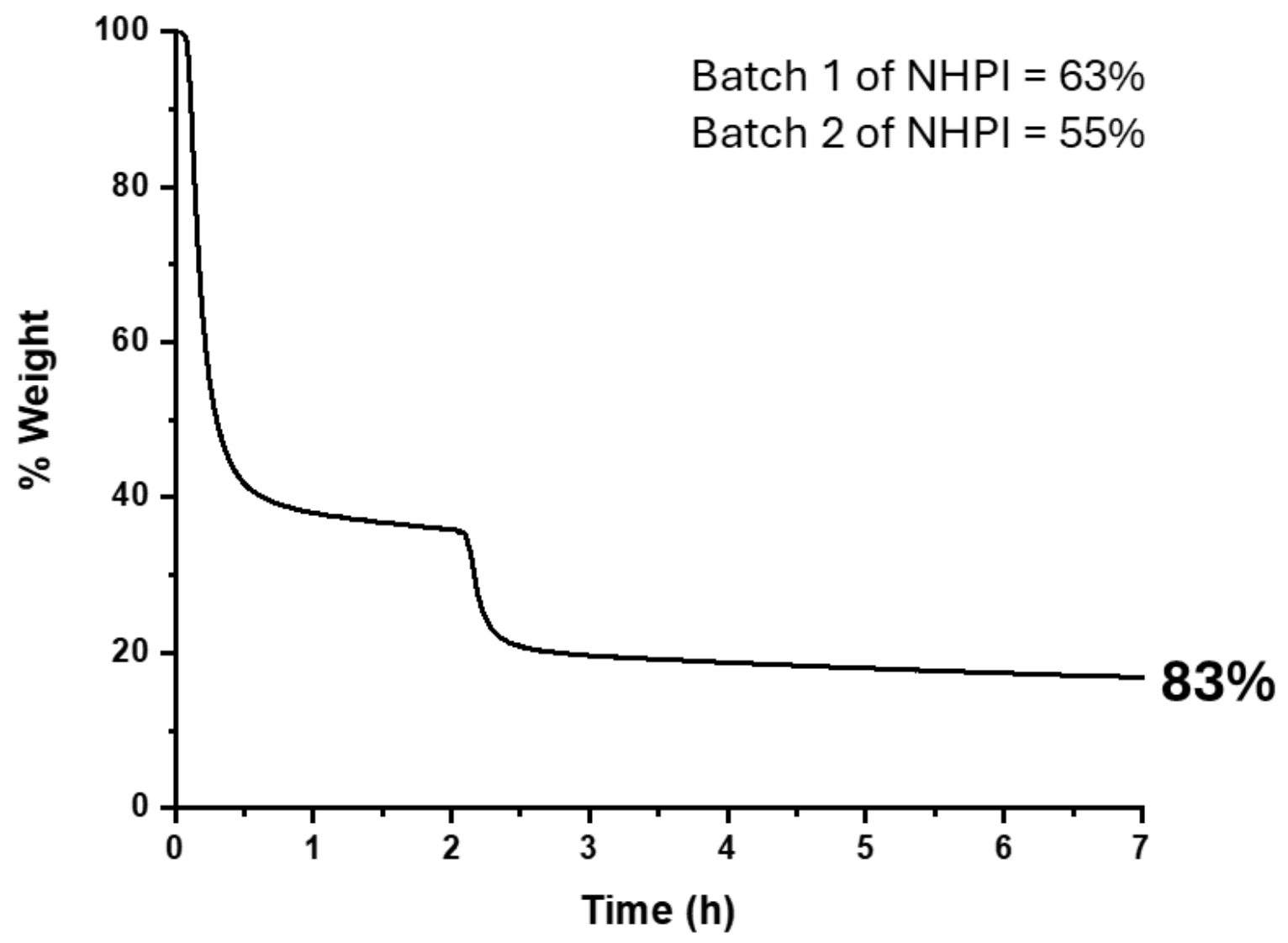

Supplementary Figure 8: TGA data showing the effect of adding a second batch of NHPI after depolymerization plateau. It was possible to achieve an overall yield of 83%. Reactions were performed with 0.05 equivalents of NHPI w.r.t. polymer repeat unit concentration at 230 °C.

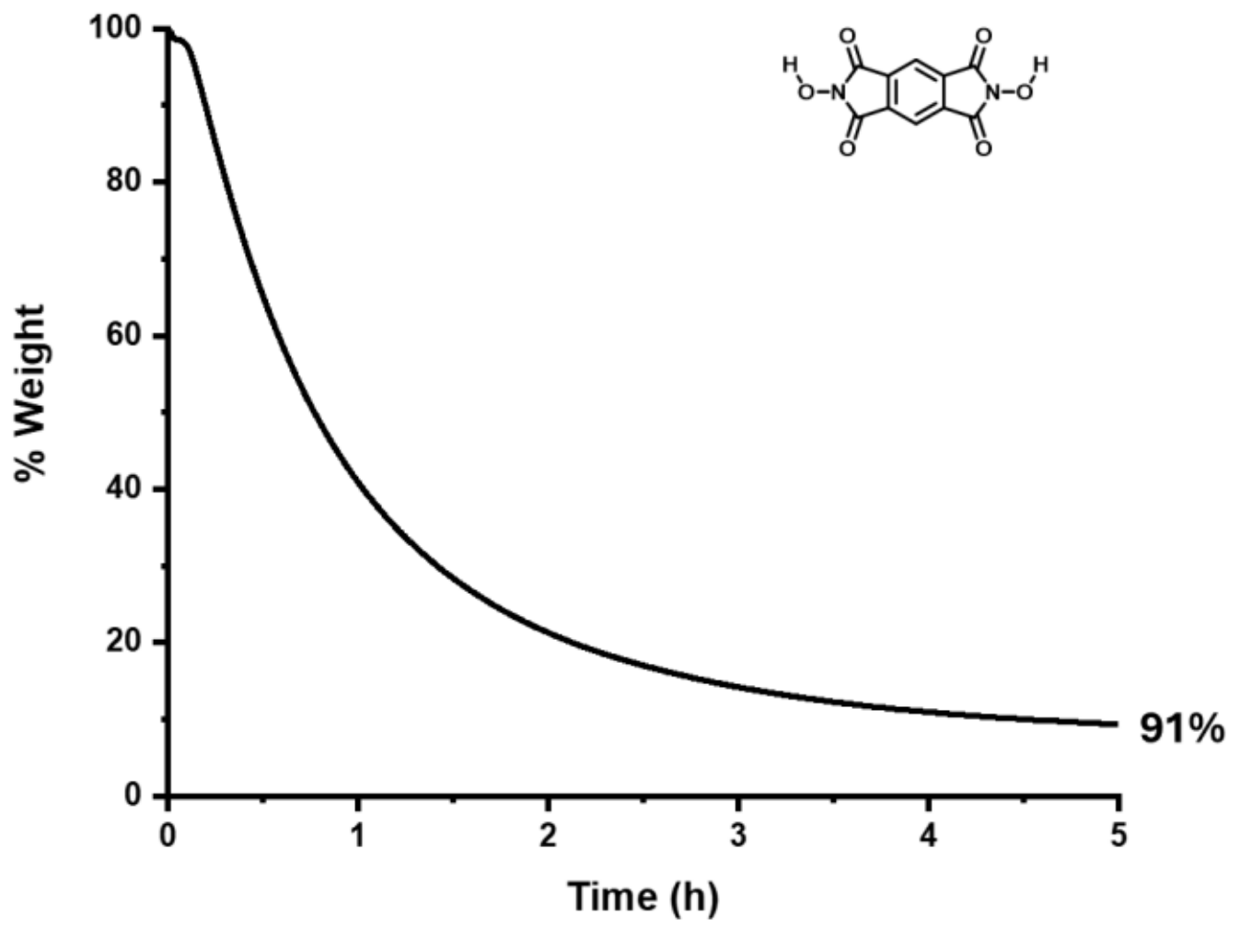

Supplementary Figure 9: TGA data showing for the depolymerization of PMMA using N,N'-Dihydroxypyromellitimide (NDHPI) as the additive. It was possible to achieve an overall yield of 91%. Reactions were performed with 0.025 equivalents of NDHPI w.r.t. polymer repeat unit concentration at 230 °C.

Supplementary Table 3: Table showing the effect of decreasing the temperature on the depolymerization of PMMA prepared by anionic polymerization. Isothermal TGA reactions were performed for 5 hours at various temperatures with 0.05 equivalents of TCNHPI w.r.t polymer repeat unit concentration.

Temperature (°C)	Depolymerization %
170	46
180	70
190	80
200	86
210	88
220	91
230	93

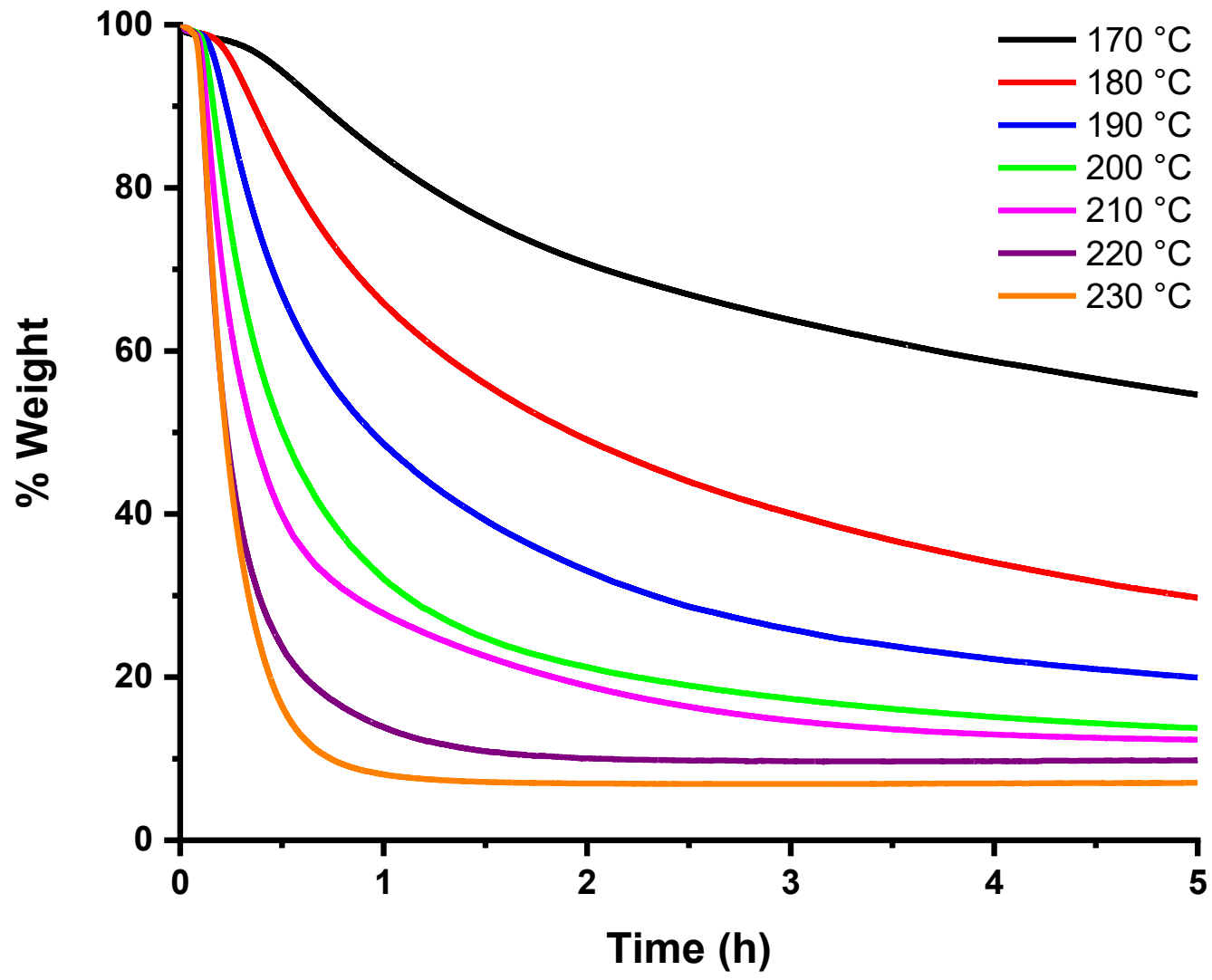

Supplementary Figure 10: TGA data showing the effect of decreasing the temperature on the depolymerization of PMMA prepared by anionic polymerization. Isothermal reactions were performed for 5 hours at various temperatures with 0.05 equivalents of TCNHPI w.r.t. polymer repeat unit concentration.

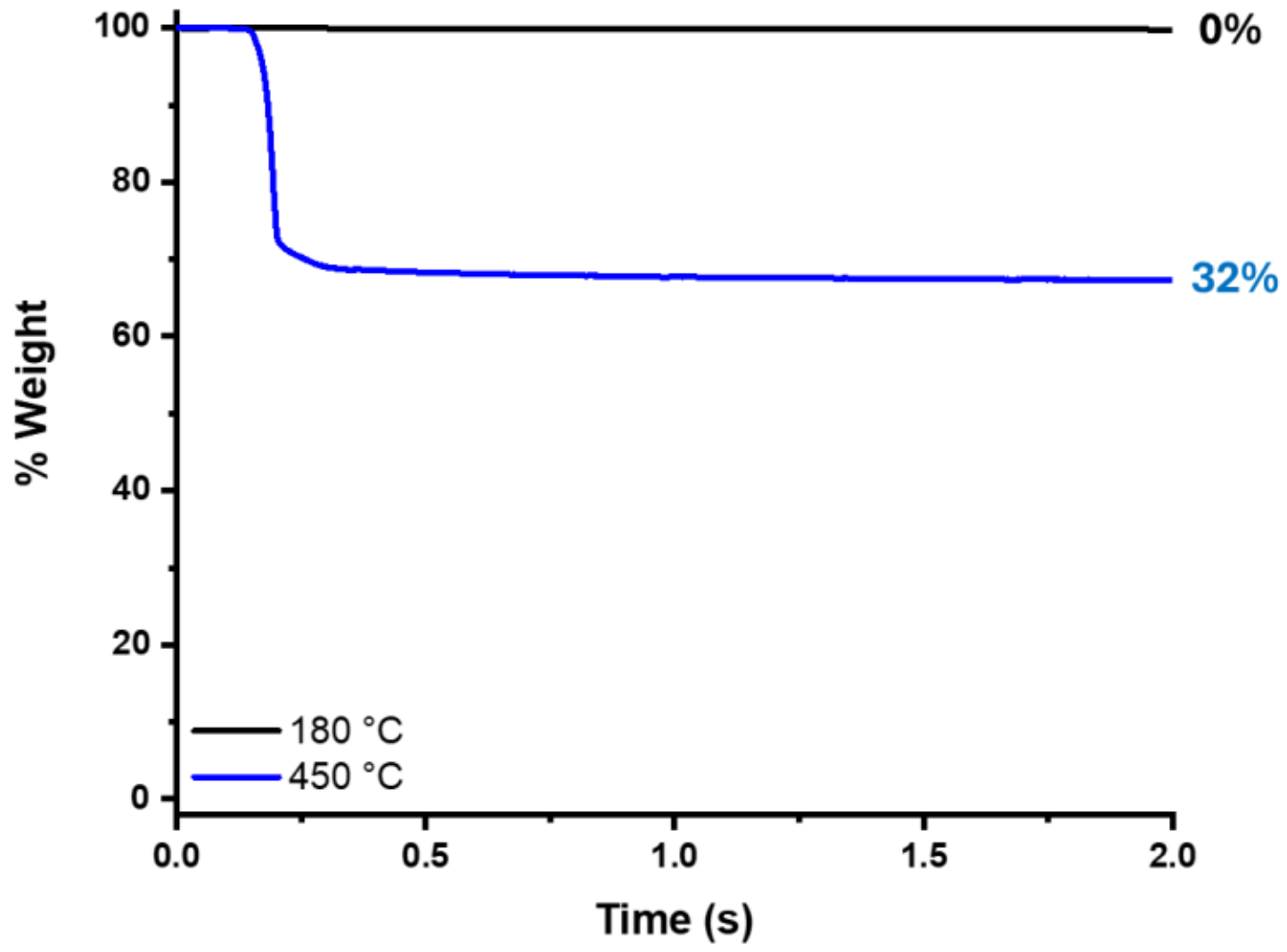

Supplementary Figure 11: TGA data showing the effect of decreasing the temperature on the stability of Al(OH)$_3$. Specifically, decomposition of Al(OH)$_3$ was observed at pyrolysis temperatures but not at the temperature of the HAT triggered depolymerization. Reactions were performed for 2 hours.

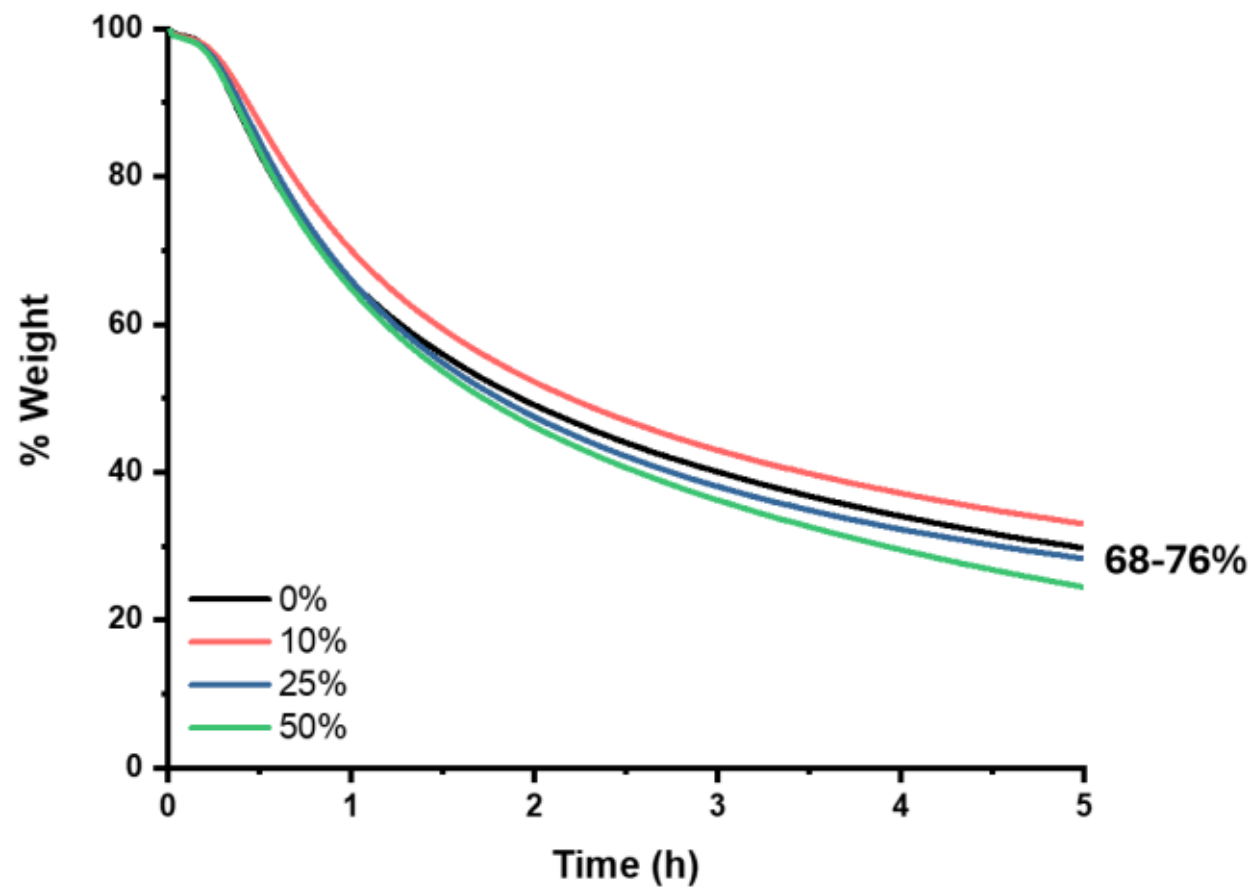

Supplementary Figure 12: TGA data showing the effect of the weight percent of Al(OH)$_3$ on the depolymerization of PMMA. In all cases comparable depolymerization was observed to the reaction performed in the absence of Al(OH)$_3$. Reactions were performed at 180°C for 5 hours with 0.05 equivalents of TCNHPI w.r.t. polymer repeat unit concentration.

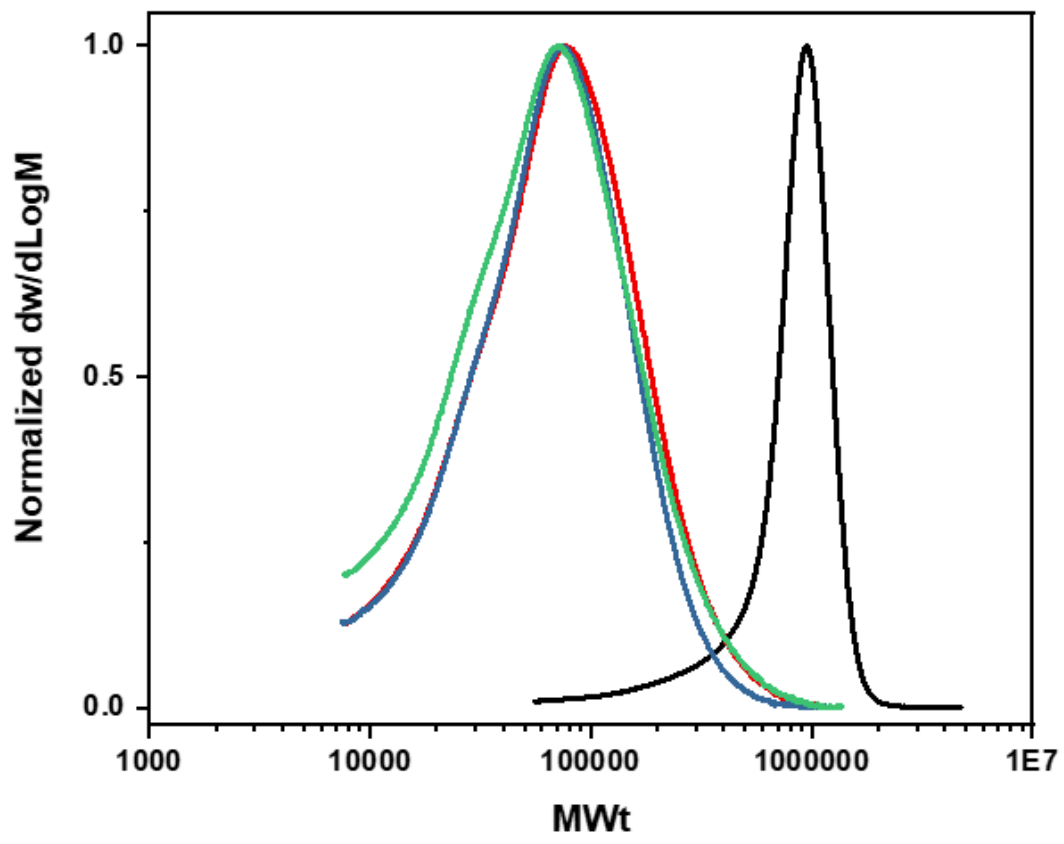

Supplementary Figure 13: SEC data showing the effect of the weight percent of $Al(OH)_3$ on the molecular weight of the unreacted fragments after depolymerization of PMMA. In all cases comparable extents of scission were observed to the reaction performed in the absence of $Al(OH)_3$. Reactions were performed at 180°C for 5 hours with 0.05 equivalents of TCNHPI w.r.t. polymer repeat unit concentration.

Supplementary Table 4: Table showing the effect of decreasing the amount of TCNHPI on the depolymerization of PMMA prepared by anionic polymerization. Isothermal TGA reactions were performed for 2 hours at 230 °C with various amounts of TCNHPI. All amounts are relative to the polymer repeat unit concentration.

Equivalents of TCNHPI	Depolymerization %
0.05	93
0.01	93
0.004	93
0.002	82
0	3

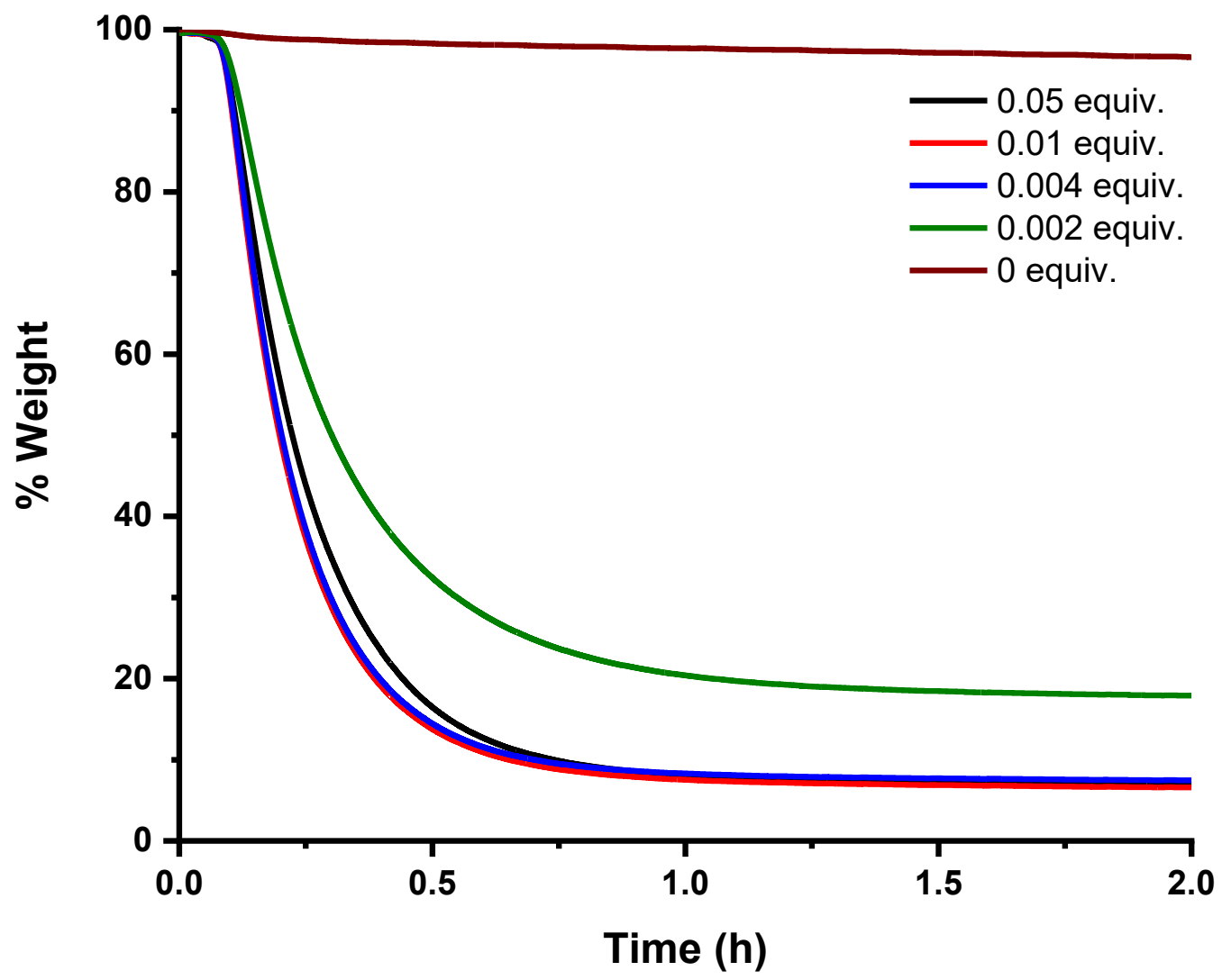

Supplementary Figure 14: TGA data showing the effect of decreasing the amount of TCNHPI on the depolymerization of PMMA prepared by anionic polymerization. Isothermal reactions were performed for 2 hours at 230 °C with various amounts of TCNHPI relative to the polymer repeat unit concentration.

Supplementary Table 5: Table of data obtained from kinetic experiments, illustrating the effect of depolymerization on the molecular weight distribution of the remaining polymer chains. Isothermal TGA reactions were performed at 230 °C for various times using 0.05 equivalents of TCNHPI w.r.t polymer repeat unit concentration and a polymer made by anionic polymerization.

Time of Experiment (seconds)	Depolymerization %	M_n	M_w	*Đ*
0	0	715000	994000	1.39
358	15	150000	403000	2.70
440	30	100400	281500	2.80
552	45	63500	164600	2.59
758	60	46100	102500	2.22
1301	75	28000	51000	1.82
7200	90	21500	47000	2.18

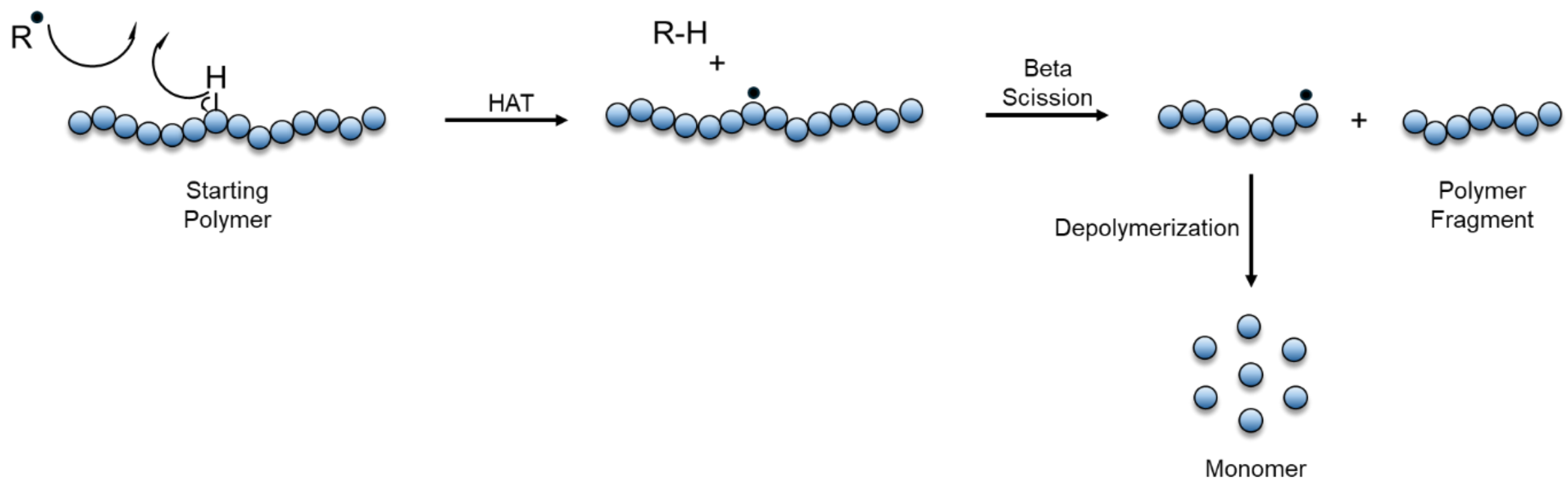

Supplementary Scheme 1: The proposed mechanism of mid-chain scission, triggered by hydrogen abstraction from the polymer backbone. Two polymer fragments are formed, one of which contains a radical that can depropogate and one does not, so requires a subsequent activation step for further depolymerization to be achieved.

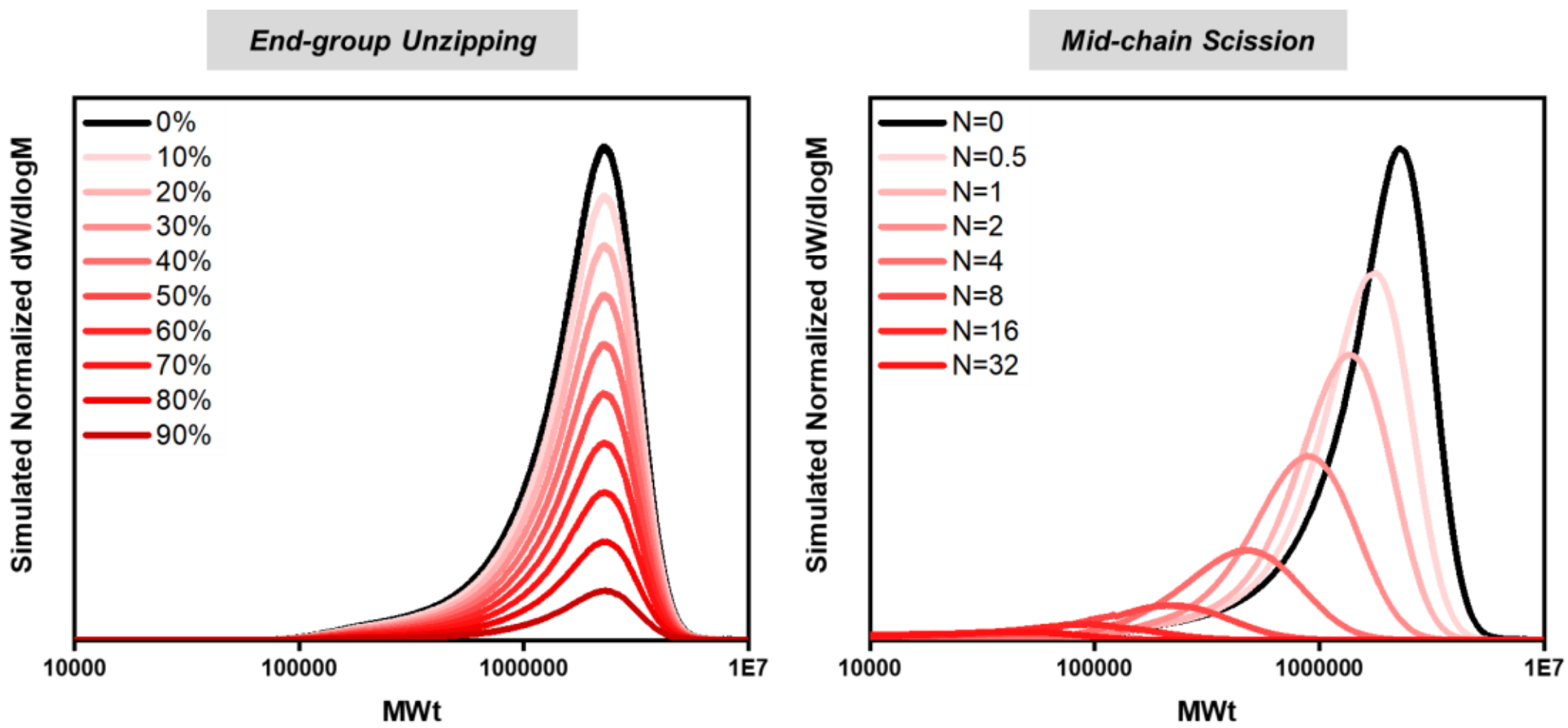

Supplementary Figure 15: Simulated SEC traces illustrating the theoretical evolution of the molecular weight distribution of the polymer for either an end-group or mid-chain initiated depolymerization process. Each trace corresponds to a depolymerization conversion (left) or number of scission events (N, right). This simulation was performed using MatLab R2024b.

Mechanism of Mid-Chain Bulk Depolymerization

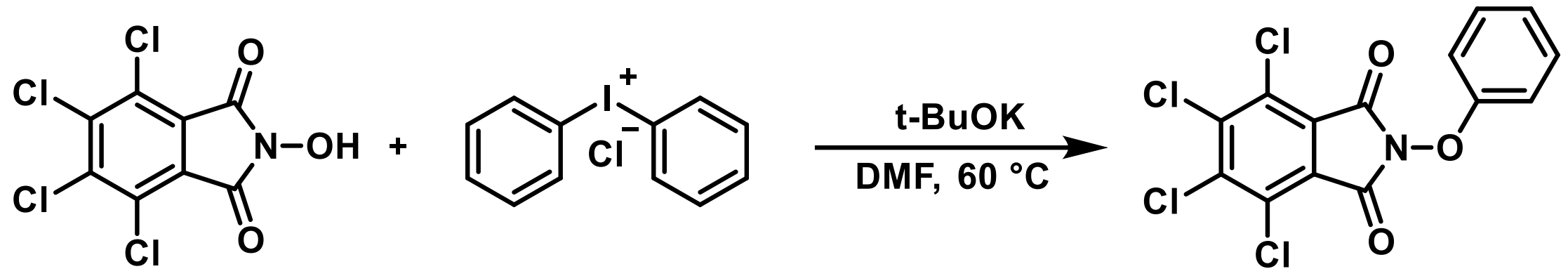

Supplementary Scheme 2: A scheme showing the synthetic procedure for the preparation of *N*-phenoxytetrachlorophthalimide (TCNPhPI, **6**).

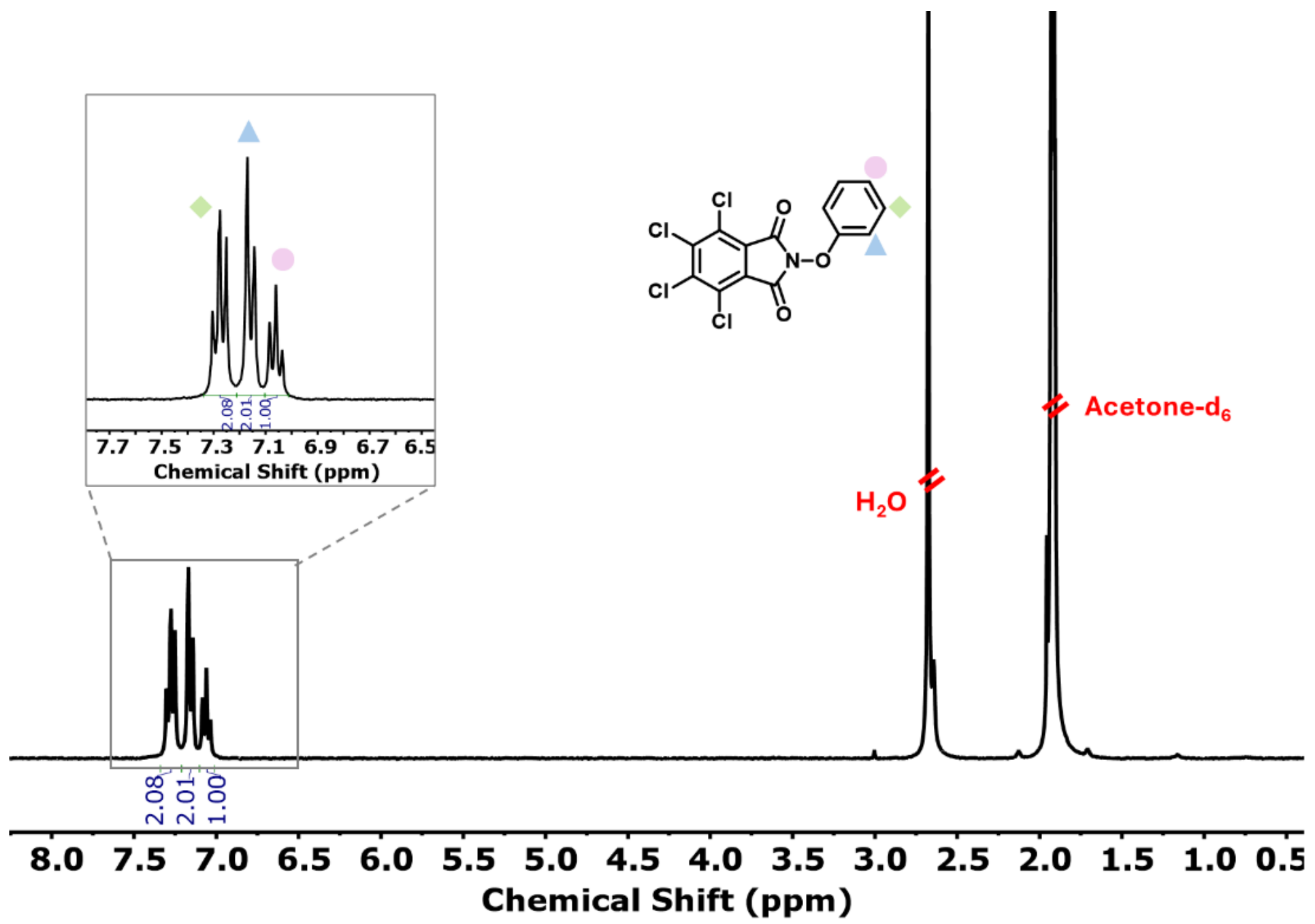

Supplementary Figure 16: ^{1}H NMR of *N*-phenoxytetrachlorophthalimide (TCNPhPI, **6**).

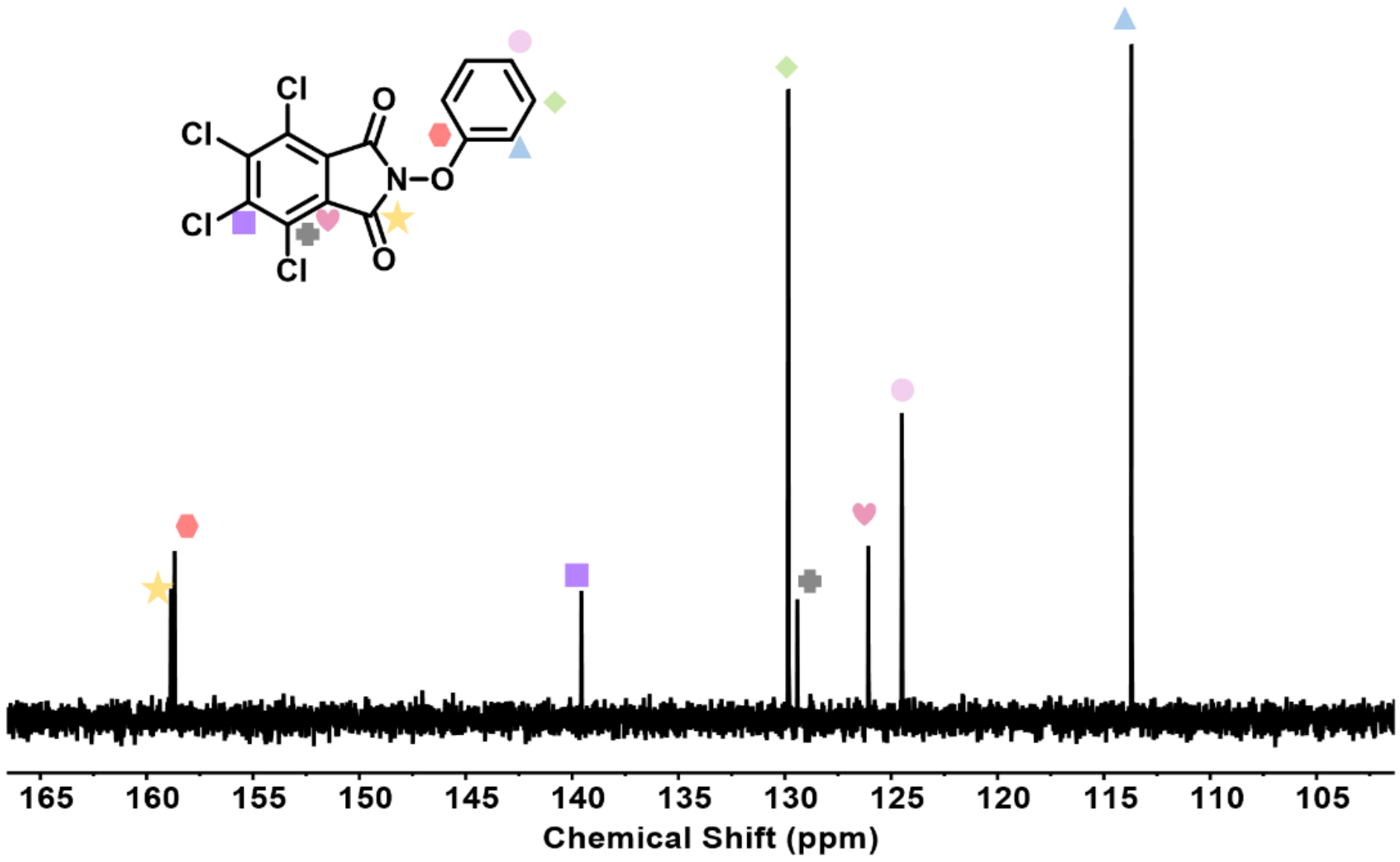

Supplementary Figure 17: ^{13}C NMR of *N*-phenoxytetrachlorophthalimide (TCNPhPI, **6**).

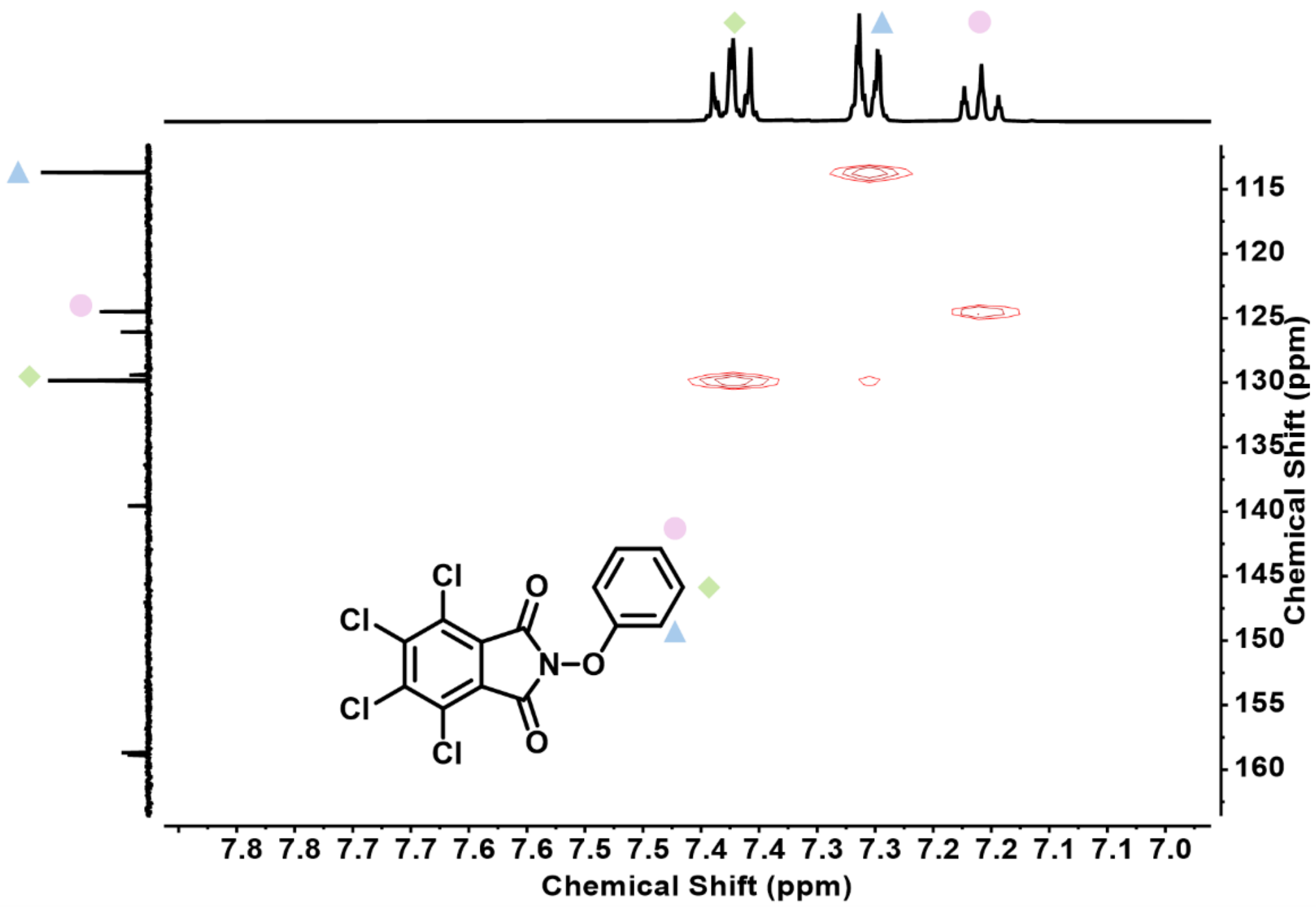

Supplementary Figure 18: HSQC NMR of *N*-phenoxytetrachlorophthalimide (TCNPhPI, **6**).

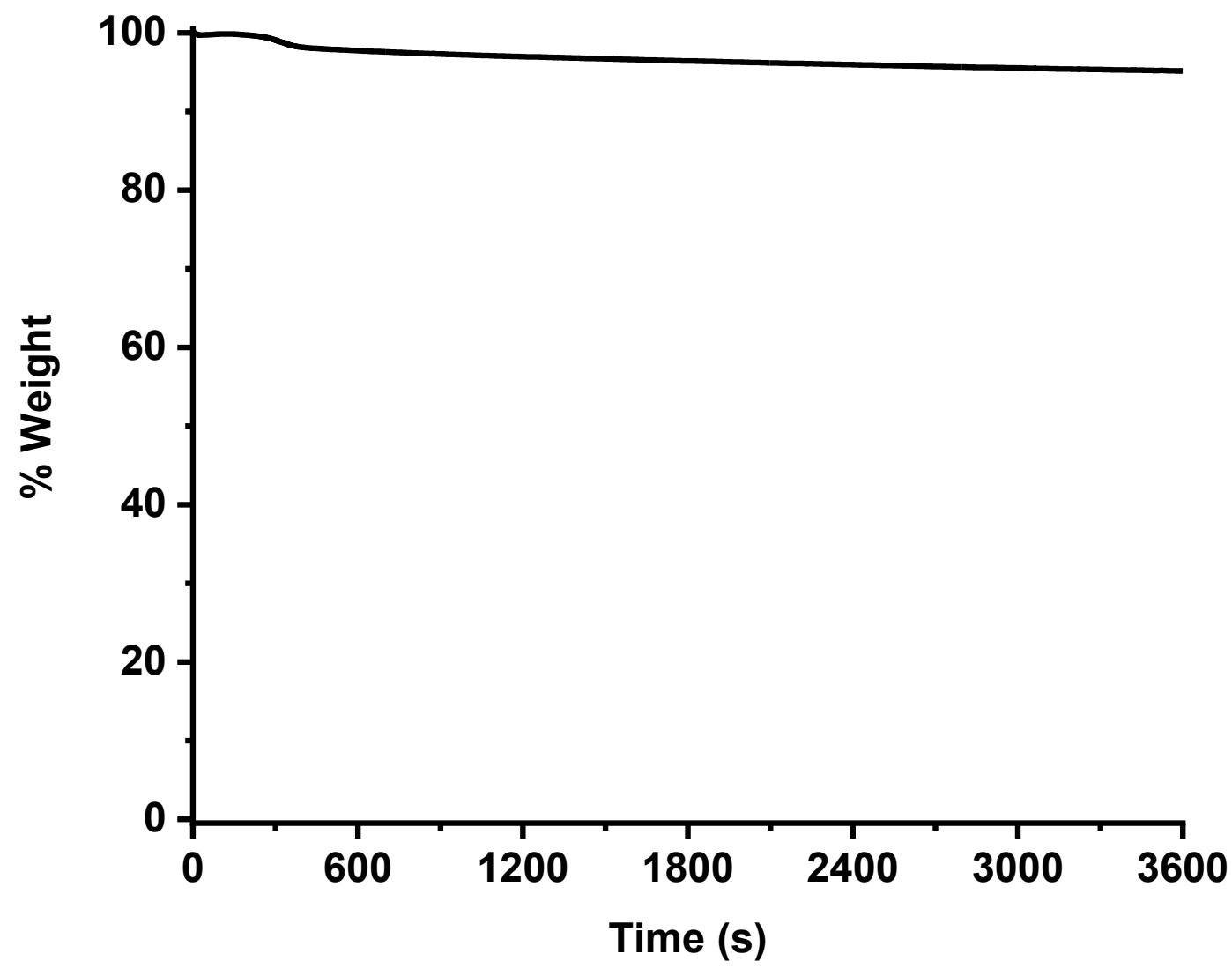

Supplementary Figure 19: TGA data showing the effect of using TCNPhPI on the depolymerization of PMMA prepared by anionic polymerization. Isothermal reaction was performed for 1 hour at 230 °C with 0.05 equivalents of TCNPhPI relative to the polymer repeat unit concentration. Negligible depolymerization was observed.

Supplementary Table 6: Standard enthalpy of formation used for the DFT calculations.

Atom	H_f (kJ mol^{-1})
H atom	218.0
Cl atom	121.7
H_2O_2	-187.8

Supplementary Table 7. Cartesian coordinates of DFT models used for bond dissociation energy calculations.

NHPI (–588.0235781 Hartree)				NHS (–435.6540802 Hartree)			
C	1.681027	1.421683	-0.002218	C	-1.183348	-0.109435	-0.001428
C	0.503821	0.696431	-0.004220	O	-2.288698	0.360978	-0.006672
C	0.503820	-0.696438	-0.004221	N	-0.000345	0.626305	-0.021712
C	1.681026	-1.421691	-0.002108	C	1.183720	-0.107876	-0.002353
C	2.877115	-0.698401	0.004886	O	2.288527	0.363699	-0.006717
C	2.877115	0.698393	0.004824	O	-0.001152	1.981149	0.126058
H	1.665401	2.506223	-0.002273	H	-0.007460	2.347816	-0.770125
H	1.665399	-2.506231	-0.002189	C	0.769833	-1.571630	0.001888

H	3.823520	-1.228787	0.011364
H	3.823521	1.228779	0.011292
C	-0.911172	-1.179014	-0.011011
O	-1.347411	-2.297961	0.027917
N	-1.670041	0.000012	-0.101734
C	-0.911172	1.179006	-0.010614
O	-1.347384	2.297980	0.027850
O	-3.012838	-0.000015	0.140695
H	-3.435969	0.000091	-0.729657

NHPI_O_radical (–587.3878120 Hartree)

C	1.635045	-1.421613	0.000025
C	0.454416	-0.697635	-0.000154
C	0.454417	0.697635	-0.000133
C	1.635045	1.421613	-0.000006
C	2.829365	0.699243	0.000133
C	2.829364	-0.699243	0.000156
H	1.619321	-2.506054	0.000009
H	1.619321	2.506054	0.000009
H	3.775787	1.229388	0.000247
H	3.775787	-1.229388	0.000268
C	-0.944945	1.196338	-0.000205
O	-1.381356	2.309966	-0.000059
N	-1.753964	0.000000	0.000034
C	-0.944945	-1.196338	-0.000443
O	-1.381356	-2.309966	0.000017
O	-3.012167	0.000000	0.000416

NHPI_N_radical (–512.1943075 Hartree)

C	1.325642	-1.424671	0.000213
C	0.145596	-0.694646	0.000314
C	0.145597	0.694707	0.000383
C	1.325633	1.424734	0.000147
C	2.516875	0.701300	-0.000228
C	2.516879	-0.701234	-0.000172
H	1.310885	-2.509108	0.000228
H	1.310872	2.509171	0.000296
H	3.465097	1.228540	-0.000643
H	3.465100	-1.228477	-0.000601
C	-1.276180	1.146156	0.000256
O	-1.695816	2.277649	-0.000035
N	-2.137684	-0.000012	-0.000460
C	-1.276157	-1.146211	-0.000528
O	-1.695619	-2.277755	0.000239

TCNHPI_2C_radical (–1966.0198215 Hartree)

C	-0.467835	0.995281	0.003175
C	0.752759	0.339665	0.001623
C	0.827396	-1.058262	0.004068

H	1.201094	-2.045228	0.885993
H	1.203885	-2.057714	-0.873925
C	-0.767706	-1.572645	0.001603
H	-1.200804	-2.058619	-0.874754
H	-1.198695	-2.047472	0.885175

NHS_O_radical (–435.0138976 Hartree)

C	1.196740	0.072172	0.000761
O	2.295402	-0.398470	-0.000095
N	0.000000	-0.717771	0.000095
C	-1.204817	0.074521	C
O	-2.295403	-0.398471	-0.000057
O	0.000000	-1.977880	-0.000195
C	-0.769148	1.526057	0.000153
H	-1.197807	2.010879	0.879742
H	-1.198478	2.011232	-0.878903
C	0.769148	1.526056	-0.000390
H	1.197783	2.010313	-0.880304
H	1.198503	2.011795	0.878340

NHS_N_radical (–359.8190809 Hartree)

C	-1.132917	0.244642	-0.000072
O	-2.257573	0.697080	0.000597
N	-0.000002	1.085658	-0.001078
C	1.132926	0.244658	-0.000117
O	2.257594	0.697049	0.000631
C	0.763643	-1.237301	-0.000099
H	1.205576	-1.710174	0.879712
H	1.206698	-1.714759	H
C	-0.763669	-1.237307	-0.000047
H	-1.205587	-1.710218	-0.879850
H	-1.205526	-1.710190	0.879796

TCNHPI (–2426.2712771 Hartree)

C	0.366920	1.419859	-0.003366
C	-0.811320	0.696761	-0.000537
C	-0.811317	-0.696770	-0.000546
C	0.366928	-1.419862	-0.003307
C	1.578236	-0.702154	0.001234
C	1.578232	0.702159	0.001192
C	-2.233411	-1.179299	-0.008897
O	-2.677408	-2.291424	0.040914
N	-2.983973	0.000004	-0.115656
C	-2.233416	1.179283	-0.008617
O	-2.677395	2.291429	0.040884
O	-4.338849	0.000003	O
H	-4.757159	0.000056	-0.723185
Cl	0.350576	3.137572	-0.009979
Cl	3.068768	1.558550	0.006438

C	-0.330514	-1.775128	0.006987
C	-1.572818	-1.179684	0.001767
C	-1.650927	0.224657	0.000167
C	2.267026	-1.476538	0.012780
O	2.750575	-2.572190	-0.029302
N	2.958921	-0.262251	0.109057
C	2.152719	0.884564	0.007515
O	2.549702	2.014116	-0.039901
O	4.297151	-0.195608	-0.141538
H	4.726203	-0.153194	0.725377
Cl	-0.537736	2.713359	0.006414
Cl	-3.184709	0.998764	-0.005697
Cl	-2.988433	-2.165588	-0.002560

TCNHPI_O_radical (–2425.6333635 Hartree)			
C	-0.332526	-1.420504	0.000093
C	0.848784	-0.698314	0.000118
C	0.848784	0.698314	0.000171
C	-0.332525	1.420503	-0.000005
C	-1.543069	0.702869	-0.000074
C	-1.543070	-0.702869	0.000009
C	2.255131	1.196719	0.000584
O	2.700722	2.304114	-0.000144
N	3.054863	0.000000	-0.000060
C	2.255131	-1.196720	-0.000058
O	2.700722	-2.304114	0.000086
O	4.312345	0.000000	-0.000424
Cl	-0.316107	-3.136175	0.000038
Cl	-3.031958	-1.557623	-0.000045
Cl	-3.031958	1.557624	-0.000127
Cl	-0.316106	3.136175	0.000090

Hydrolyzed TCNHPI (–2502.6776800 Hartree)			
C	-1.444815	-0.982326	-0.043199
C	-0.108899	-1.384080	-0.150548
C	0.906997	-0.435146	-0.187192
C	0.593149	0.923457	-0.138160
C	-0.735946	1.335884	-0.020975
C	-1.758914	0.382093	0.026470
Cl	-1.124962	3.012087	0.134566
Cl	-3.399643	0.879064	0.182068
Cl	-2.697772	-2.158418	0.008657
Cl	0.299141	-3.058004	-0.232961
C	1.744532	1.882991	-0.154191
O	2.714604	1.753932	0.562854
C	2.338741	-0.881182	-0.437960
O	2.764144	-1.016003	-1.565111
O	1.627625	2.853131	-1.049659

Cl	3.068777	-1.558537	0.006479
Cl	0.350596	-3.137575	-0.009985

TCNHPI_3C_radical (–1966.0214153 Hartree)			
C	1.280867	-0.672084	0.003066
C	-0.106396	-0.645459	0.001158
C	-0.834703	0.548577	0.000471
C	-0.192033	1.779260	0.001343
C	1.184089	1.713060	-0.003130
C	1.946124	0.572992	-0.002854
C	-2.299637	0.224662	0.009087
O	-3.251993	0.950966	-0.039797
N	-2.331022	-1.172619	0.115181
C	-1.076880	-1.792308	0.009825
O	-0.901629	-2.976546	O
O	-3.475511	-1.867363	-0.141156
H	-3.843331	-2.097265	0.724398
Cl	2.158332	-2.147442	0.009726
Cl	3.666537	0.687019	-0.009472
Cl	-1.023209	3.287250	0.006225

TCNHPI_N_radical (–2350.4419857 Hartree)			
C	-0.091621	1.422261	-0.000311
C	1.088321	0.694879	-0.000267
C	1.088306	-0.694916	-0.000327
C	-0.091655	-1.422279	-0.000262
C	-1.299557	-0.704756	-0.000396
C	-1.299535	0.704774	-0.000450
C	2.518511	-1.145197	-0.000417
O	2.946021	-2.271609	0.000321
N	3.368632	-0.000025	0.000279
C	2.518530	1.145131	0.000214
O	2.969704	2.255372	O
Cl	-0.071130	3.138715	0.000125
Cl	-2.790710	1.557114	0.000113
Cl	-2.790779	-1.557022	0.000199
Cl	-0.071208	-3.138733	0.000060

HTCPINO (–2502.0448143 Hartree)			
C	-1.556948	-0.834461	0.030621
C	-0.281075	-1.404488	-0.060845
C	0.842558	-0.587445	-0.170707
C	0.703240	0.804266	-0.157254
C	-0.567467	1.376871	-0.096136
C	-1.700918	0.557200	-0.005555
Cl	-0.774086	3.087860	-0.200291
Cl	-3.271077	1.256715	0.061779
Cl	-2.944387	-1.840111	0.174452
Cl	-0.105033	-3.120508	0.007224

H	2.419929	3.410522	-0.983927	C	1.968796	1.611604	-0.250654
N	3.058978	-1.145887	0.678894	O	2.861274	1.328168	-1.009963
H	4.055210	-1.301181	0.585575	C	2.191889	-1.239170	-0.351027
O	2.627267	-0.700750	1.908028	O	2.480057	-1.976617	-1.257468
H	2.728833	0.268857	1.862117	O	2.018247	2.618706	0.617427
				H	2.697387	3.207004	H
				N	3.164259	-0.950523	0.646094
				H	4.101779	-1.331587	0.484842
				O	2.913894	-0.334215	1.726366

Supplementary Table 8: DFT calculations showing the bond dissociation energies of various bonds found within the N-hydroxyimide derivatives.

Temperature (°C)	Bond	BDE (kcal mol^{-1})
NHS	N-O	82.38
	O-H	85.18
NHPI	N-O	78.86
	O-H	82.46
TCNHPI	N-O	78.84
	O-H	83.59
	C-Cl	97.11/96.22
TCNPhPI	N-O	50.69

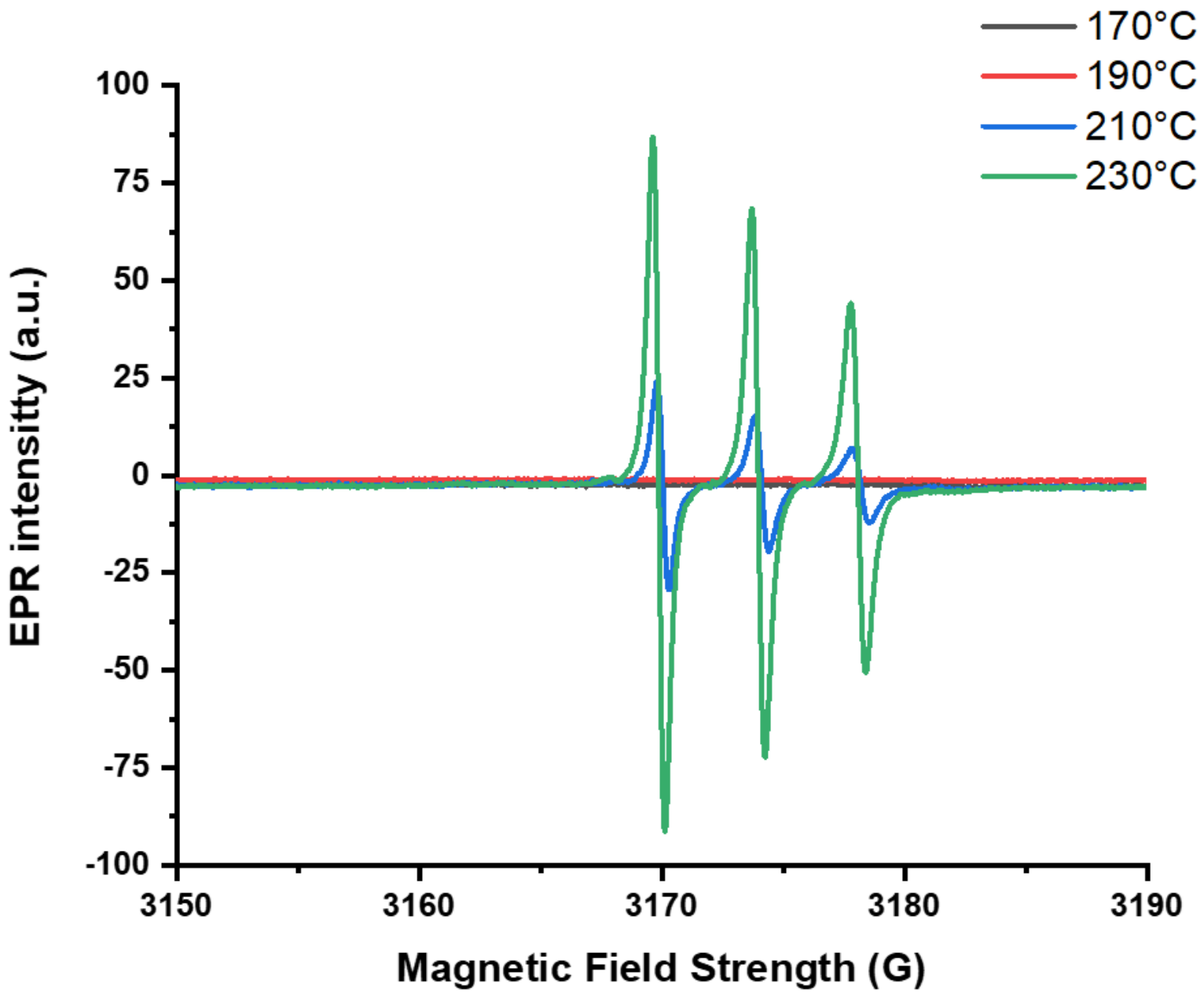

Supplementary Figure 20: EPR data after TCNHPI had been heated to various temperatures. Signal was only observed at 210 °C and 230 °C.

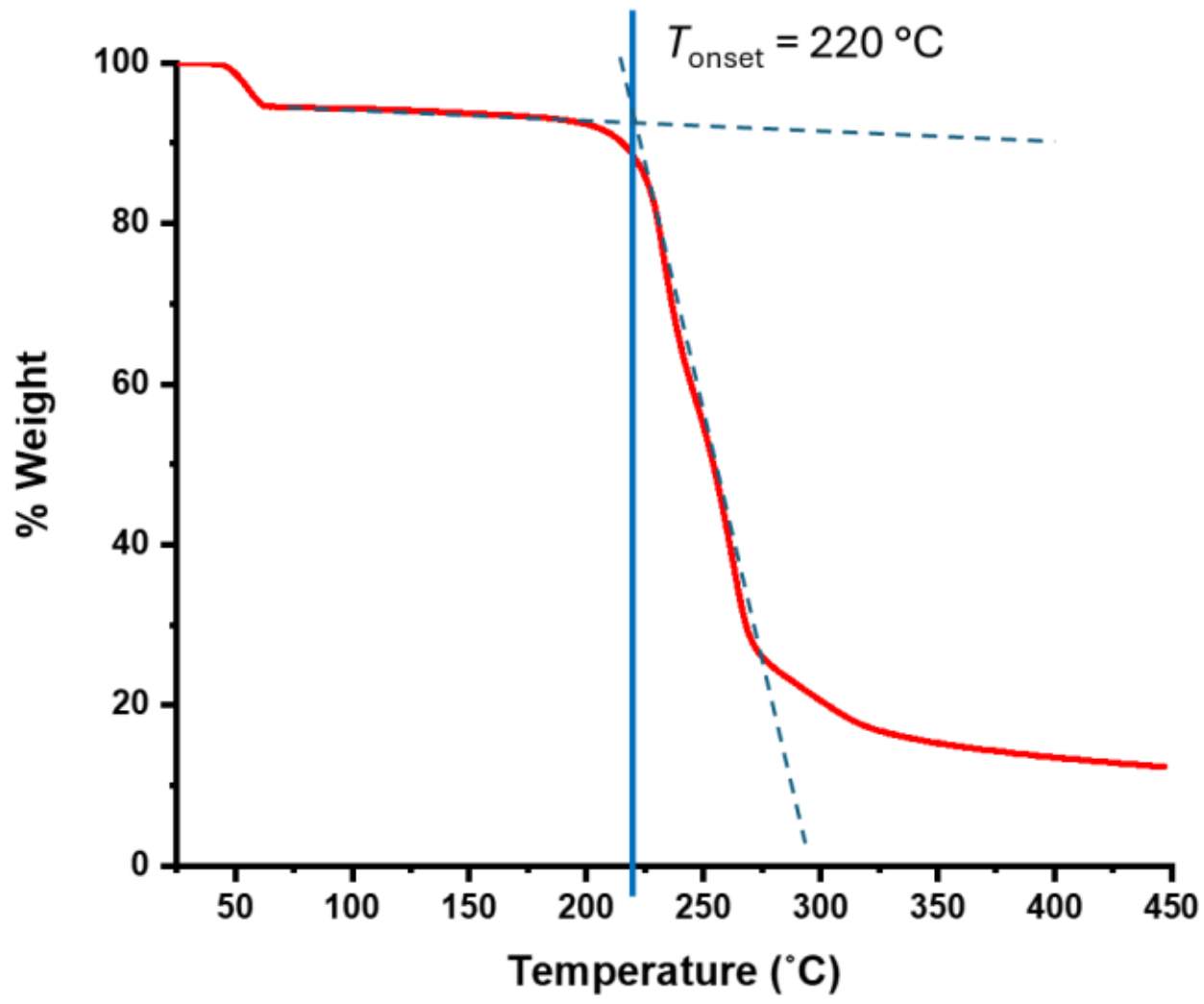

Supplementary Figure 21: TGA data showing the thermal decomposition of TCNHPI. The dotted blue lines denote the values for determining the T_{onset} (solid blue line). Dynamic reaction was performed on heating TCNHPI alone at 1°C/minute from 25 to 450°C

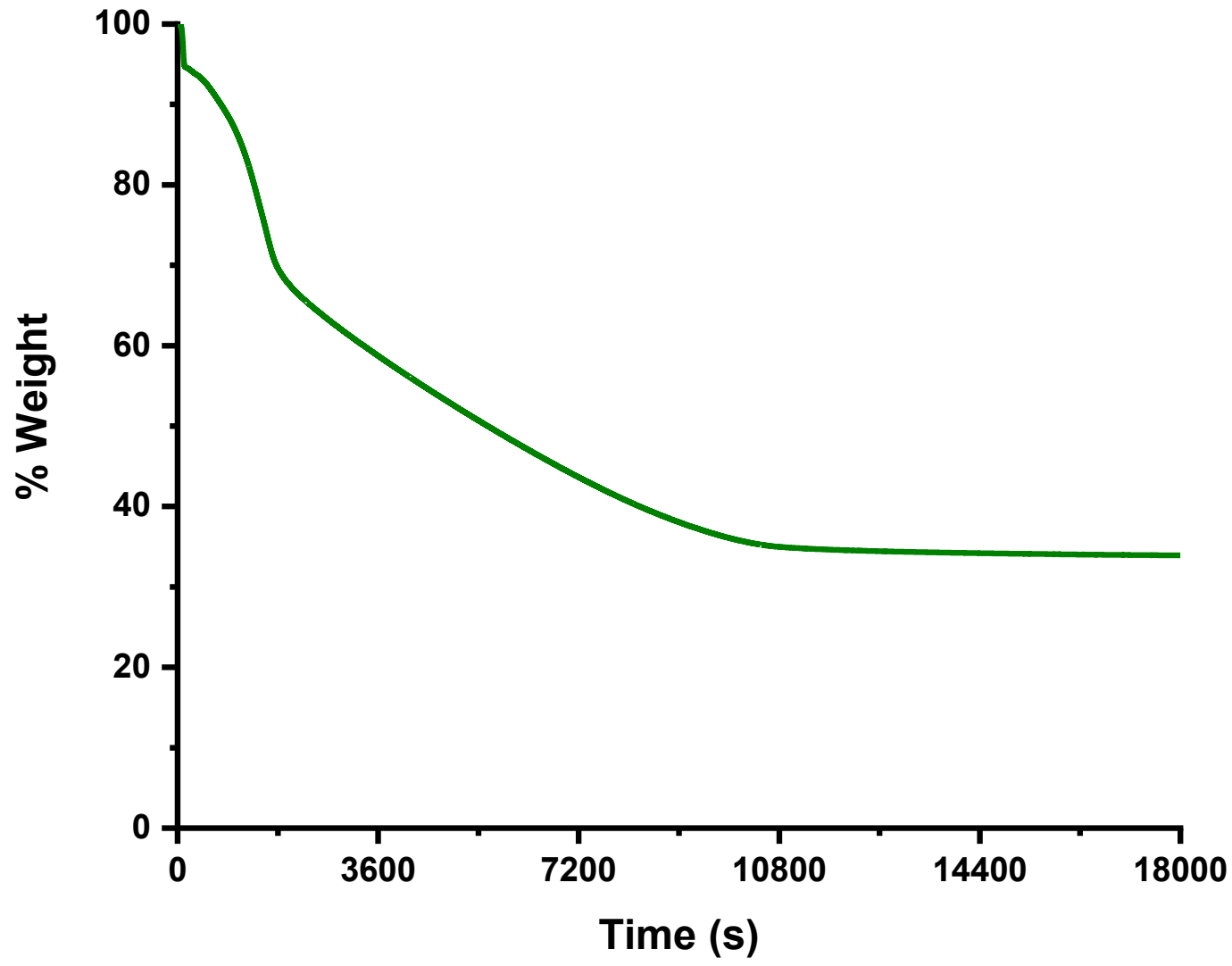

Supplementary Figure 22: TGA data showing the thermal decomposition of TCNHPI. Isothermal reaction was performed for 5 hours at 230 °C with TCNHPI alone.

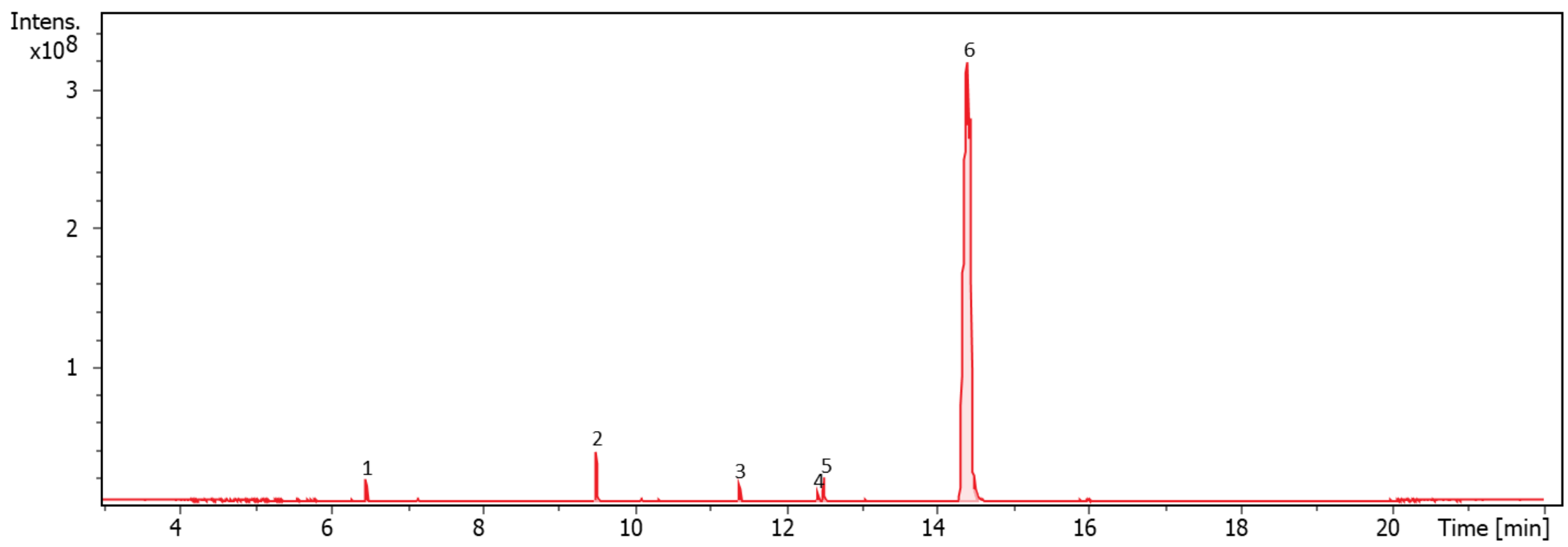

Supplementary Figure 23: GC of the products of the thermal decomposition of TCNHPI. This was a sample obtained after isothermal heating in the TGA at 230 °C for 20 minutes.

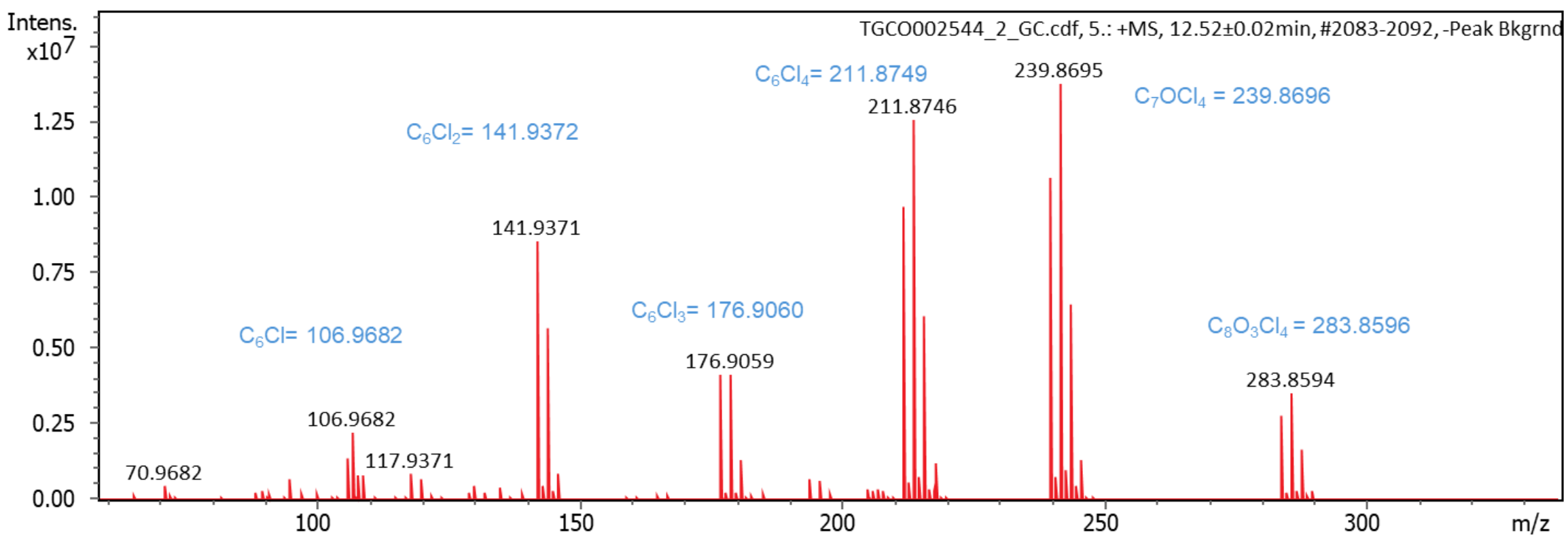

Supplementary Figure 24: MS of product 5 (Supplementary Figure 23) generated from the thermal decomposition of TCNHPI.

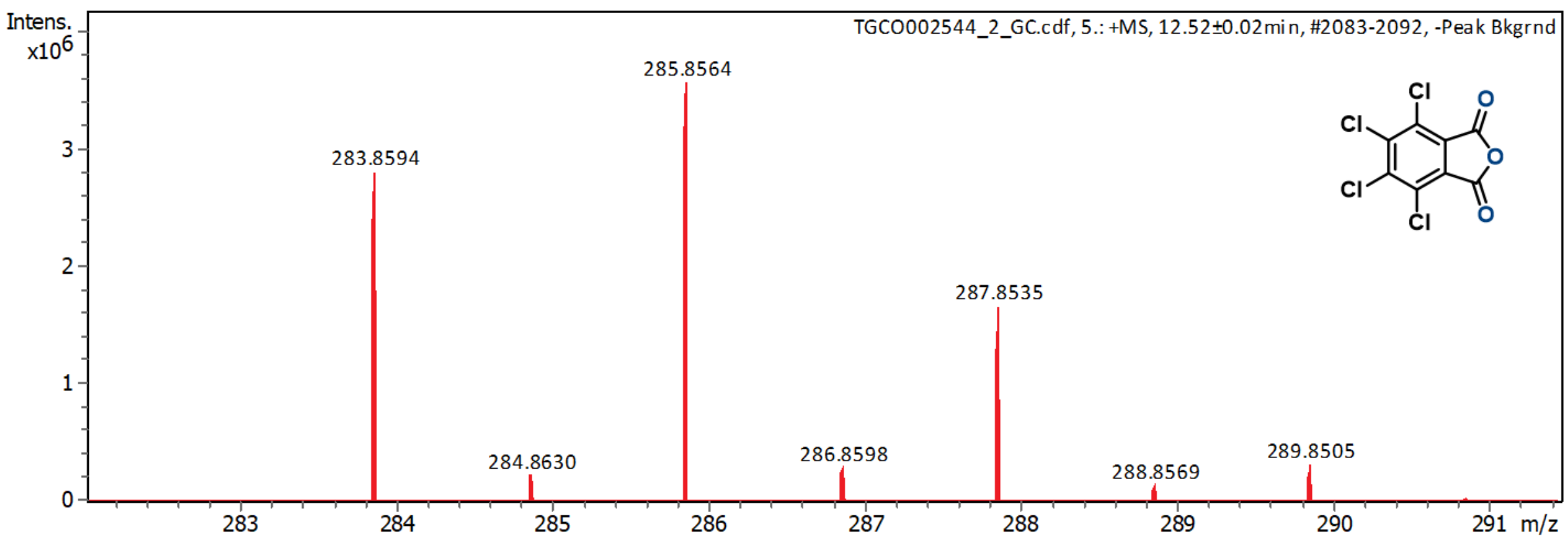

Supplementary Figure 25: Zoomed in MS of product 5 (Supplementary Figure 23) generated from the thermal decomposition of TCNHPI.

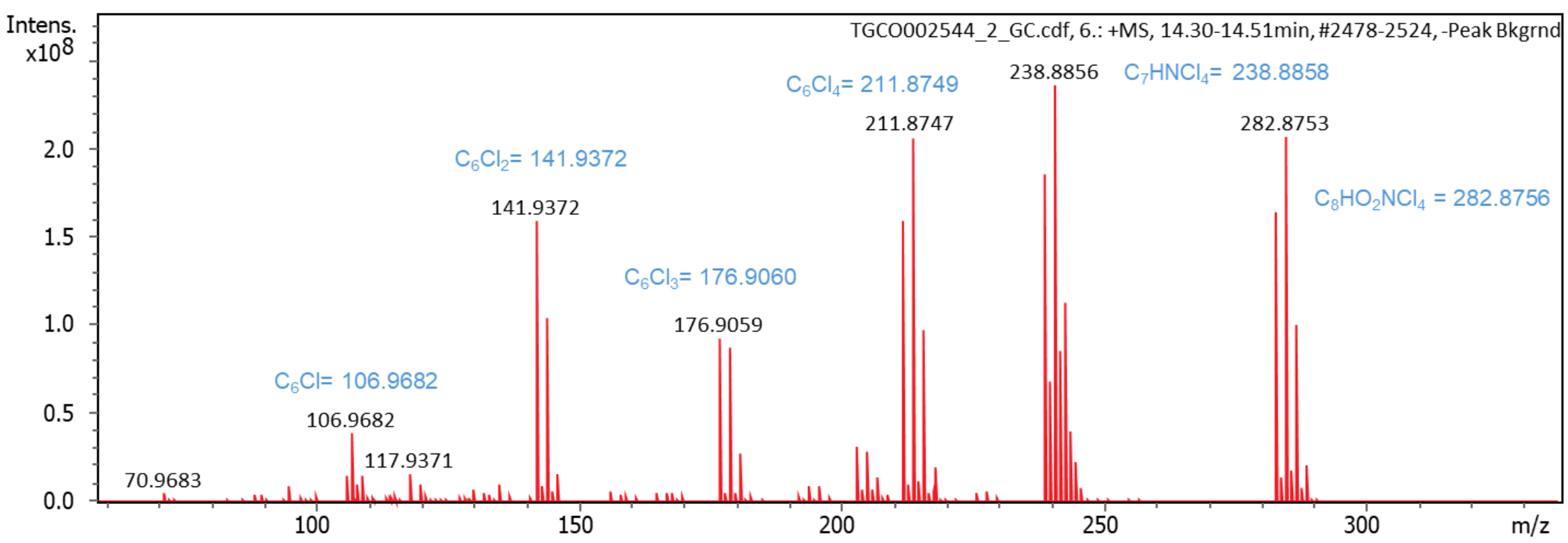

Supplementary Figure 26: MS of product 6 (Supplementary Figure 23) generated from the thermal decomposition of TCNHPI.

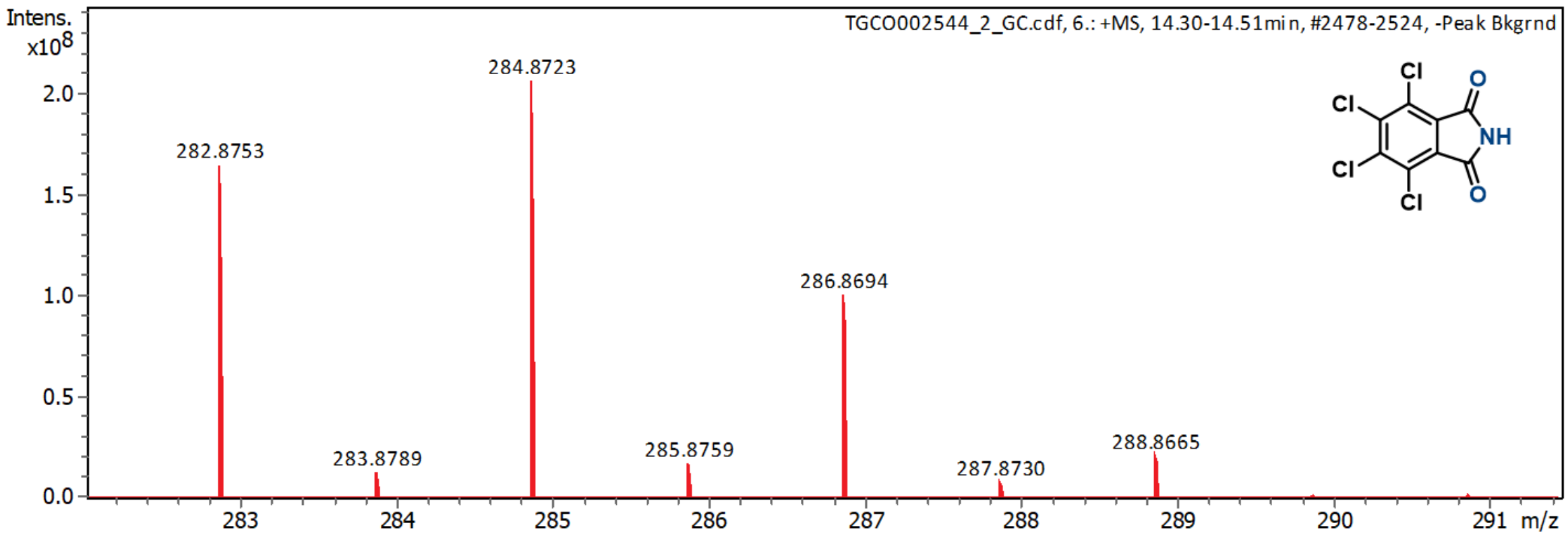

Supplementary Figure 27: Zoomed in MS of product 6 (Supplementary Figure 23) generated from the thermal decomposition of TCNHPI.

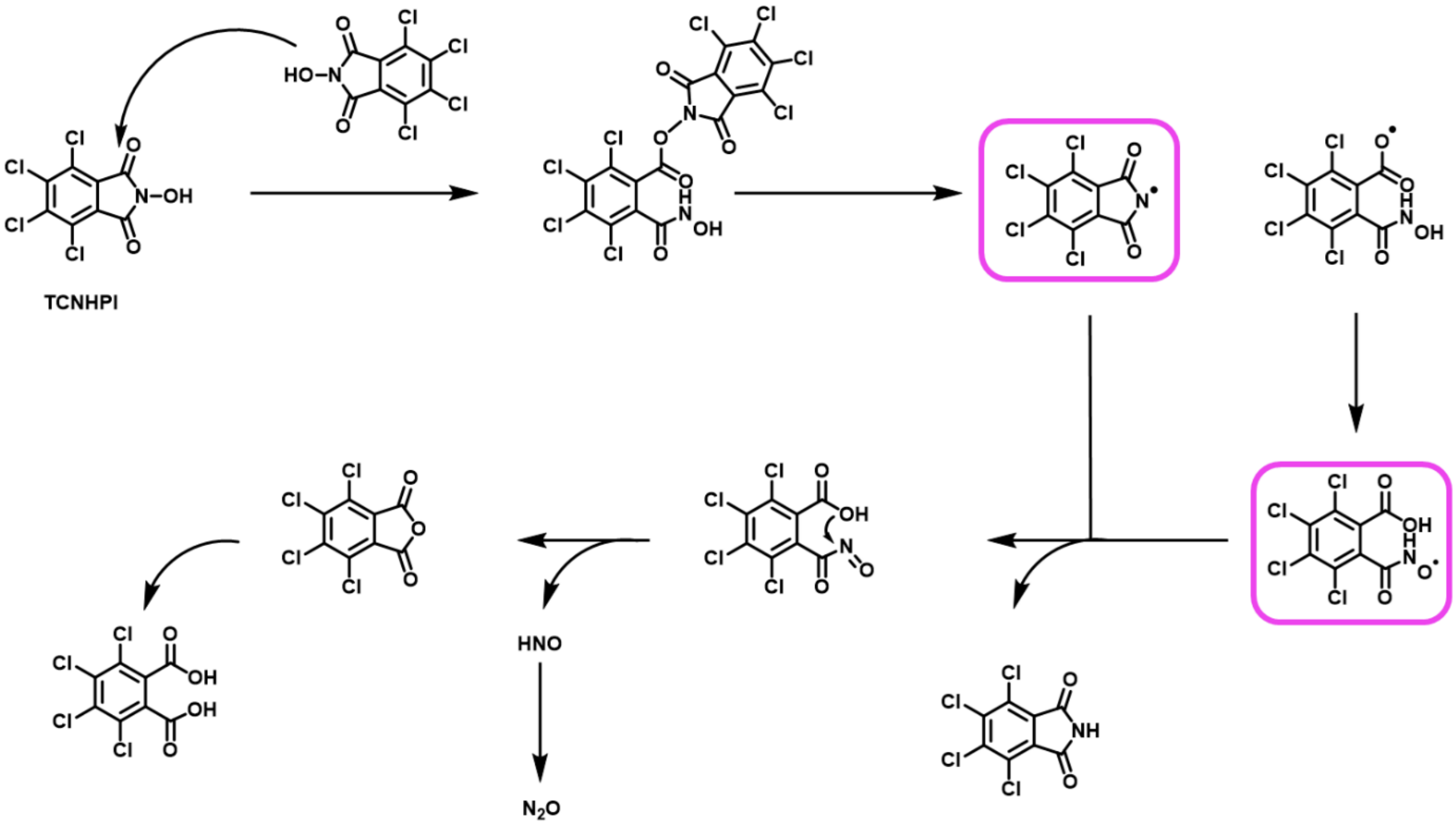

Supplementary Scheme 3: The potential pathway for the generation of radicals from TCNHPI. The open nitroxide structure, HTCPINO and the closed nitrogen radical, TCPI, are highlighted in a purple box. This is based on a previous literature report published for the thermal degradation of NHPI.[9]

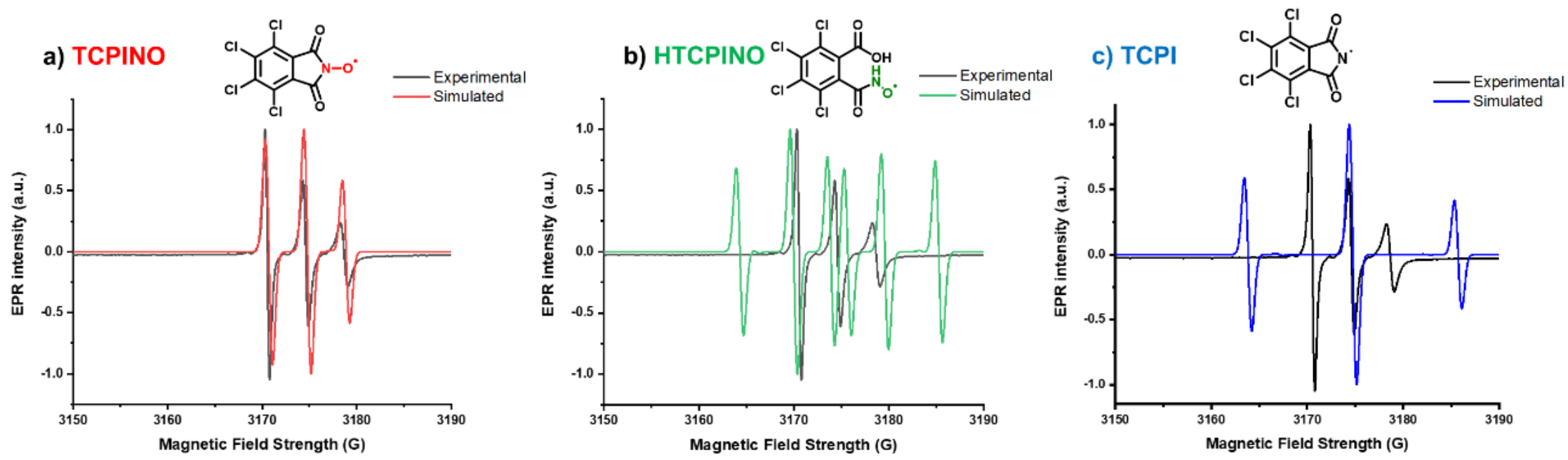

Supplementary Figure 28: EPR spectra obtained on heating TCNHPI to 230 °C. Experimental data is shown in black and simulated spectra for the closed TCPINO, open HTCPINO are shown in red, green and blue, respectively. Experimental (TCNHPI): a_iso(^{14}N)=4.0 G; Simulatin: TCPINO: a_iso(^{14}N)=4.1 G; HTCPINO: a_iso(^{14}N)=5.2 G; a_iso(^{1}H)=9.6 G and TCPI: a_iso(^{14}N)=11.0 G. EPR was performed in the absence of a spin trap, so even though the dominant species present is TCPINO, due to the short lifetimes of HHTCPINO and TCPI, this data does not preclude the formation of the other more reactive radical species.

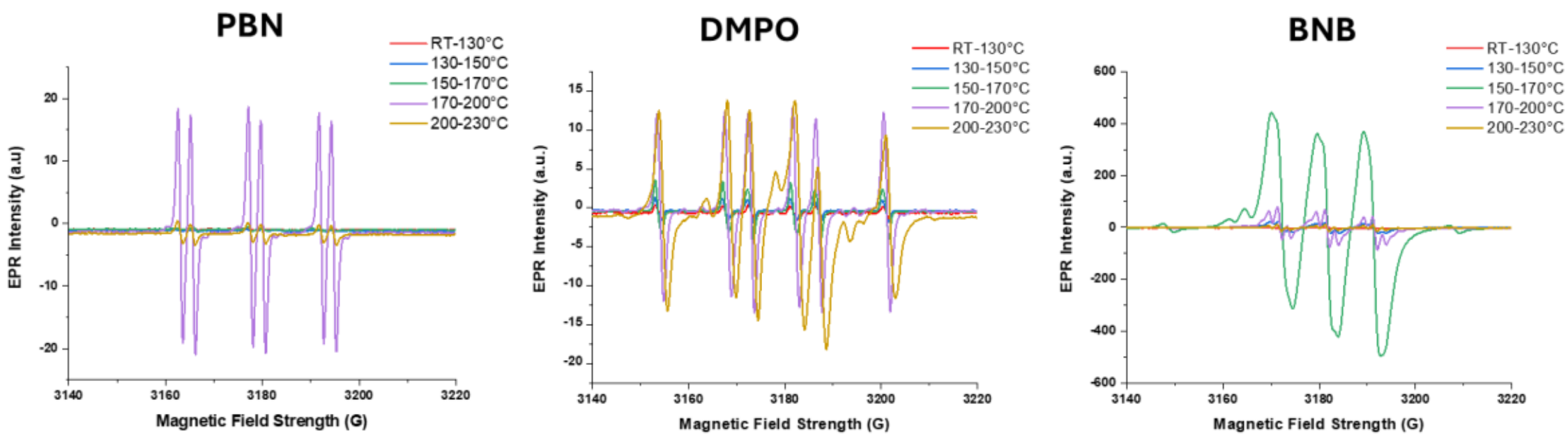

Supplementary Figure 29: EPR data on heating PBN, DMPO or BNB alone to 230°C. In all cases decomposition was observed prior to reaching the depolymerization temperatures. This prevents these spin traps from being used for the EPR measurements.

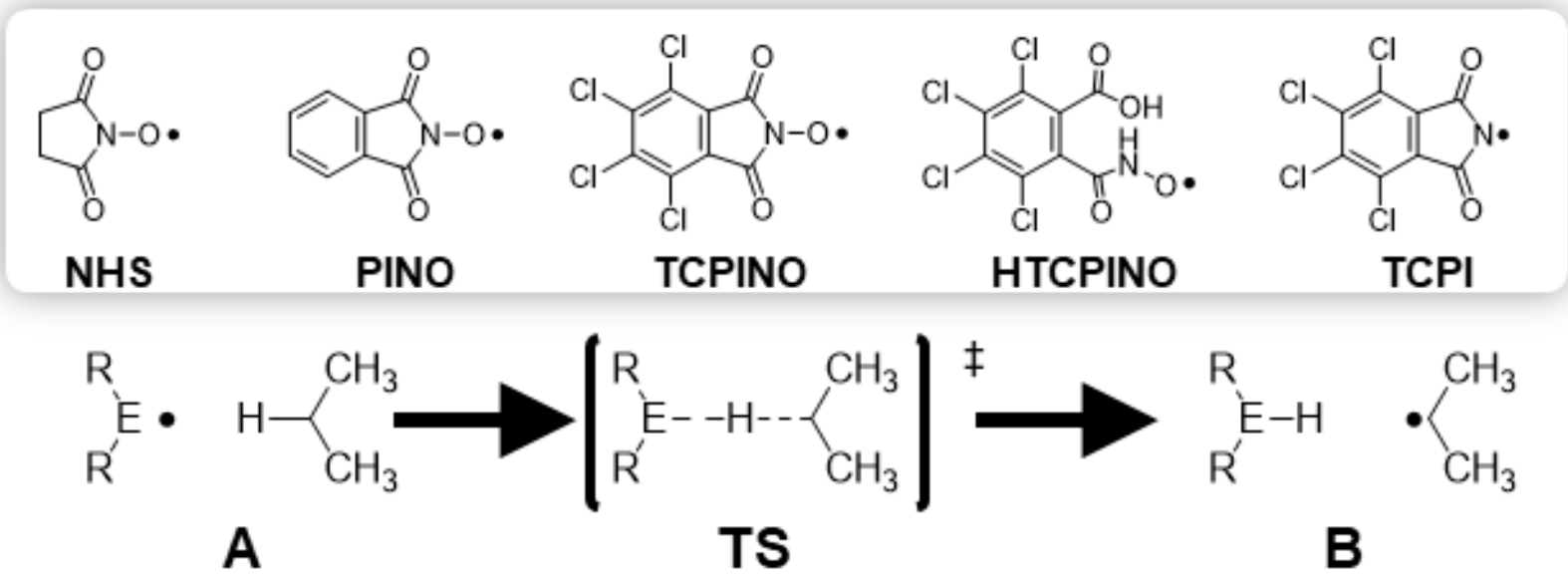

	A^a	TS^a	B^a	BDE (E–H)^b
**NHS**	0.0	22.8	10.9	85.2
**PINO**	0.0	24.6	13.7	82.5
**TCPINO**	0.0	23.0	12.5	83.6
**HTCPINO**	0.0	27.9	16.4	80.2
**TCPI**	0.0	8.2	–25.7	123.0

[a]Relative free energy in kcal mol^{-1}. [b]Bond dissociation energy in kcal mol^{-1}

Supplementary Figure 30: DFT calculated model hydrogen atom transfer reactions for various nitrogen and oxygen atom-centered radicals with propane.

Supplementary Table 9: Cartesian coordinate of DFT models used for propane model HAT simulations.

**Propane (–119.0733101 Hartree)**				**Propane_radical (–118.4065841 Hartree)**			
C	1.266800	-0.260678	0.000000	C	-1.290656	-0.199973	0.005394
H	1.301080	-0.906720	-0.882934	H	-1.483359	-0.599121	1.014270
H	1.301082	-0.906718	0.882935	H	-1.285396	-1.063148	-0.670920
H	2.169055	0.356406	0.000000	H	-2.141628	0.432975	-0.257858
C	0.000000	0.590992	0.000000	C	0.000000	0.541894	-0.063186
H	0.000000	1.248126	0.876842	H	0.000000	1.606905	0.143408
H	0.000000	1.248126	-0.876842	C	1.290656	-0.199973	0.005394
C	-1.266800	-0.260678	0.000000	H	1.285396	-1.063148	-0.670919
H	-1.301081	-0.906720	0.882934	H	2.141628	0.432974	-0.257859
H	-2.169055	0.356406	0.000001	H	1.483360	-0.599120	1.014270
H	-1.301081	-0.906719	-0.882935	**TS_HTCPINO (–2621.0849939 Hartree)**			
**TS_PINO (–706.4336621 Hartree)**				C	-0.54837	-1.384812	-0.496369
C	C	0.412225	0.121948	C	0.384787	-0.472978	-0.821751
C	C	0.089977	-0.227489	C	0.135903	0.896949	-0.722686

C	C	-1.15108	-0.122382
C	C	-2.10001	0.335990
C	C	-1.80472	0.688725
C	O	2.200299	0.583531
H	C	1.776968	0.037350
H	O	2.13412	0.414948
H	O	2.196535	1.051932
H	H	2.467428	0.866695
C	C	2.810716	-0.556501
O	H	2.989925	-0.553628
N	C	1.598087	-0.965579
C	H	0.685803	-0.733276
O	H	1.716896	-0.892356
O	H	1.474287	-1.414780
H	C	4.04751	-0.458689
C	H	4.427028	0.706443
H	H	4.848683	0.620015
C	H	3.821299	0.649451
H	Cl	-1.49267	-0.162064
H	Cl	-3.62844	1.590961
H	Cl	-2.98876	0.498538
C	Cl	-0.21173	1.740274
H	N	2.595326	2.747155
H	H	3.508139	1.594636
H	C	1.136831	1.723425
**TS_TCPINO (–2544.6815666 Hartree)**			
C	H	1.481958	-0.200378
C	-0.131697	0.591606	-0.445230
C	0.012660	-0.794585	-0.406278
C	1.228726	-1.386383	-0.121468
C	2.328498	-0.543056	0.129037
C	2.183103	0.852761	0.089939
C	-1.317001	-1.425619	-0.701398

C	-1.075842	1.357352	-0.208941
C	-2.069363	0.445422	0.172934
C	-1.848183	-0.931816	0.006457
O	2.268616	1.968355	-0.537631
C	1.72832	-0.905046	-1.367802
O	2.083431	-0.626631	-2.499292
O	2.150684	-1.966117	0.735756
H	2.442086	-1.157624	1.526849
C	2.773548	-0.233498	2.505311
H	2.916923	-0.948089	3.319961
C	1.556717	0.642087	2.652332
H	0.648898	0.055904	2.817848
H	1.675318	1.310704	3.517517
H	1.419078	1.271776	1.772086
C	4.037586	0.405222	1.995524
H	4.39978	1.153573	2.716499
H	4.837791	-0.326856	1.858902
H	3.861428	0.927807	1.052375
Cl	-1.31675	3.070327	0.013513
Cl	-3.567296	1.015556	0.84052
Cl	-3.090361	-2.06844	0.4302
Cl	-0.35503	-3.082618	-0.767117
N	2.542609	-1.561402	-0.493251
H	3.433256	-1.908834	-0.831654
C	1.223788	1.857618	-1.128313
O	0.752749	2.556995	-2.220072
H	1.636993	3.139598	-2.438864
**TS_NHS (–554.0636048 Hartree)**			
C	-0.70007	1.281683	-0.15995
O	0.073249	2.189604	-0.31849
N	-0.54993	0.004058	-0.7065
C	-1.44542	-0.95473	-0.21636
O	-1.38118	-2.13183	-0.44789

O	-1.647134	-2.578573	-0.694758	O	0.460884	-0.31112	-1.50875
N	-2.158494	-0.334702	-0.975652	H	1.388045	-0.58424	-0.784
C	-1.561493	0.916986	-0.764922	C	2.309513	-0.7207	0.147013
O	-2.123933	1.976691	-0.810410	H	2.789191	-1.64717	-0.17994
O	-3.444509	-0.475697	-1.268646	C	1.535855	-0.86261	1.43324
H	-4.033174	-0.462415	-0.218533	H	0.818888	-1.68753	1.386731
C	-4.510260	-0.321057	1.007922	H	2.223086	-1.06521	2.266041
H	-5.278515	-1.098912	1.009409	H	1.006738	0.065584	1.67834
C	-5.037880	1.090295	1.031260	C	3.155858	0.518092	-0.00006
H	-5.843515	1.230592	0.307801	H	2.543894	1.416717	0.117228
H	-5.434785	1.328912	2.027402	H	3.941733	0.534502	0.767366
H	-4.243022	1.806080	0.803402	H	3.64159	0.558422	-0.9772
C	-3.337175	-0.624476	1.904228	C	-2.44409	-0.20924	0.653879
H	-3.653634	-0.607765	2.956108	H	-3.44338	-0.34489	0.23549
H	-2.914050	-1.612384	1.701748	H	-2.43984	-0.66677	1.645455
H	-2.551319	0.132589	1.800330	C	-1.9771	1.255108	0.662896
Cl	1.390771	-3.096161	-0.075714	H	-2.69661	1.937973	0.205514
Cl	3.861128	-1.234997	0.486064	H	-1.75477	1.635379	1.661637
Cl	3.538178	1.864335	0.399650				
Cl	0.741283	3.142742	-0.251404				

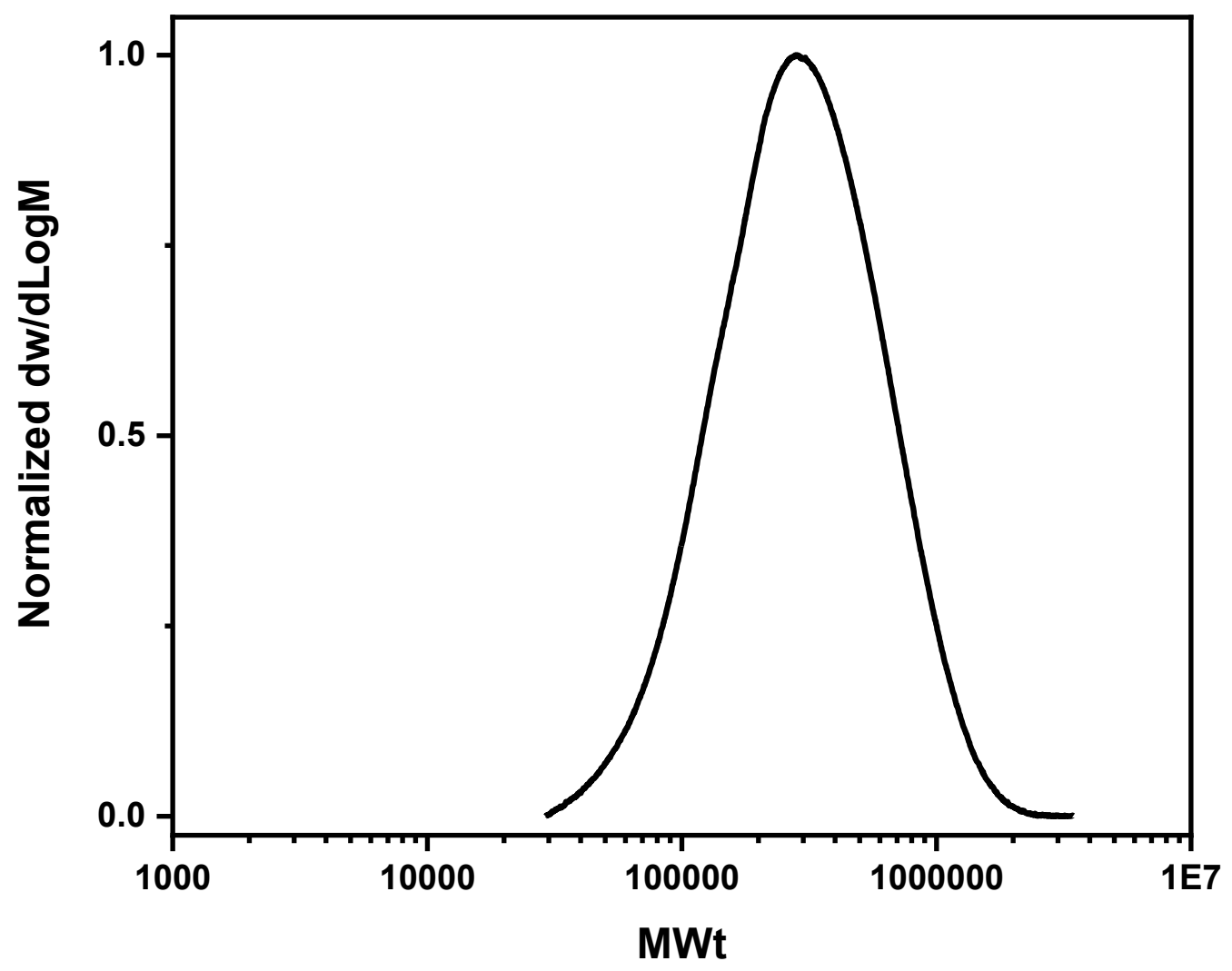

Supplementary Figure 31: SEC trace of poly(phenyl methacrylate) prepared by free radical polymerization. The number-averaged molecular weight (M_n) of the polymer was 214 kDa.

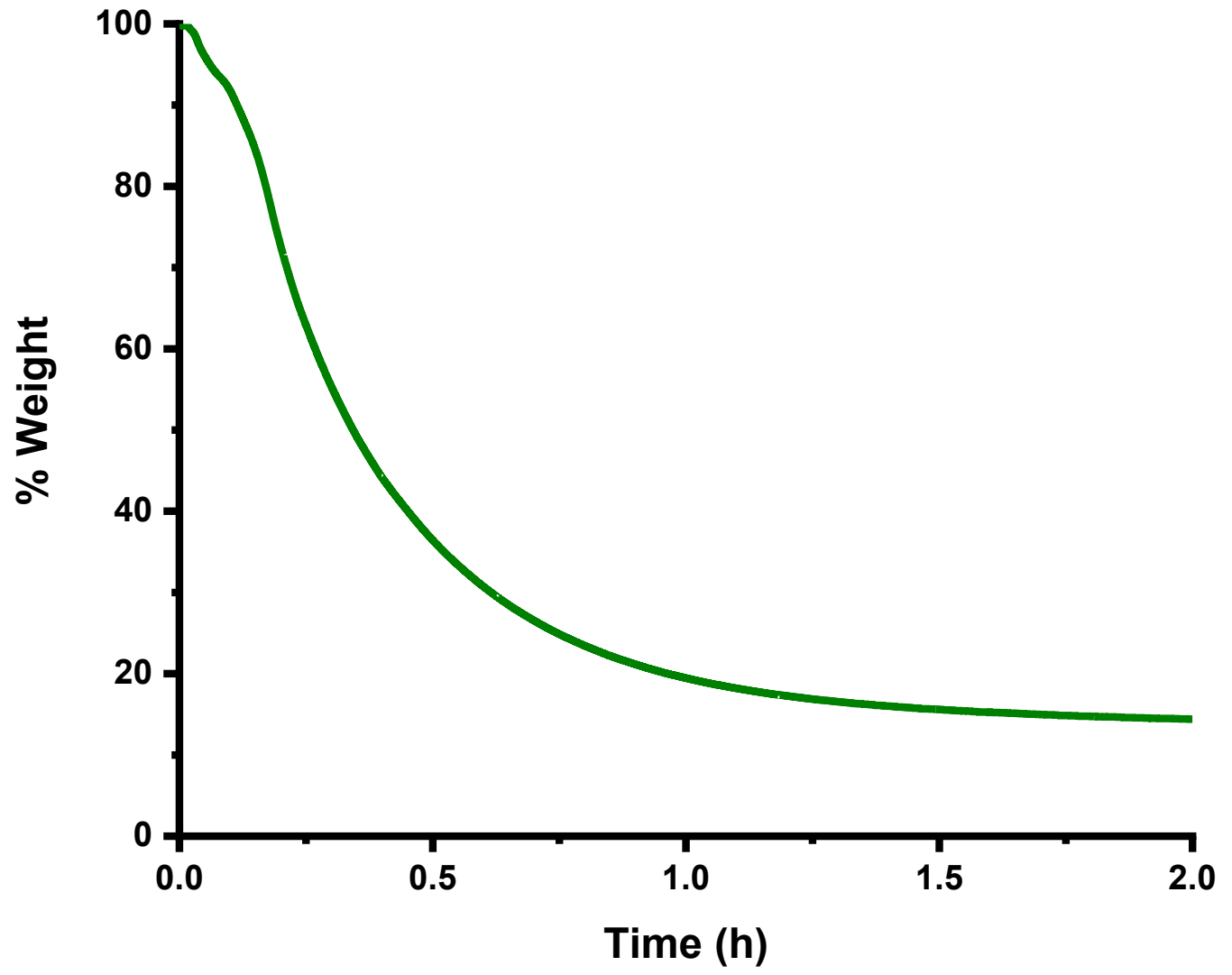

Supplementary Figure 32: TGA data for the depolymerization of poly(phenyl methacrylate). Isothermal reaction was performed for 2 hours at 230 °C with 0.05 equivalents of TCNHPI relative to the polymer repeat unit concentration. The final depolymerization was calculated to be 87%.

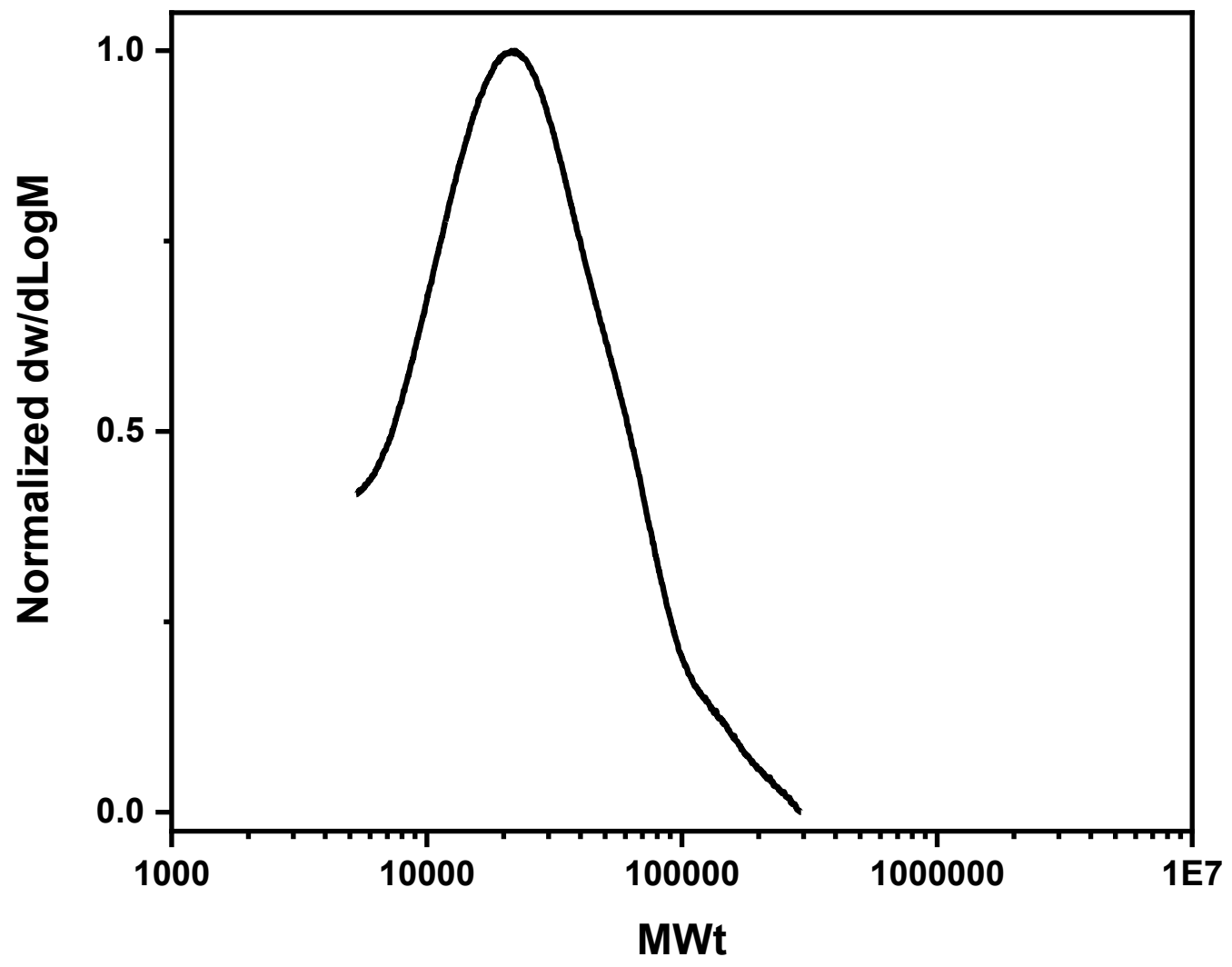

Supplementary Figure 33: SEC trace of the residual polymer after the depolymerization of poly(phenyl methacrylate). Isothermal reaction had been performed for 2 hours at 230 °C with 0.05 equivalents of TCNHPI relative to the polymer repeat unit concentration. The M_n of the polymer fragments was 17.4 kDa.

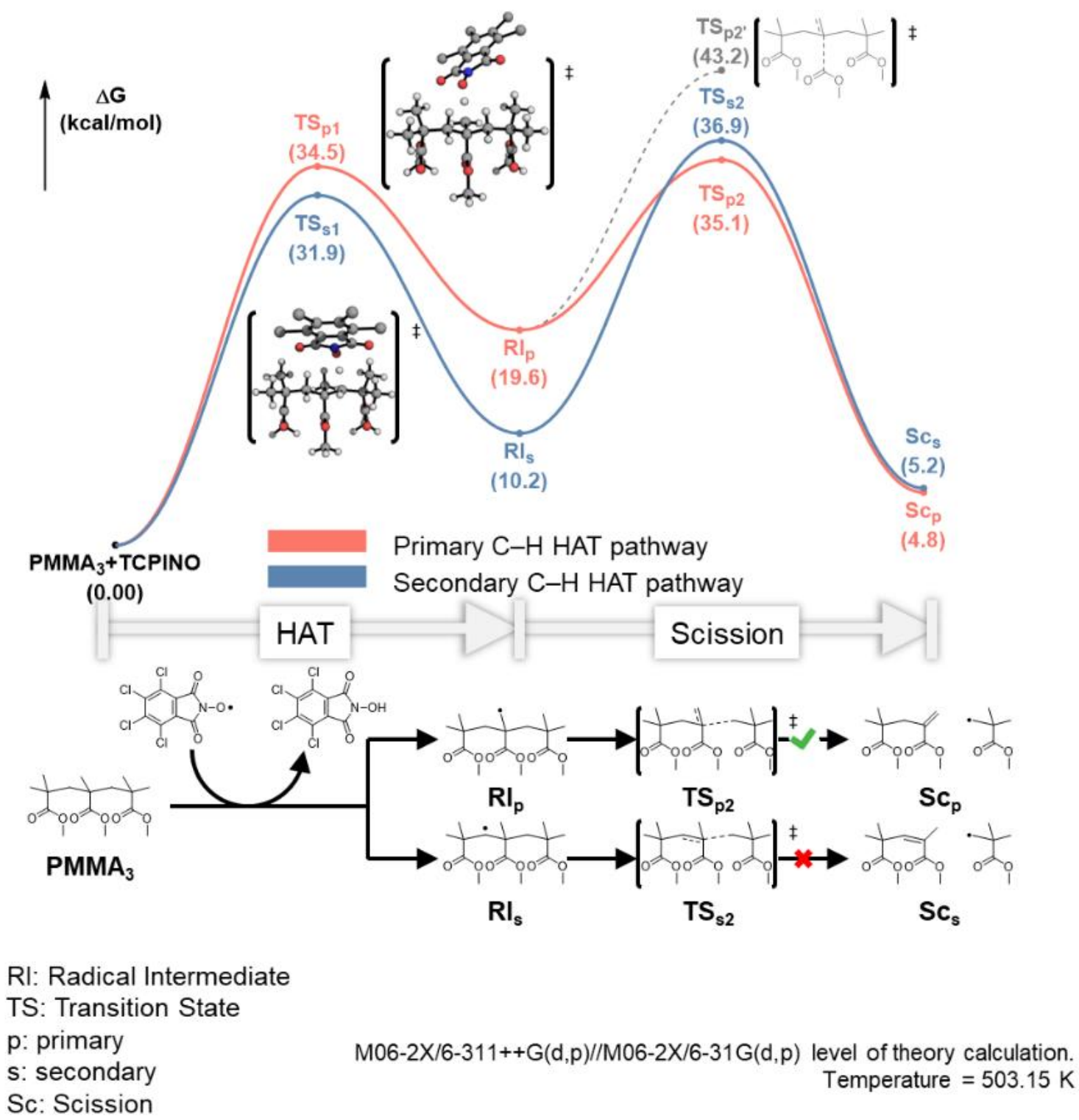

Supplementary Figure 34: DFT calculations for the reaction of TCPINO with the backbone of PMMA. This is expanded from Figure 4 and shows the reaction of TCPINO with a PMMA trimer.

Supplementary Table 10: Cartesian coordinates of DFT models used for the depolymerization simulation.

PMMA$_3$ (-1077.5239179 Hartree)				TS$_{S1}$ (-3503.1353833 Hartree)			
C	-2.683923	-1.176981	0.061322	C	4.077876	1.496753	-0.116519
C	-1.232380	-1.565087	0.469858	C	3.100477	0.606407	-0.519354
C	0.000002	-0.829816	-0.128488	C	3.303883	-0.772221	-0.466487
C	1.232352	-1.565087	0.469911	C	4.491467	-1.311314	-0.009515
C	2.683903	-1.176993	0.061401	C	5.501792	-0.420532	0.403012
C	-3.610112	-1.686127	1.178463	C	5.297157	0.967406	0.350456
C	0.000001	0.615336	0.377859	C	2.067759	-1.457932	-0.963229
O	0.000062	1.590959	-0.342720	O	1.814470	-2.626413	-1.077422
O	-0.000099	0.681917	1.706414	N	1.189679	-0.407678	-1.270663
C	-0.000104	2.004932	2.244108	C	1.723204	0.872992	-1.050344
C	0.000048	-0.800558	-1.656155	O	1.161299	1.913910	-1.256954
C	-2.818779	0.344813	0.037521	O	0.010029	-0.618599	-1.843031
O	-2.838984	1.033501	1.032173	Cl	4.724796	-3.011522	0.051710
O	-2.894591	0.851408	-1.199622	Cl	6.997051	-1.045069	0.974549
C	-2.938522	2.280402	-1.264317	Cl	6.542416	2.036225	0.859853
C	-3.114319	-1.823982	-1.260810	Cl	3.813131	3.192333	-0.183359
C	2.818760	0.344801	0.037581	C	-2.025111	-2.605468	-0.178808
O	2.838774	1.033522	1.032215	C	-1.706487	-1.103067	-0.034704
O	2.894764	0.851359	-1.199565	C	-2.705907	0.030681	-0.301465
C	2.938737	2.280352	-1.264307	C	-1.920396	1.350242	-0.057354
C	3.114311	-1.823997	-1.260724	C	-2.576449	2.736487	-0.307411
H	-1.124572	-2.632434	0.236725	C	-1.060106	-3.377669	0.733229
H	-1.158130	-1.467275	1.557506	C	-3.816720	-0.079154	0.757629
H	1.124540	-2.632438	0.236797	O	-4.995112	-0.172773	0.497041
H	1.158074	-1.467248	1.557555	O	-3.325223	-0.063681	1.992422
H	-4.660136	-1.525342	0.916628	C	-4.305124	-0.140654	3.030774
H	-3.455622	-2.759551	1.325022	C	-3.342961	-0.052989	-1.688947
H	-3.401958	-1.163012	2.113226	C	-3.442618	-2.866470	0.357789
H	0.898452	2.529734	1.913787	O	-3.712766	-2.938054	1.533778
H	-0.898585	2.529779	1.913655	O	-4.354349	-3.000074	-0.610648
H	-0.000185	1.883203	3.325474	C	-5.700123	-3.175819	-0.153261
H	0.875042	-0.265246	-2.026267	C	-1.861990	-3.112070	-1.622461
H	0.000022	-1.820314	-2.055281	C	-3.973860	2.747227	0.305249
H	-0.874880	-0.265178	-2.026322	O	-4.183871	2.725144	1.497609
H	-3.016540	2.523541	-2.322343	O	-4.956022	2.750760	-0.604866
H	-3.805244	2.653933	-0.715704	C	-6.278677	2.676351	-0.064333
H	-2.023368	2.692449	-0.836467	C	-2.564681	3.130086	-1.789141
H	-4.148273	-1.556544	-1.495503	H	-0.788418	-0.883194	-0.968676
H	-2.495255	-1.527495	-2.105578	H	-1.128110	-0.901757	0.873835
H	-3.061060	-2.912404	-1.157298	H	-1.030980	1.327094	-0.693538
H	3.805398	2.653881	-0.715595	H	-1.586169	1.326852	0.985179
H	3.016900	2.523447	-2.322332	H	-1.232208	-4.454035	0.644381
H	2.023538	2.692439	-0.836594	H	-0.028918	-3.175336	0.431494

H	2.495260	-1.527502	-2.105500
H	4.148270	-1.556563	-1.495402
H	3.061044	-2.912419	-1.157215
C	3.610070	-1.686143	1.178559
H	3.455581	-2.759570	1.325107
H	4.660098	-1.525353	0.916746
H	3.401896	-1.163036	2.113321
**TS_{P1} (-3503.1273188 Hartree)**			
C	4.486910	1.090046	-0.571712
C	3.285947	0.424840	-0.730522
C	3.098332	-0.866112	-0.237961
C	4.103586	-1.538981	0.430020
C	5.335986	-0.878836	0.600578
C	5.525210	0.421103	0.105759
C	1.702246	-1.304493	-0.565171
O	1.113700	-2.314273	-0.288454
N	1.159141	-0.221476	-1.267073
C	2.017783	0.881701	-1.393660
O	1.757536	1.921663	-1.930803
O	-0.076350	-0.248863	-1.765201
Cl	3.855476	-3.129652	1.029337
Cl	6.616630	-1.677781	1.422873
Cl	7.036374	1.209852	0.326733
Cl	4.704629	2.681639	-1.180251
C	-2.314524	2.880183	-0.238746
C	-2.830921	1.695603	-1.105733
C	-2.955647	0.261541	-0.516732
C	-3.383557	-0.636316	-1.713331
C	-3.508606	-2.179673	-1.558117
C	-2.832844	4.169410	-0.898187
C	-4.067586	0.248781	0.545504
O	-3.894884	-0.087287	1.697623
O	-5.231905	0.649736	0.053414
C	-6.310234	0.657603	0.993223
C	-1.696466	-0.216860	0.168515
C	-2.947562	2.808402	1.149207
O	-4.109624	3.059496	1.373319
O	-2.093085	2.411559	2.102556
C	-2.669367	2.265415	3.405040
C	-0.782982	2.943782	-0.193772
C	-4.191433	-2.500232	-0.229861
O	-5.366861	-2.308690	-0.013768
O	-3.354280	-2.992930	0.691682
C	-3.943438	-3.240702	1.972433
C	-2.161981	-2.896045	-1.725794
H	-2.161406	1.635383	-1.972519
H	-3.824958	1.959193	-1.479538

H	-1.209692	-3.090648	1.775671
H	-4.977378	0.716029	2.954740
H	-4.857255	-1.077836	2.937361
H	-3.746670	-0.112687	3.964134
H	-4.095896	0.728132	-1.799452
H	-2.574500	0.066905	-2.458800
H	-3.850180	-1.008944	-1.822438
H	-6.35928	-3.31004	H
H	-5.769790	-4.071240	0.467010
H	-6.006279	-2.300622	0.421704
H	-2.172785	-4.158124	-1.683156
H	-2.455054	-2.541444	-2.337074
H	-0.808276	-3.042773	-1.902854
H	-6.461106	3.523695	0.599360
H	-6.948275	2.706375	-0.921884
H	-6.395479	1.743051	0.488729
H	-3.158574	2.467128	-2.415489
H	-2.956647	4.143413	-1.913777
H	-1.530491	3.113761	-2.146780
C	-1.729585	3.757475	0.472197
H	-0.684424	3.675314	0.158184
H	-2.070585	4.777581	0.272238
H	-1.800911	3.571654	1.545469
**RI_P (-1076.8470712 Hartree)**			
C	-2.685933	-1.182771	0.006536
C	-1.232282	-1.589266	0.387611
C	-0.000091	-0.811916	-0.163471
C	1.232156	-1.589339	0.387448
C	2.685830	-1.182923	0.006328
C	-3.603694	-1.699867	1.126477
C	-0.000056	0.601428	0.453768
O	0.000158	1.624959	-0.194938
O	-0.000166	0.570985	1.783299
C	-0.000077	1.855293	2.411145
C	-0.000233	-0.658753	-1.654174
C	-2.808875	0.339767	0.003647
O	-2.842966	1.014006	1.007759
O	-2.854330	0.863665	-1.227390
C	-2.889314	2.294034	-1.273209
C	-3.132814	-1.813284	-1.318549
C	2.809026	0.339600	0.003817
O	2.843009	1.013591	1.008099
O	2.854685	0.863815	-1.227078
C	2.889801	2.294193	-1.272515
C	3.132560	-1.813222	-1.318911
H	-1.118110	-2.638121	0.087415
H	-1.152603	-1.556366	1.478499

H	-2.665619	-0.465280	-2.525214
H	-4.350298	-0.250763	-2.051183
H	-2.434079	5.052298	-0.390174
H	-2.510377	4.202691	-1.943054
H	-3.922638	4.207123	-0.856082
H	-6.465700	-0.354873	1.370395
H	-6.071742	1.342843	1.808814
H	-7.180127	1.002846	0.438497
H	-1.697285	-1.259000	0.482543
H	-0.802732	-0.201970	-0.840216
H	-1.293460	0.453077	0.925867
H	-1.851701	1.959153	4.054895
H	-3.091238	3.216264	3.735820
H	-3.450534	1.504604	3.375687
H	-0.455370	3.807958	0.390734
H	-0.324099	2.058936	0.248021
H	-0.393541	3.045985	-1.209887
H	-4.760037	-3.958637	1.875673
H	-3.143526	-3.646402	2.588890
H	-4.321566	-2.305540	2.388099
H	-1.395933	-2.591976	-1.013995
H	-2.296187	-3.976807	-1.627427
H	-1.777760	-2.690472	-2.729758
C	-4.457201	-2.658995	-2.670024
H	-4.077934	-2.332125	-3.642915
H	-4.522132	-3.750929	-2.679343
H	-5.458747	-2.252526	-2.519547
**RIs (-1076.800955 Hartree)**			
C	C	-1.443094	0.131159
C	C	-0.436448	-0.375585
C	C	1.335289	-0.544145
C	C	2.698102	0.100427
C	C	-3.730162	-0.018954
C	C	0.055819	1.594939
C	O	0.796006	0.19834
O	O	-0.409432	-0.308816
O	C	0.015337	1.494341
C	C	-0.355602	2.276013
C	C	-3.094532	-2.036962
C	O	-3.518929	0.018887
O	O	-2.704656	0.913436
O	C	-2.777866	-1.235103
C	C	-3.487800	-1.424465
C	C	2.959282	-0.746508
C	O	2.574607	0.290634
O	O	3.708686	1.394046
O	C	3.870410	-0.783076

H	1.117913	-2.638170	0.087194
H	1.152562	-1.556522	1.478344
H	-4.655539	-1.535495	0.874131
H	-3.449194	-2.774593	1.262687
H	-3.387164	-1.184426	2.063543
H	0.897829	2.401303	2.115574
H	-0.897816	2.401499	2.115434
H	-0.000183	1.659735	3.481648
H	0.915449	-0.344765	-2.136703
H	-0.916139	-0.345261	-2.136603
H	-2.927237	2.552345	-2.329857
H	-3.775079	2.663192	-0.752623
H	-1.990176	2.695964	-0.804485
H	-4.144341	-1.488414	-1.576370
H	-2.478135	-1.569840	-2.153822
H	-3.144250	-2.901682	-1.203631
H	3.775515	2.663136	-0.751690
H	2.927914	2.552780	-2.329089
H	1.990622	2.696077	-0.803828
H	2.477843	-1.569571	-2.154092
H	4.144099	-1.488399	-1.576741
H	3.143906	-2.901643	-1.204196
C	3.603596	-1.700397	1.126086
H	3.448958	-2.775133	1.262058
H	4.655446	-1.536111	0.873706
H	3.387198	-1.185143	2.063285
**TSP2 (-1076.8117669 Hartree)**			
C	-2.937696	-1.18268	-0.00545
C	-1.37678	-1.46983	0.31528
C	-0.32227	-0.71789	-0.48927
C	1.378866	-1.48728	0.788226
C	2.796994	-1.25839	0.309741
C	-3.72994	-1.99112	0.994117
C	-0.03607	0.716953	-0.13565
O	0.500168	1.521616	-0.8747
O	-0.42664	1.015172	1.120537
C	-0.06918	2.327682	1.593822
C	-0.05501	-1.06955	-1.79764
C	-3.19382	0.303934	0.205827
O	-3.76269	0.775146	1.166009
O	-2.73767	1.048941	-0.82891
C	-2.89845	2.472071	-0.71878
C	-3.24376	-1.59584	-1.44527
C	3.037064	0.256075	0.245104
O	2.881457	0.990316	1.202602
O	3.448095	0.678761	-0.95816
C	3.609232	2.106166	-1.08887

C	C	2.865921	-0.543525	C	3.088279	-1.98107	-1.01344
C	H	-1.396769	-1.372422	H	-1.23265	-2.54339	0.148593
H	H	-1.169380	-0.679619	H	-1.22677	-1.28674	1.380837
H	H	1.062749	-0.327668	H	1.057176	-2.52589	0.76253
H	H	1.074841	1.163633	H	1.095947	-0.94064	1.682947
H	H	-4.794434	1.939458	H	-4.79715	-1.84023	0.809556
H	H	-3.607739	1.673482	H	-3.51051	-3.05895	0.893806
H	H	-3.388873	2.237066	H	-3.52624	-1.68139	2.021413
H	H	1.024902	2.220871	H	1.015933	2.394641	1.690285
H	H	-0.000200	1.907575	H	-0.42461	3.095268	0.903146
H	H	-0.698422	3.293012	H	-0.55795	2.427378	2.562534
H	H	-0.778910	-2.177698	H	-0.32965	-2.04601	-2.17954
H	H	0.169241	-2.5697	H	0.502038	-0.39754	-2.43877
H	H	-2.235489	-2.453036	H	-2.14738	2.903898	-1.38001
H	H	-3.820092	-2.484059	H	-3.90452	2.760741	-1.03678
H	H	-2.323932	-1.141095	H	-2.74504	2.797321	0.311402
H	H	-4.549155	-0.821951	H	-4.30601	-1.41826	-1.64226
H	H	-2.957702	-0.407194	H	-2.66286	-1.03913	-2.18083
H	H	-3.392139	-1.793034	H	-3.05076	-2.6655	-1.5819
H	H	4.308725	-0.667024	H	4.324193	2.477972	-0.35077
H	H	4.528232	0.209335	H	3.981147	2.264172	-2.10093
H	H	2.893859	-1.499731	H	2.642862	2.592265	-0.94896
H	H	2.299117	-0.201746	H	2.460791	-1.60397	-1.82078
H	H	3.916722	-2.211805	H	4.132816	-1.84915	-1.30669
H	H	2.511649	-1.387463	H	2.895618	-3.05257	-0.89553
H	C	3.750981	-1.520092	C	3.753413	-1.79752	1.415181
C	H	3.622596	1.089176	H	3.580459	-2.86663	1.570522
H	H	4.761873	0.985814	H	4.798634	-1.66041	1.114251
H	H	3.640828	1.019167	H	3.589848	-1.26708	2.355344
H	3.069951	-1.599521	2.072813	**TS$_{S2}$ (-1076.8076101 Hartree)**			
**TS$_{P2}$' -1076.7991302)**				C	-2.721264	0.300398	0.950327
C	C	-1.372053	0.963675	0.735693	-1.37795	0.963675	0.735693
C	C	-0.363520	0.536835	-0.09576	-0.35189	0.536835	-0.09576
C	C	0.931725	-0.59249	1.365639	0.965106	-0.59249	1.365639
C	C	2.100446	-1.45045	0.892006	2.12755	-1.45045	0.892006
C	C	-3.647778	1.300514	1.687666	-3.65201	1.300514	1.687666
C	C	0.729802	1.541509	-0.42157	0.715817	1.541509	-0.42157
C	O	1.303653	1.588879	-1.50055	1.277452	1.588879	-1.50055
O	O	1.005986	2.389355	0.589869	0.992798	2.389355	0.589869
O	C	2.070083	3.308518	0.335498	2.072834	3.308518	0.335498
C	C	-0.587031	-0.46858	-1.20865	-0.57042	-0.46858	-1.20865
C	C	-3.368303	0.049444	-0.41175	-3.39769	0.049444	-0.41175
C	O	-3.558816	0.918354	-1.2304	-3.60806	0.918354	-1.2304
O	O	-3.732968	-1.24129	-0.60111	-3.75096	-1.24129	-0.60111
O	C	-4.321052	-1.52183	-1.86912	-4.37039	-1.52183	-1.86912
C	C	-2.597207	-0.98747	1.791571	-2.59249	-0.98747	1.791571
C	C	2.951275	-0.59812	-0.04585	2.993745	-0.59812	-0.04585

C	O	3.529882	0.380815	0.324608	3.611907	0.380815	0.324608
O	O	2.992799	-1.03885	-1.3147	2.991756	-1.03885	-1.3147
O	C	3.761755	-0.23384	-2.25038	3.741774	-0.23384	-2.25038
C	C	1.649463	-2.76842	0.258376	1.654457	-2.76842	0.258376
C	H	-1.170101	1.826814	1.363313	-1.17936	1.826814	1.363313
H	H	0.125604	-1.15283	1.831709	0.160526	-1.15283	1.831709
H	H	1.207069	0.31132	1.896052	1.247422	0.31132	1.896052
H	H	-4.638361	0.862599	1.864218	-4.63949	0.862599	1.864218
H	H	-3.223931	1.562585	2.657218	-3.21686	1.562585	2.657218
H	H	-3.755080	2.2099	1.095213	-3.77509	2.2099	1.095213
H	H	2.986520	2.750292	0.164139	2.995138	2.750292	0.164139
H	H	1.817097	3.930801	-0.53492	1.850324	3.930801	-0.53492
H	H	2.173172	3.919963	1.234524	2.150138	3.919963	1.234524
H	H	0.369641	-0.78125	-1.62979	0.384053	-0.78125	-1.62979
H	H	-1.130352	-1.34523	-0.85659	-1.11648	-1.34523	-0.85659
H	H	-1.168270	-0.00303	-2.01324	-1.14958	-0.00303	-2.01324
H	H	-4.574627	-2.58548	-1.8524	-4.60701	-2.58548	-1.8524
H	H	-5.212182	-0.92418	-1.99544	-5.27642	-0.92418	-1.99544
H	H	-3.608163	-1.29236	-2.68704	-3.68228	-1.29236	-2.68704
H	H	-3.583388	-1.4275	1.985366	-3.57455	-1.4275	1.985366
H	H	-1.993966	-1.73887	1.284118	-1.98504	-1.73887	1.284118
H	H	-2.126493	-0.74904	2.750463	-2.12266	-0.74904	2.750463
H	H	4.801514	-0.17058	-1.93916	4.787408	-0.17058	-1.93916
H	H	3.684200	-0.74812	-3.20708	3.654224	-0.74812	-3.20708
H	H	3.344969	0.764428	-2.30277	3.307098	0.764428	-2.30277
H	H	1.028775	-2.60326	-0.61918	1.031299	-2.60326	-0.61918
H	H	2.512573	-3.38041	-0.04735	2.50728	-3.38041	-0.04735
H	H	1.072240	-3.3351	0.993346	1.072917	-3.3351	0.993346
H	C	2.977713	-1.76771	2.137285	3.004015	-1.76771	2.137285
C	H	2.387493	-2.2933	2.88959	2.408352	-2.2933	2.88959
H	H	3.830096	-2.41064	1.857873	3.847043	-2.41064	1.857873
H	H	3.355345	-0.84761	2.57019	3.400295	-0.84761	2.57019
H	3.526655	-0.751515	2.181821	**Scs (-691.3202241 Hartree)**			
**ScP (-691.3215634 Hartree)**				C	-1.237468	0.990621	-0.269386
C	C	0.051636	1.665868	0.161545	0.063329	1.65985	0.170618
C	C	1.316856	1.232188	0.220143	1.332307	1.219693	0.233439
C	C	-1.436745	1.148988	-1.781950	-1.39371	1.16377	-1.8087
C	C	1.749852	-0.150932	-0.134592	1.776037	-0.15922	-0.1213
C	O	2.756572	-0.670766	0.283773	2.782741	-0.68281	0.313325
O	O	0.917748	-0.753089	-1.001569	0.951158	-0.76514	-1.00627
O	C	1.209238	-2.113851	-1.316833	1.236848	-2.13683	-1.32411
C	C	2.441320	2.091253	0.731402	2.450392	2.082809	0.7659
C	C	-1.344394	-0.485449	0.099928	-1.36782	-0.48158	0.084921
C	O	-1.915302	-1.320029	-0.558651	-1.92952	-1.31727	-0.59018
O	O	-0.828971	-0.739440	1.315539	-0.89316	-0.73135	1.326343
O	C	-0.977767	-2.086937	1.758087	-1.05966	-2.08253	1.785338
C	C	-2.405636	1.670209	0.478954	-2.4127	1.701448	0.440542

C	H	-0.100071	2.703287	0.463975	-0.08576	2.69285	0.485593
H	H	-2.389323	0.705183	-2.079426	-2.33811	0.720577	-2.1338
H	H	-1.439651	2.210755	-2.044027	-1.38944	2.225807	-2.07232
H	H	-0.632283	0.650860	-2.324466	-0.57715	0.665722	-2.33233
H	H	2.036025	-2.163885	-2.028917	2.077735	-2.19499	-2.02059
H	H	1.487866	-2.661278	-0.414528	1.487935	-2.69636	-0.42021
H	H	0.293876	-2.513111	-1.751592	0.324176	-2.51991	-1.77905
H	H	3.227078	2.196263	-0.022661	3.242255	2.206013	0.01844
H	H	2.080904	3.083670	1.006633	2.087113	3.071709	1.055254
H	H	2.903163	1.621042	1.603370	2.915291	1.604448	1.633309
H	H	-2.035049	-2.346849	1.843003	-0.6326	-2.10796	2.787541
H	H	-0.506171	-2.774373	1.052226	-2.1178	-2.35601	1.807299
H	H	-0.487500	-2.138664	2.728340	-0.53243	-2.77908	1.128334
H	H	-3.354708	1.205297	0.199000	-3.3637	1.253292	0.138617
H	H	-2.276170	1.586569	1.561063	-2.31275	1.613197	1.525856
H	H	-2.449715	2.730076	0.211486	-2.43785	2.763777	0.176956
H	-2.915301	1.707339	0.794832				

**Sc_{common} (-385.4907407 Hartree)**			
C	-1.190741	0.736278	1.403615
C	-1.001269	0.125704	0.047016
C	0.441786	-0.388443	-0.026953
O	0.770944	-1.545942	-0.104609
O	1.330157	0.621859	0.029421
C	2.697540	0.216853	-0.002139
C	-1.212778	1.185821	-1.046899
H	-0.793930	1.722439	1.611356
H	-1.498250	0.115433	2.236760
H	2.913993	-0.326000	-0.924549
H	3.282331	1.133480	0.050171
H	2.922287	-0.432727	0.846399
H	-0.540889	2.034058	-0.902587
H	-1.029738	0.761973	-2.038954
H	-2.245168	1.543625	-1.008838
C	-1.946933	-1.059354	-0.151944
H	-2.982578	-0.716507	-0.082235
H	-1.789375	-1.519980	-1.130094
H	-1.773121	-1.824280	0.607904

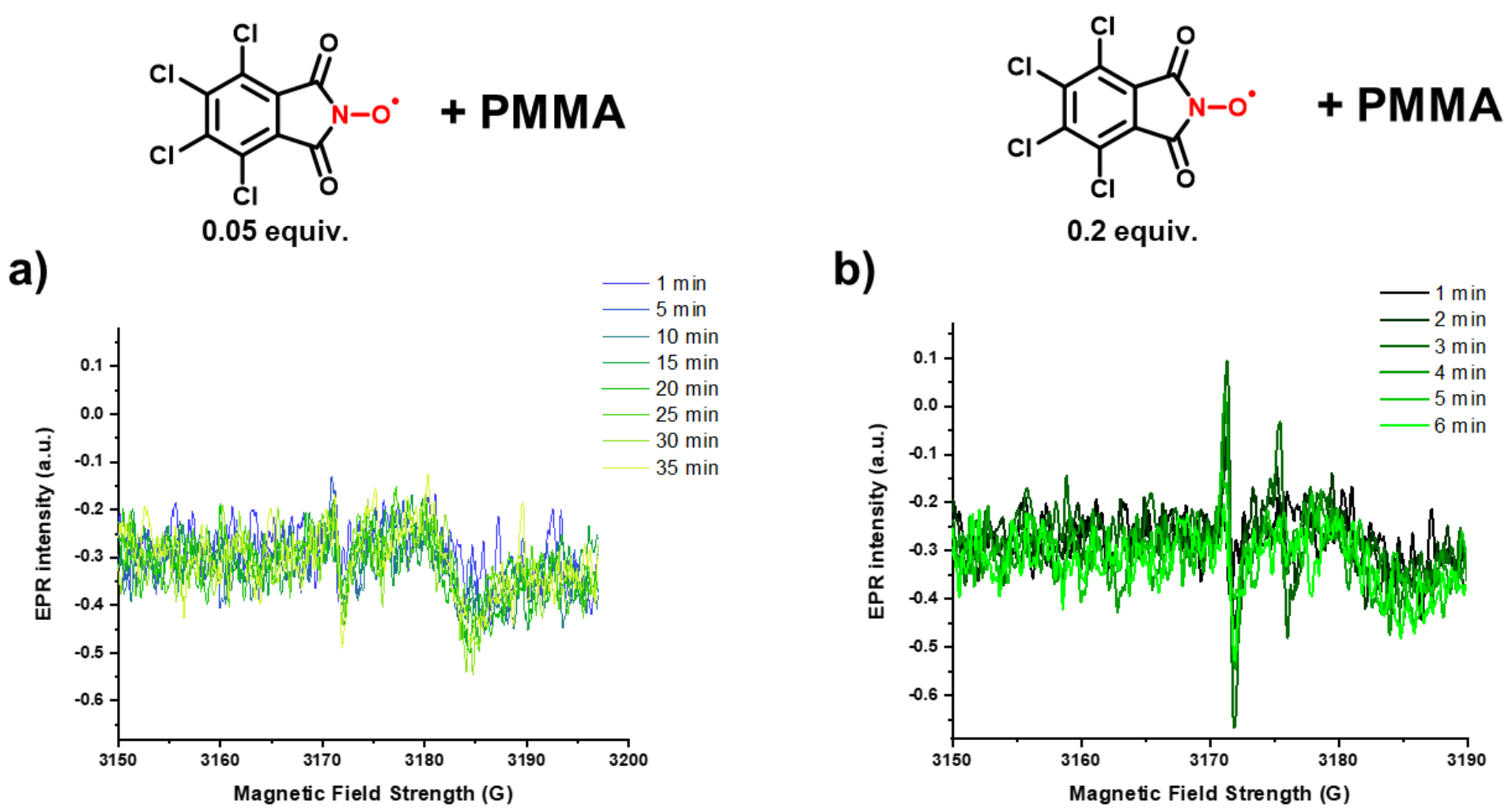

Supplementary Figure 35: EPR spectra for the reaction of TCNHPI and PMMA at 230 °C. In a), the reaction was performed with 0.05 equivalents of TCNHPI and in b), the reaction was performed with 0.20 equivalents of TCNHPI. In b, the signal of the TCPINO is shown in the early stages of the reaction, suggesting that this radical is being formed and reacting with the polymer.

Expanding the Scope of Mid-Chain Bulk Depolymerization

Supplementary Table 11: The polymers prepared by various methods and the results of their depolymerization. Reactions were performed with 0.05 equivalents of TCNHPI with respect to polymer repeat unit at 230 °C.

Polymerization Method	End-Group	M_n	*Đ*	Depolymerization %
ATRP	Br	129000	1.23	87
RAFT	DTB	182000	1.22	90
	TTC	90000	1.18	92
FRP	H + $=CH_2$ + $C(CH_3)_2CN$	138000	2.44	91

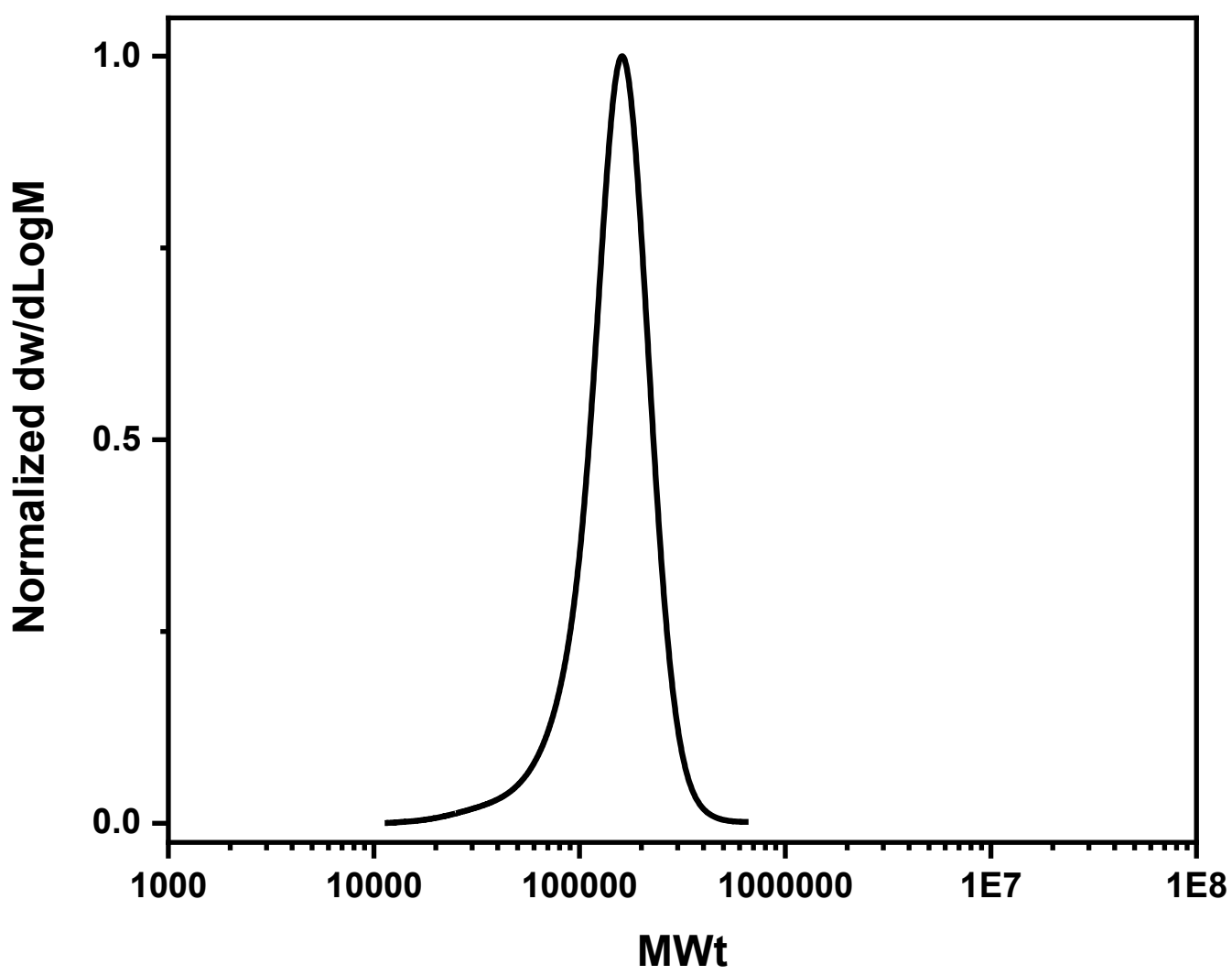

Supplementary Figure 36: SEC trace of PMMA-Br obtained by ATRP. The M_n was 129 kDa and the dispersity was 1.23.

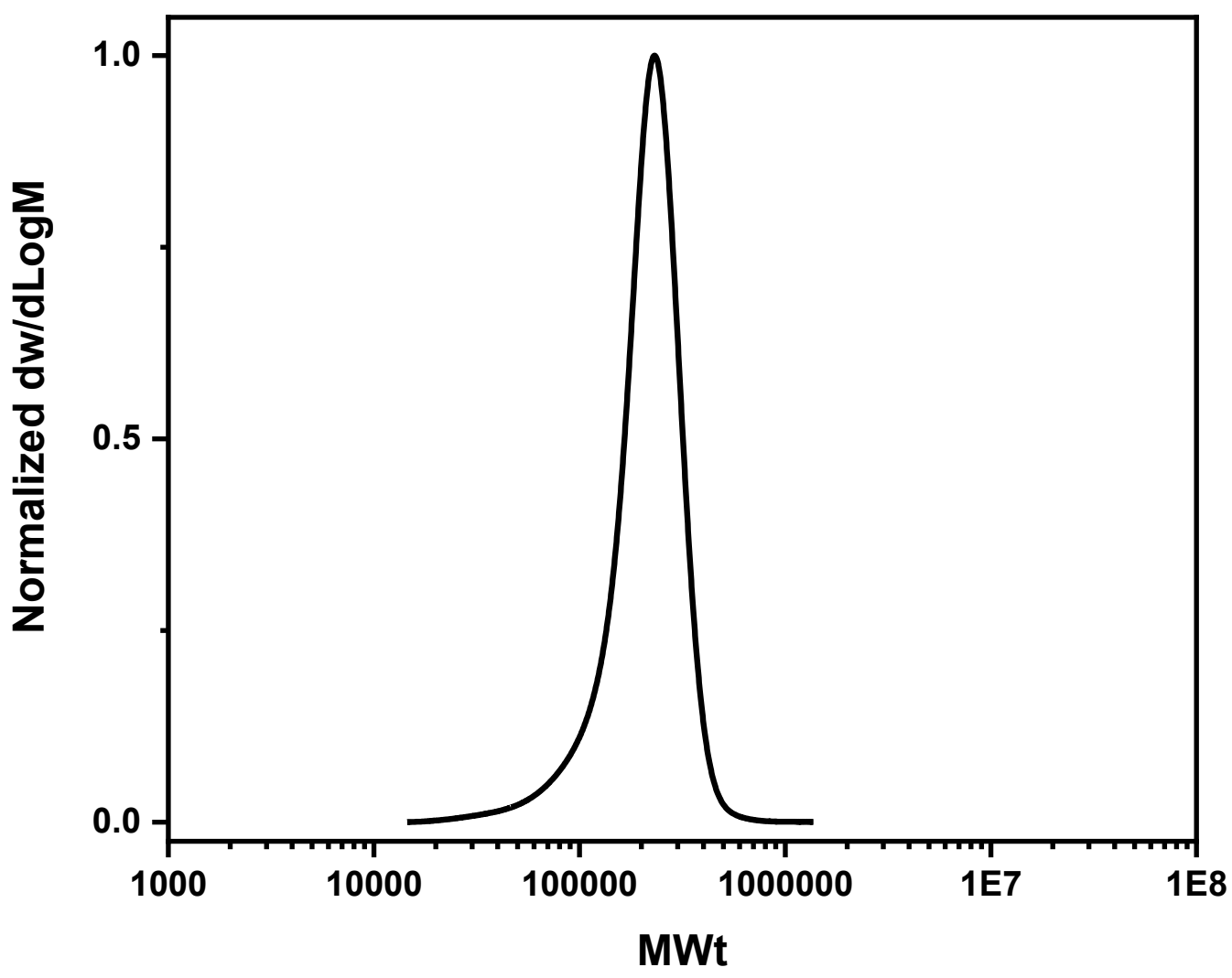

Supplementary Figure 37: SEC trace of PMMA obtained by RAFT using a dithiobenzaote CTA. The M_n was 182 kDa and the dispersity was 1.22.

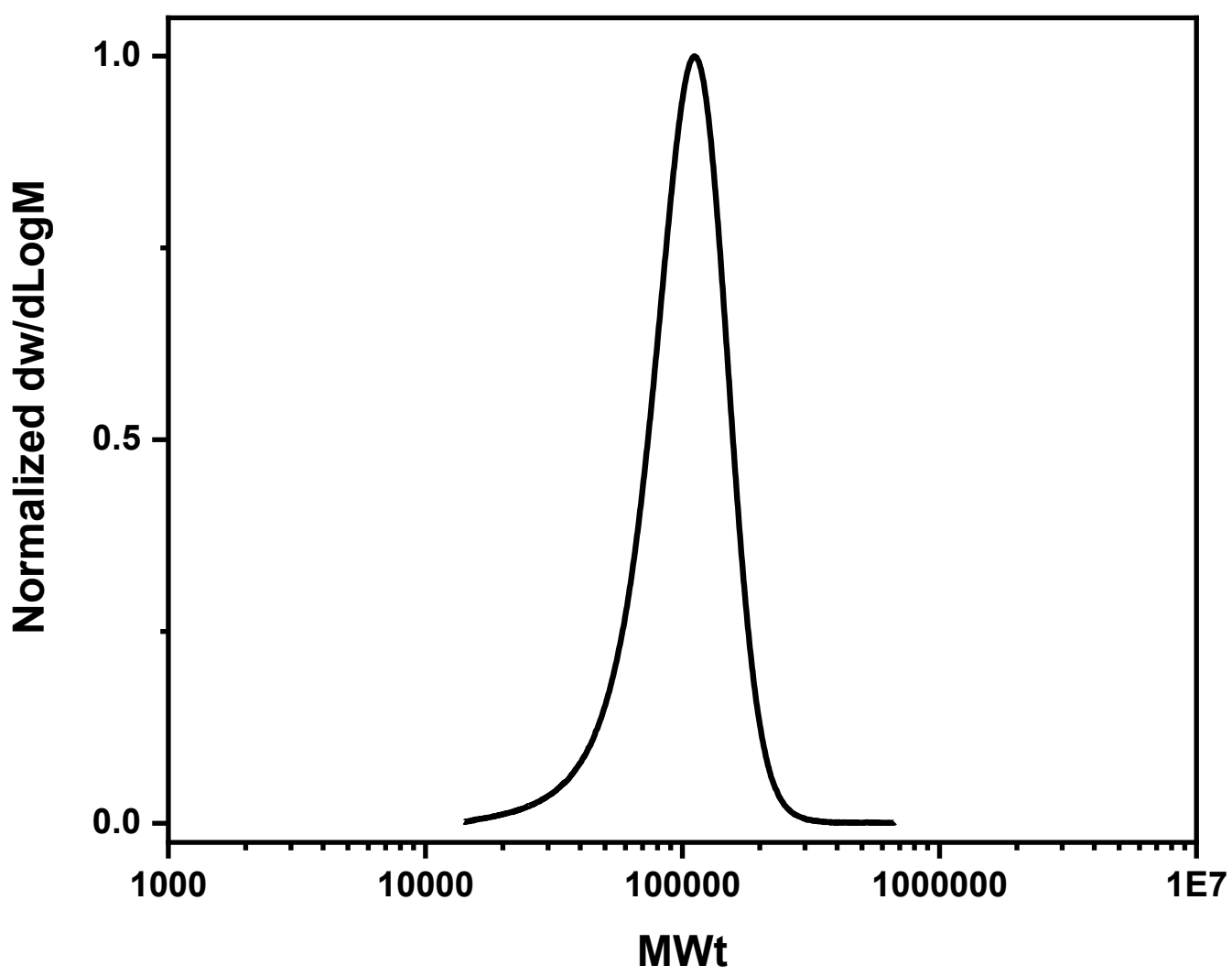

Supplementary Figure 38: SEC trace of PMMA obtained by RAFT using a trithiocarbonate CTA. The M_n was 90 kDa and the dispersity was 1.18.

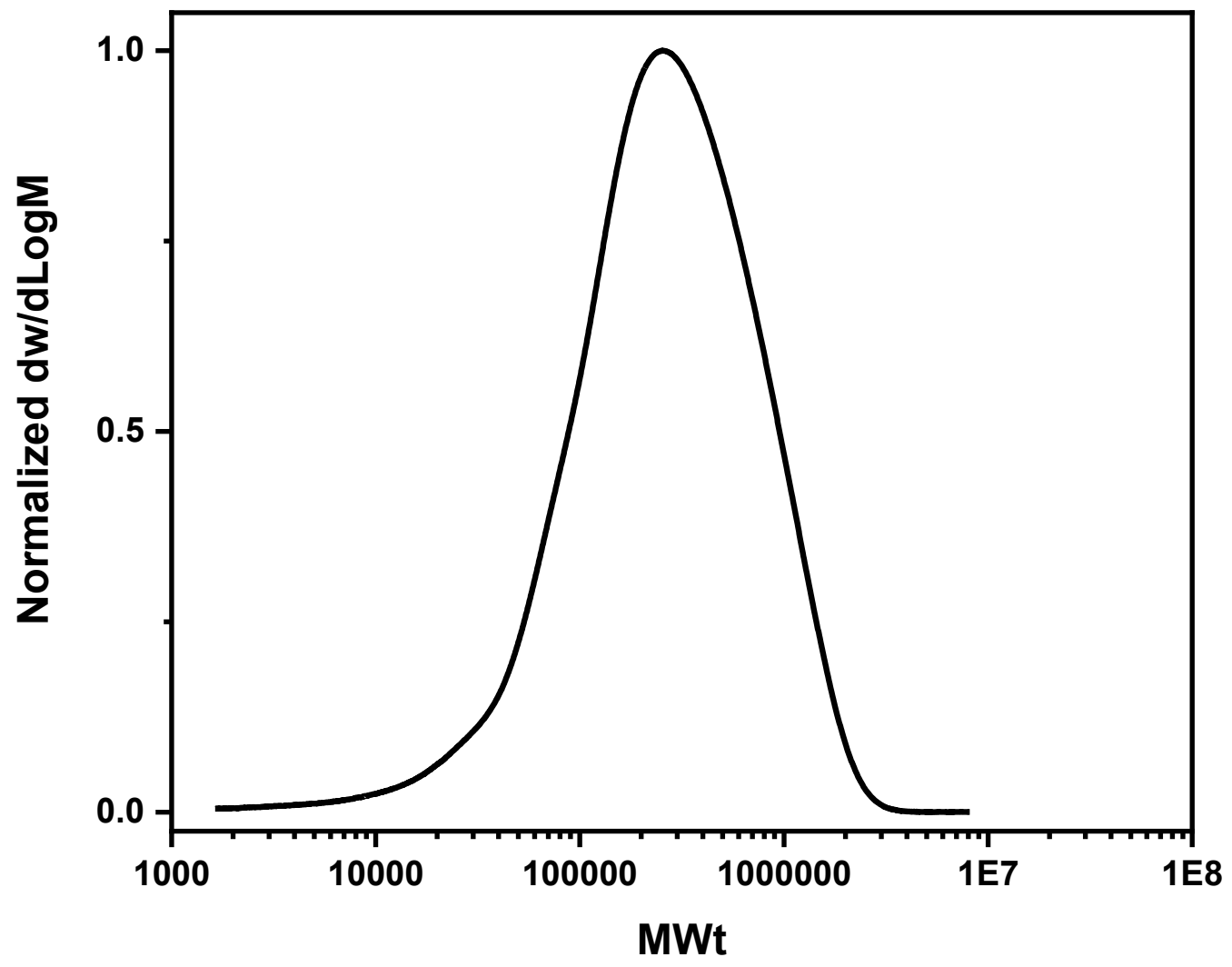

Supplementary Figure 39: SEC trace of PMMA obtained by free radical polymerization. The M_n was 138 kDa and the dispersity was 2.44.

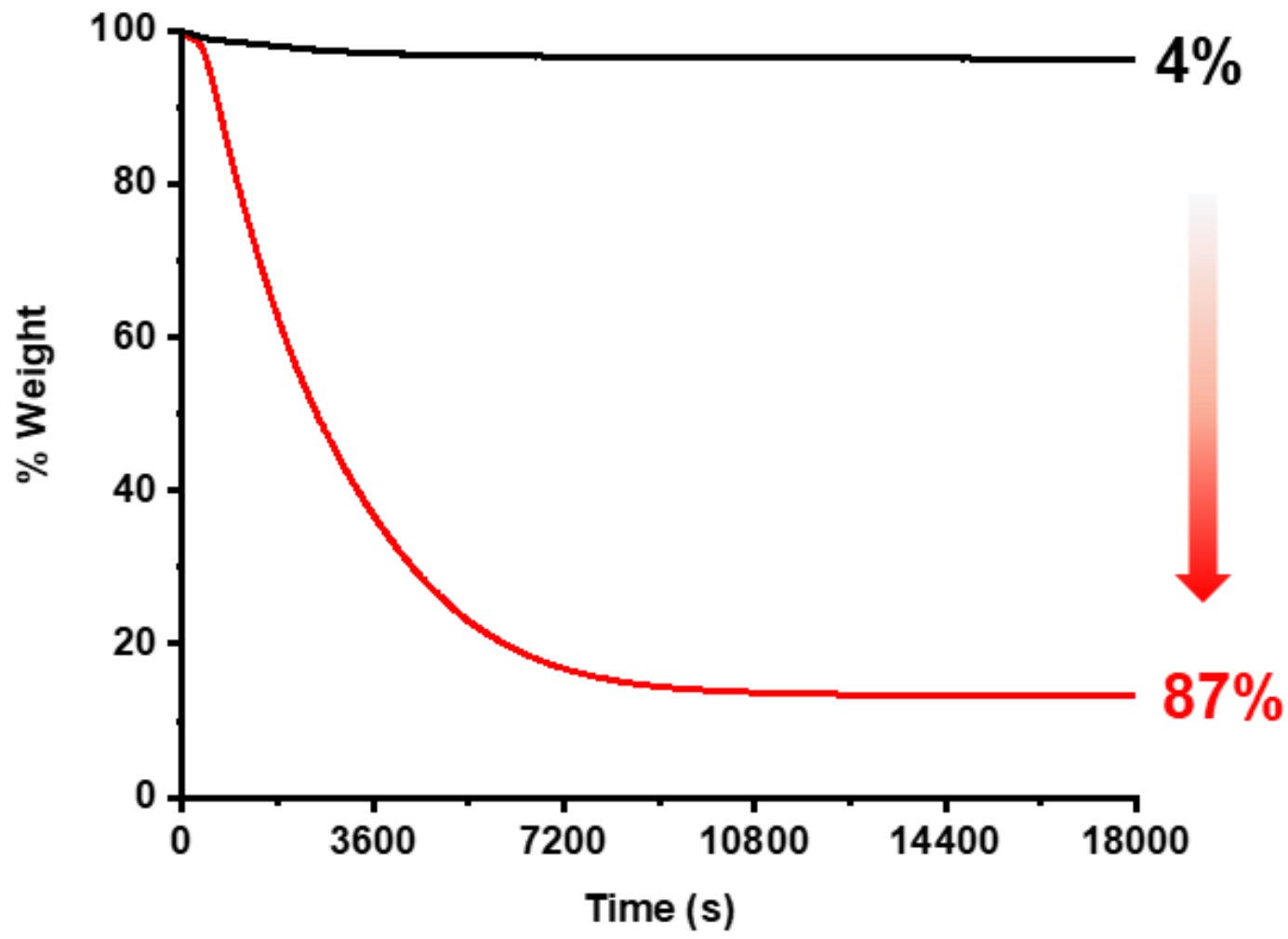

Supplementary Figure 40: TGA data for the depolymerization of PMMA prepared by ATRP. Isothermal reaction was performed for 5 hours at 230 °C with 0.05 equivalents of TCNHPI relative to the polymer repeat unit concentration. The final depolymerization was calculated to be 87%.

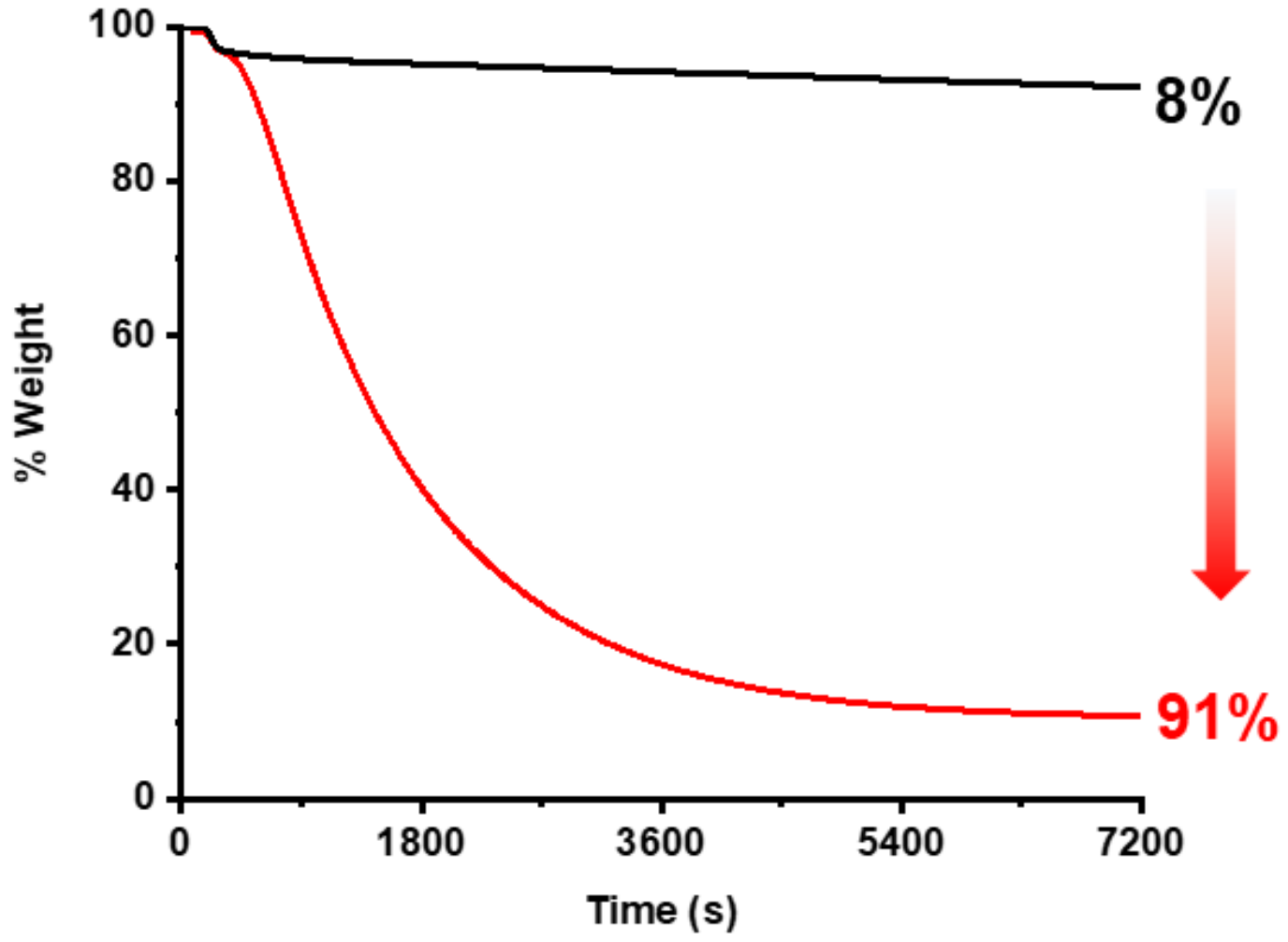

Supplementary Figure 41: TGA data for the depolymerization of a dithiobenzoate terminal PMMA prepared by RAFT. Isothermal reaction was performed for 2 hours at 230 °C with 0.05 equivalents of TCNHPI relative to the polymer repeat unit concentration. The final depolymerization was calculated to be 91%.

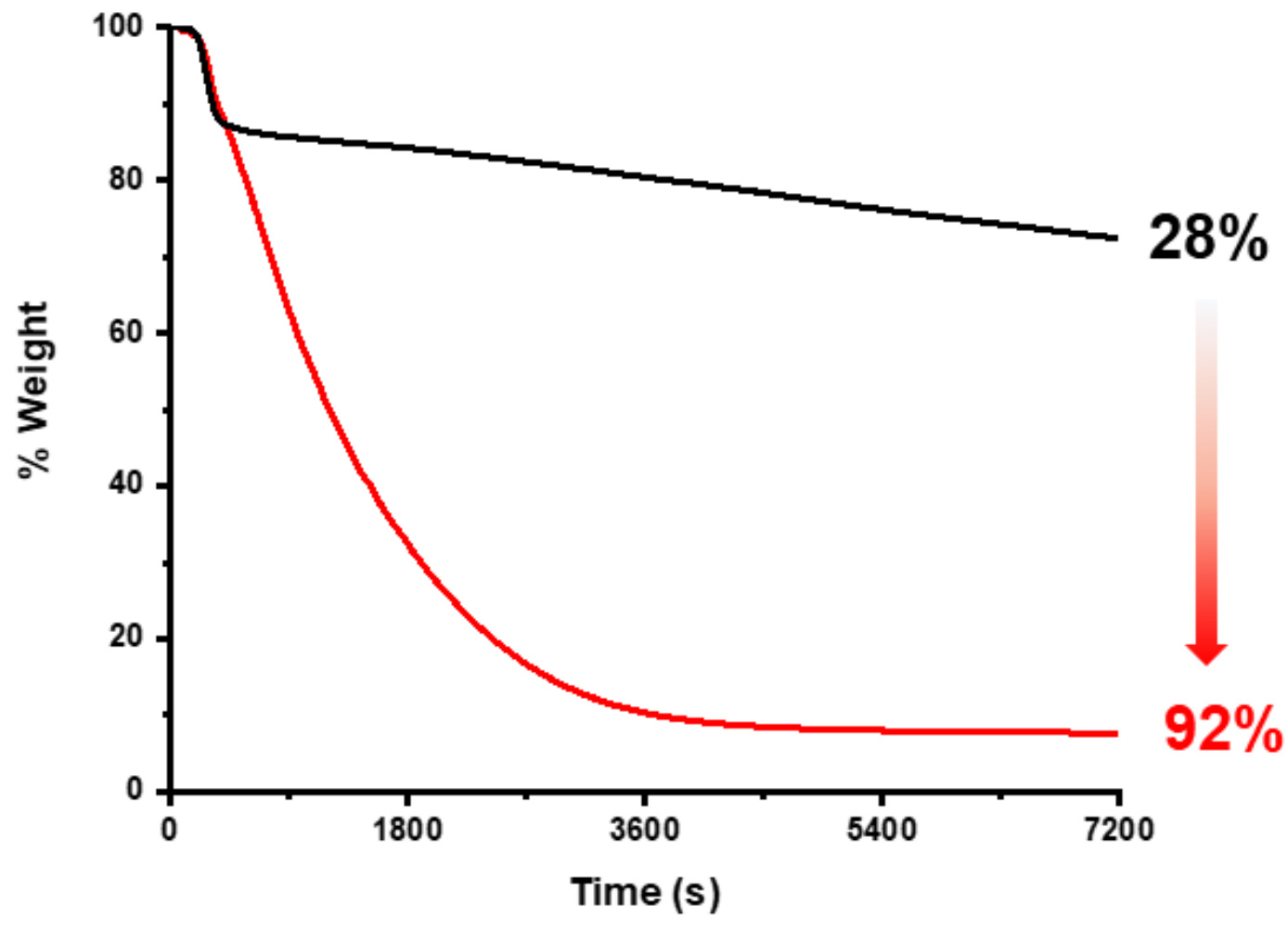

Supplementary Figure 42: TGA data for the depolymerization of a trithiocarbonate terminal PMMA prepared by RAFT. Isothermal reaction was performed for 2 hours at 230 °C with 0.05 equivalents of TCNHPI relative to the polymer repeat unit concentration. The final depolymerization was calculated to be 92%.

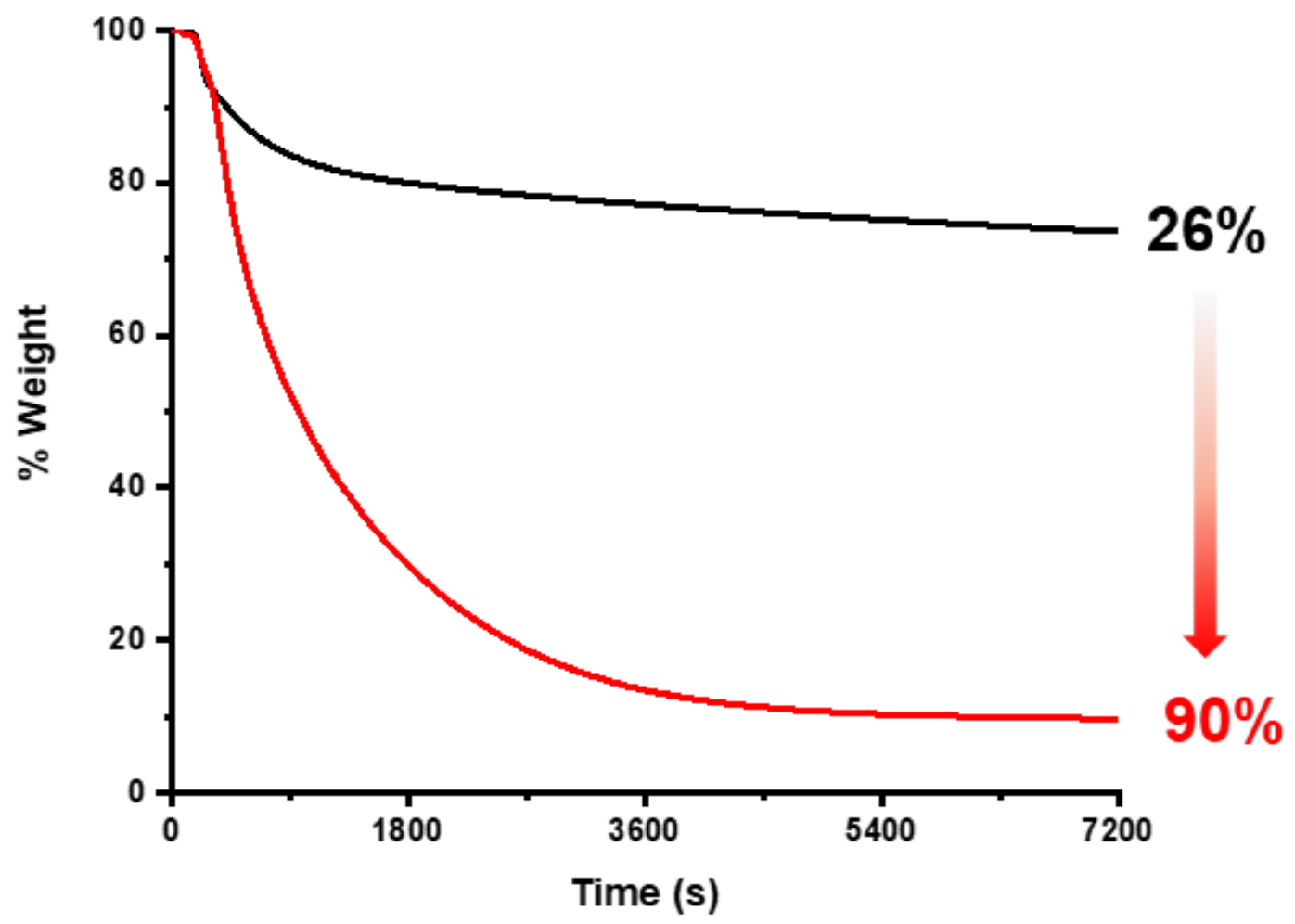

Supplementary Figure 43: TGA data for the depolymerization of a PMMA prepared by free radical polymerization. Isothermal reaction was performed for 2 hours at 230 °C with 0.05 equivalents of TCNHPI relative to the polymer repeat unit concentration. The final depolymerization was calculated to be 90%.

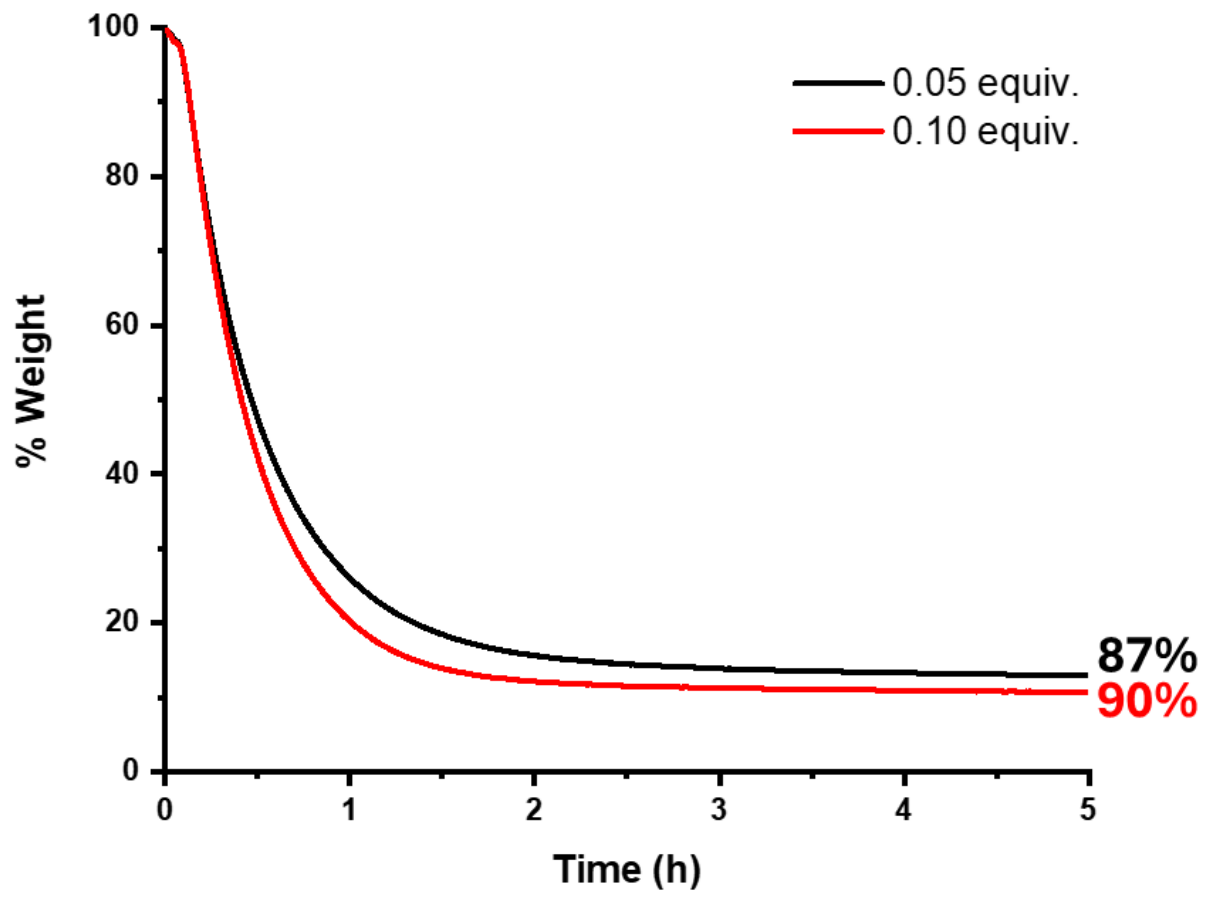

Supplementary Figure 44: TGA data showing the effect of the weight percent of TCNHPI on the depolymerization of PMMA prepared by ATRP. Reaction was performed at 230 °C for 5 hours with either 0.05 or 0.10 equivalents of TCNHPI w.r.t. polymer repeat unit concentration.

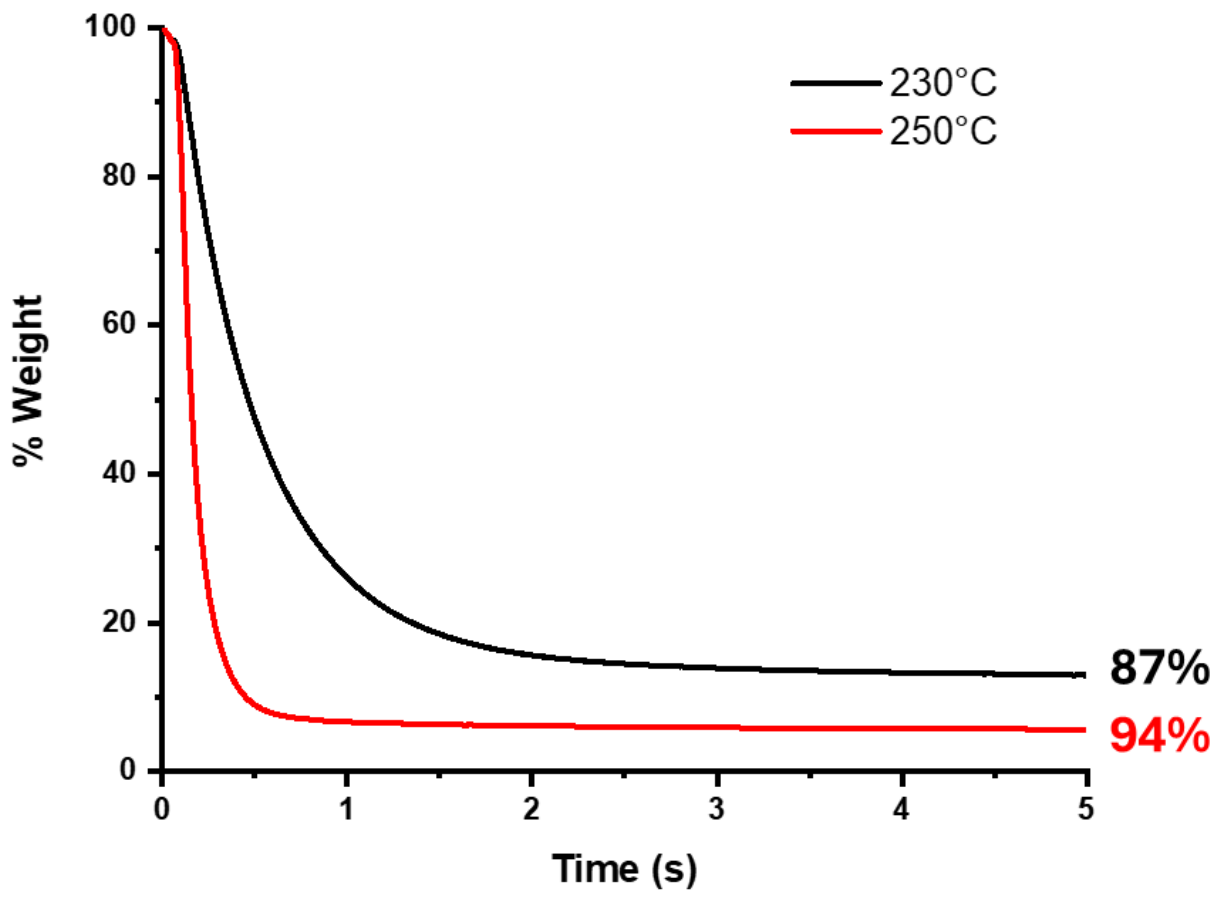

Supplementary Figure 45: TGA data showing the effect of the weight percent of TCNHPI on the depolymerization of PMMA prepared by ATRP. Reaction was performed at either 230 °C or 250°C for 5 hours with 0.05 equivalents of TCNHPI w.r.t. polymer repeat unit concentration.

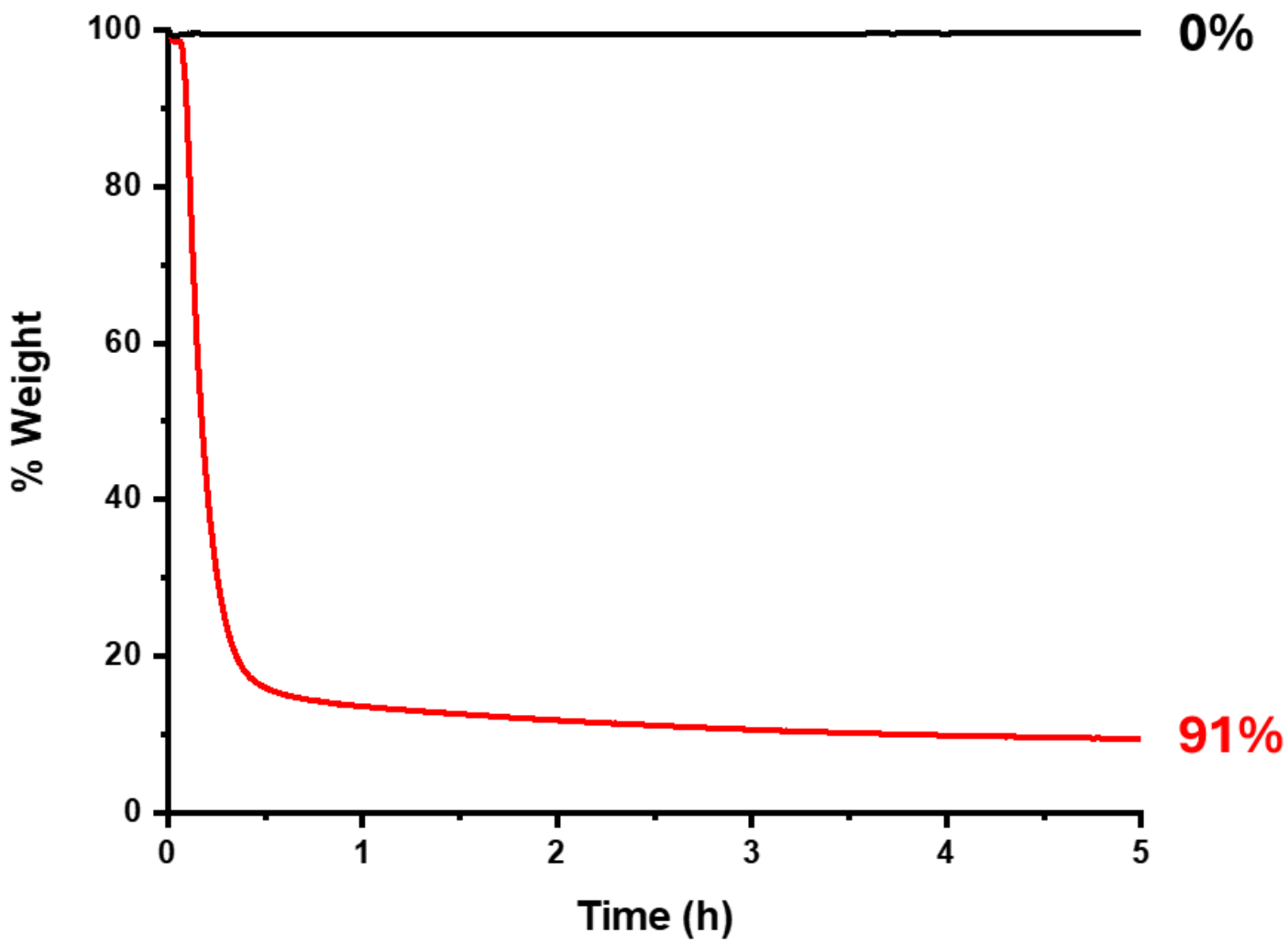

Supplementary Figure 46: TGA data showing the depolymerization of 100kDa PMMA with (red) and without (black) the addition of TCNHPI. The reaction was performed at 230°C for 5 hours with 0.05 equivalents of TCNHPI w.r.t. polymer repeat unit concentration.

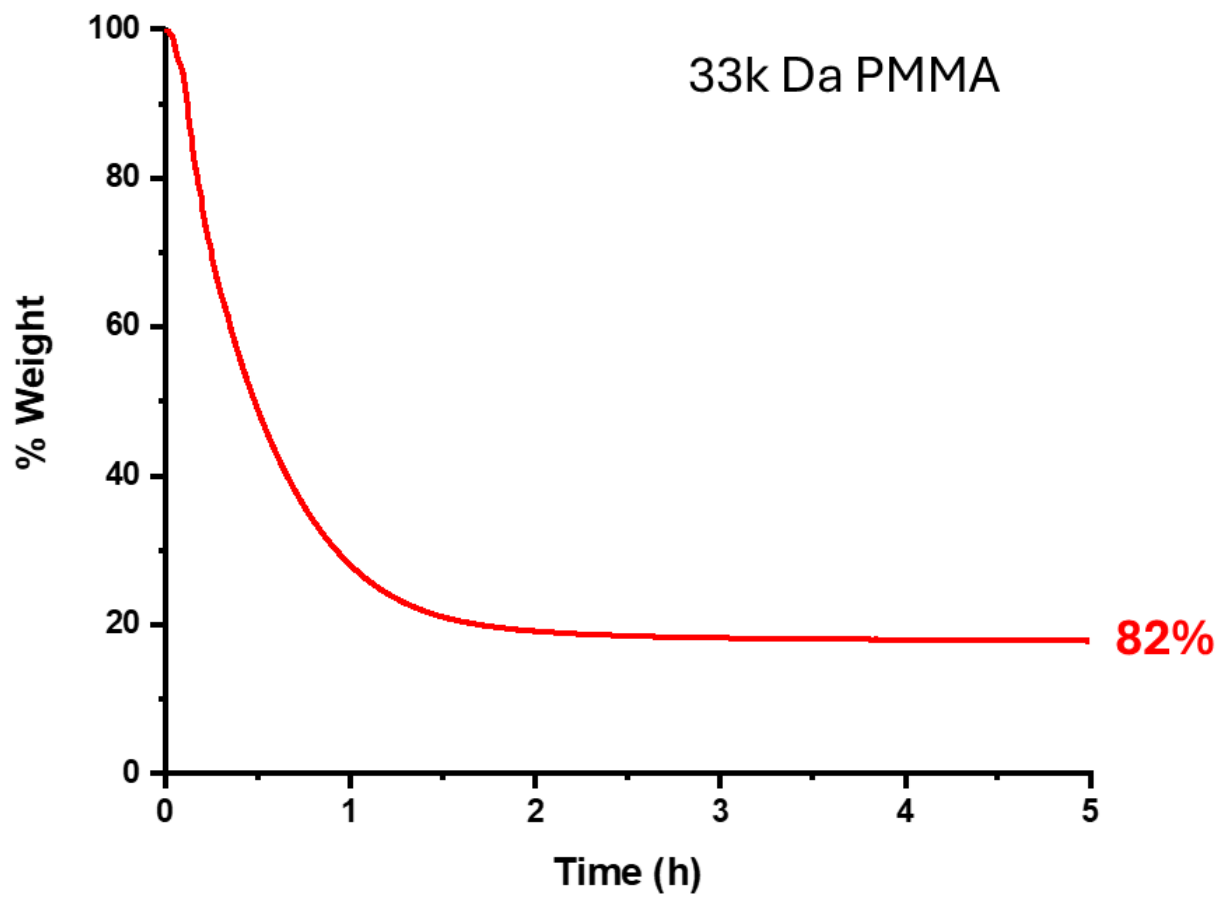

Supplementary Figure 47: TGA data showing the depolymerization of 33kDa PMMA. The reaction was performed at 230 °C for 5 hours with 0.05 equivalents of TCNHPI w.r.t. polymer repeat unit concentration.

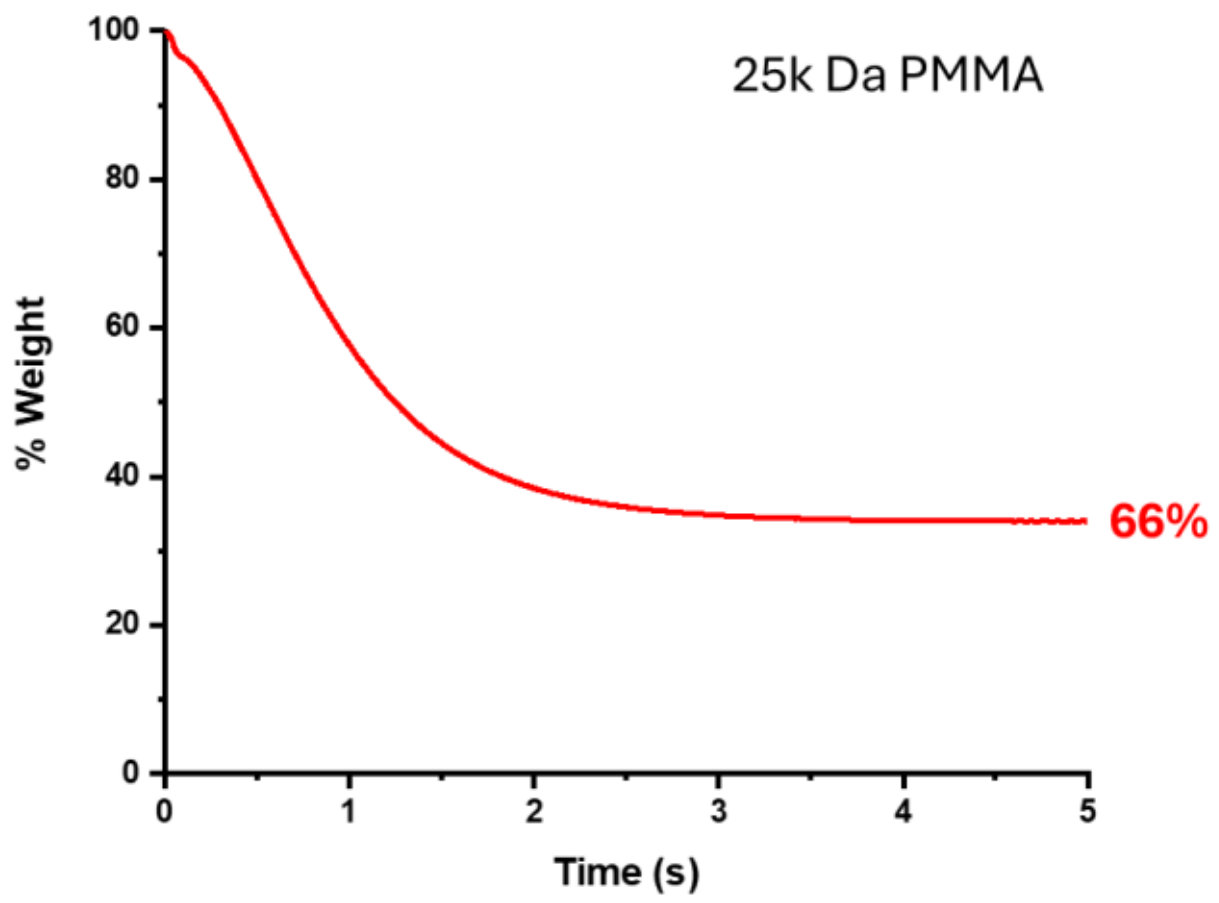

Supplementary Figure 48: TGA data showing the depolymerization of 25kDa PMMA. Reaction was performed at 230 °C for 5 hours with 0.05 equivalents of TCNHPI w.r.t. polymer repeat unit concentration.

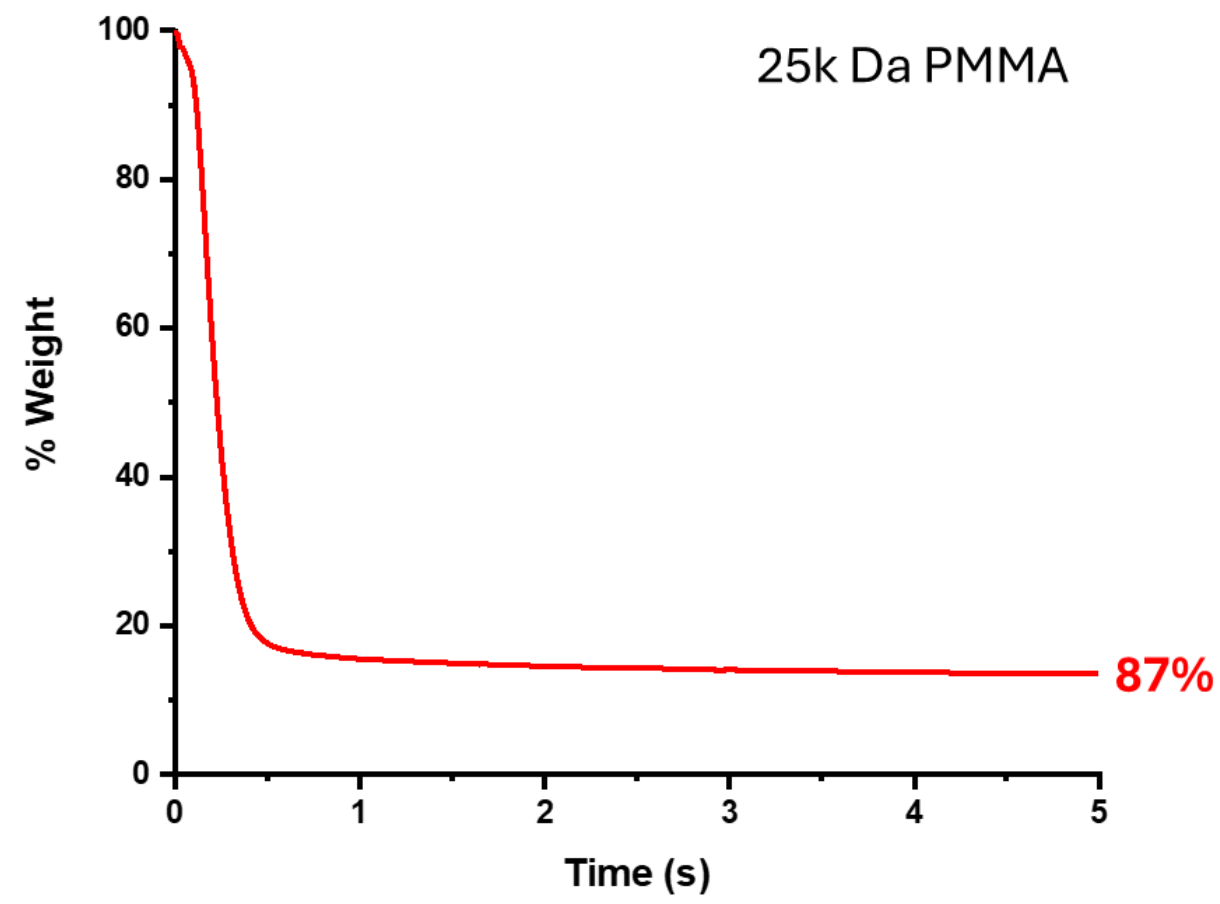

Supplementary Figure 49: TGA data showing the depolymerization of 25kDa PMMA. Reaction was performed at 250°C for 5 hours with 0.10 equivalents of TCNHPI w.r.t. polymer repeat unit concentration.

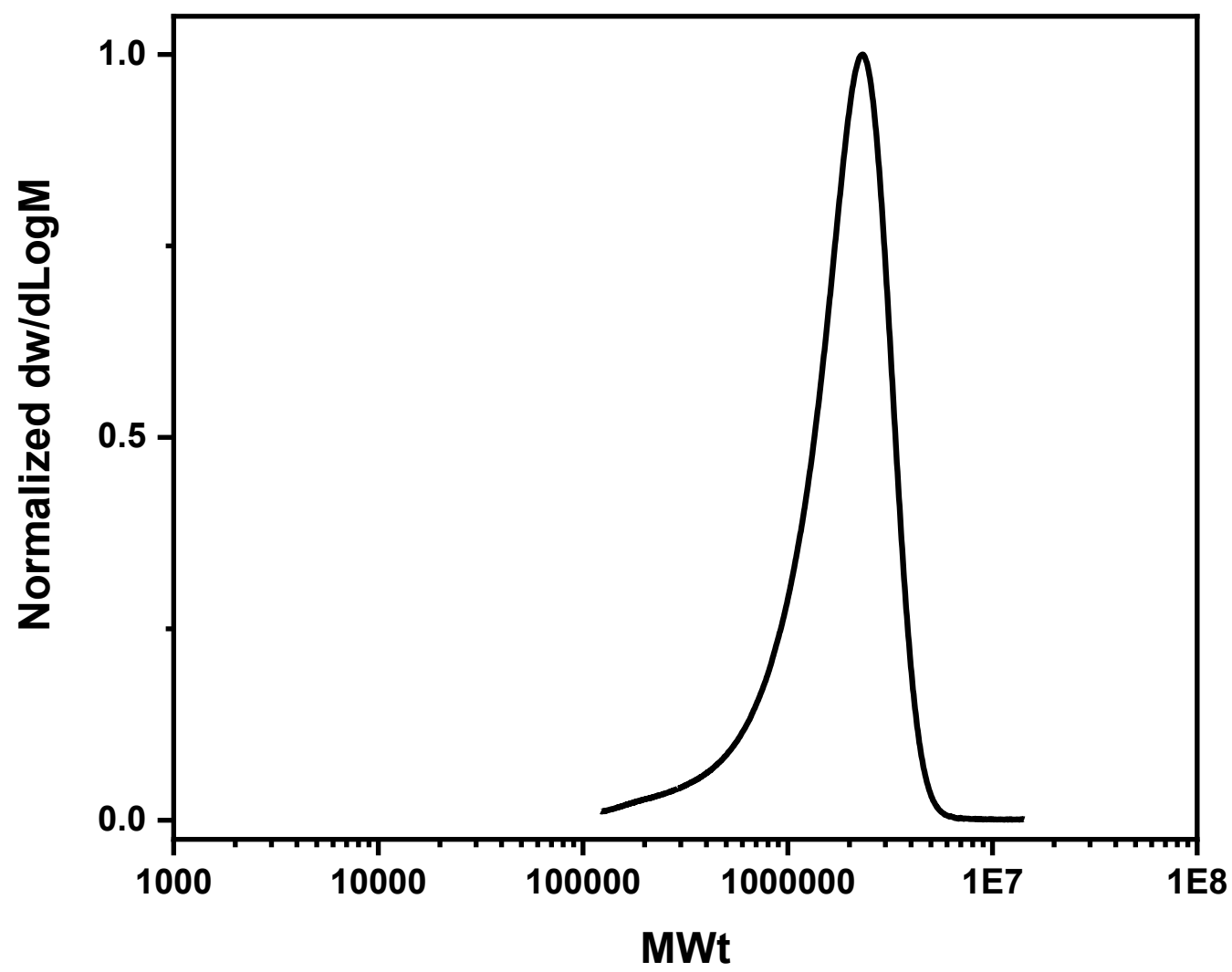

Supplementary Figure 50: SEC trace of high molecular weight PMMA prepared by anionic polymerization. The M_n was 1.4 MDa.

Supplementary Table 12: Table of data obtained from kinetic experiments of 1.4MDa PMMA, illustrating the effect of depolymerization on the molecular weight distribution of the remaining polymer chains. Isothermal TGA reactions were performed at 230 °C for various times using 0.05 equivalents of TCNHPI w.r.t polymer repeat unit concentration and a polymer made by anionic polymerization.

Time of Experiment (seconds)	Depolymerization %	M_n	M_w	*Đ*
0	0	1400000		
396	15	190000	472000	2.49
520	30	112000	291000	2.59
670	45	85000	177000	2.07
906	60	50000	93400	1.89
1303	75	34000	59000	1.72
2441	90	22000	37000	1.70

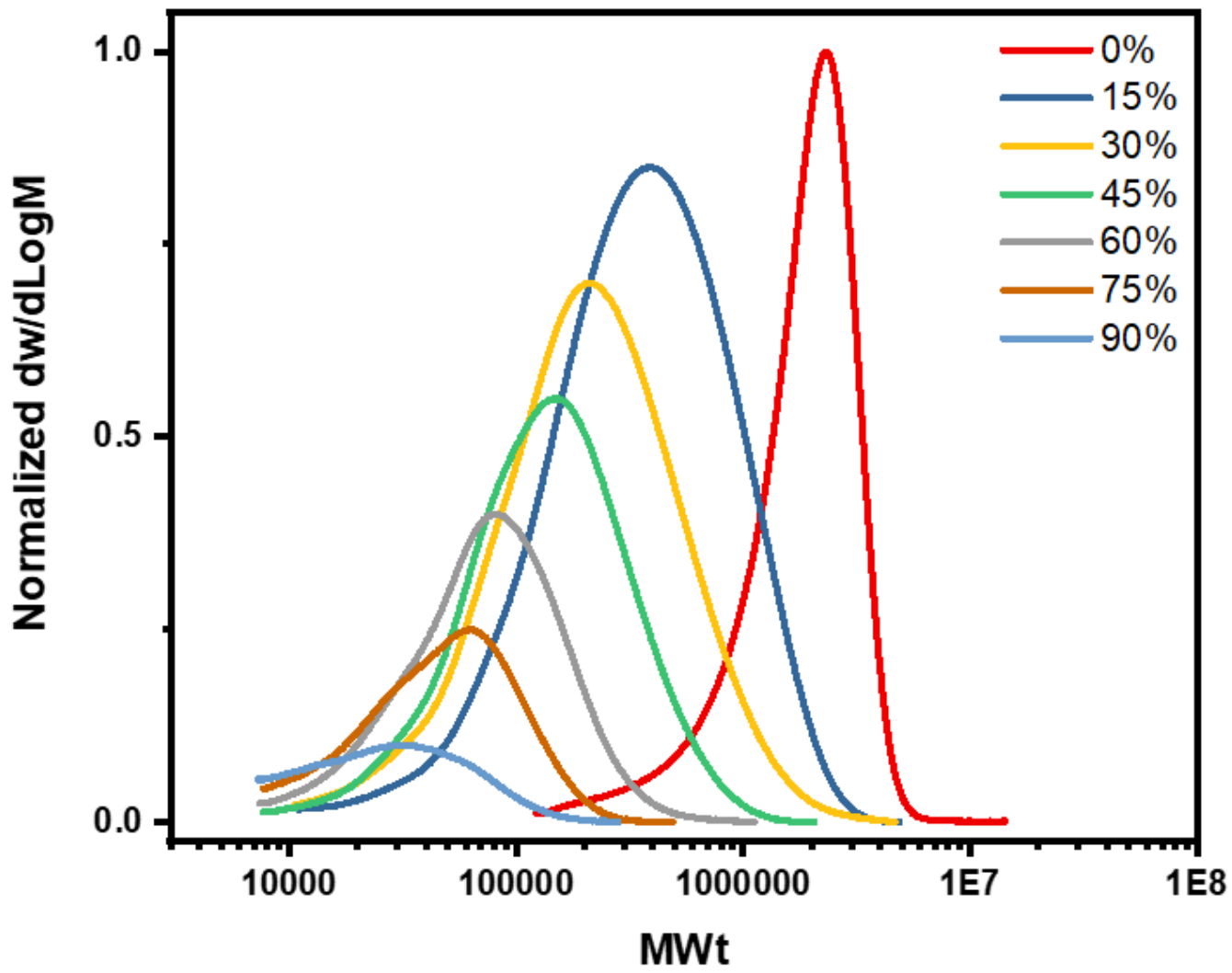

Supplementary Figure 51: SEC traces presented at various extents of depolymerization of the 1.4MDa PMMA. Reaction was performed at 230 °C for various times using 0.05 equivalents of TCNHPI w.r.t polymer repeat unit concentration.

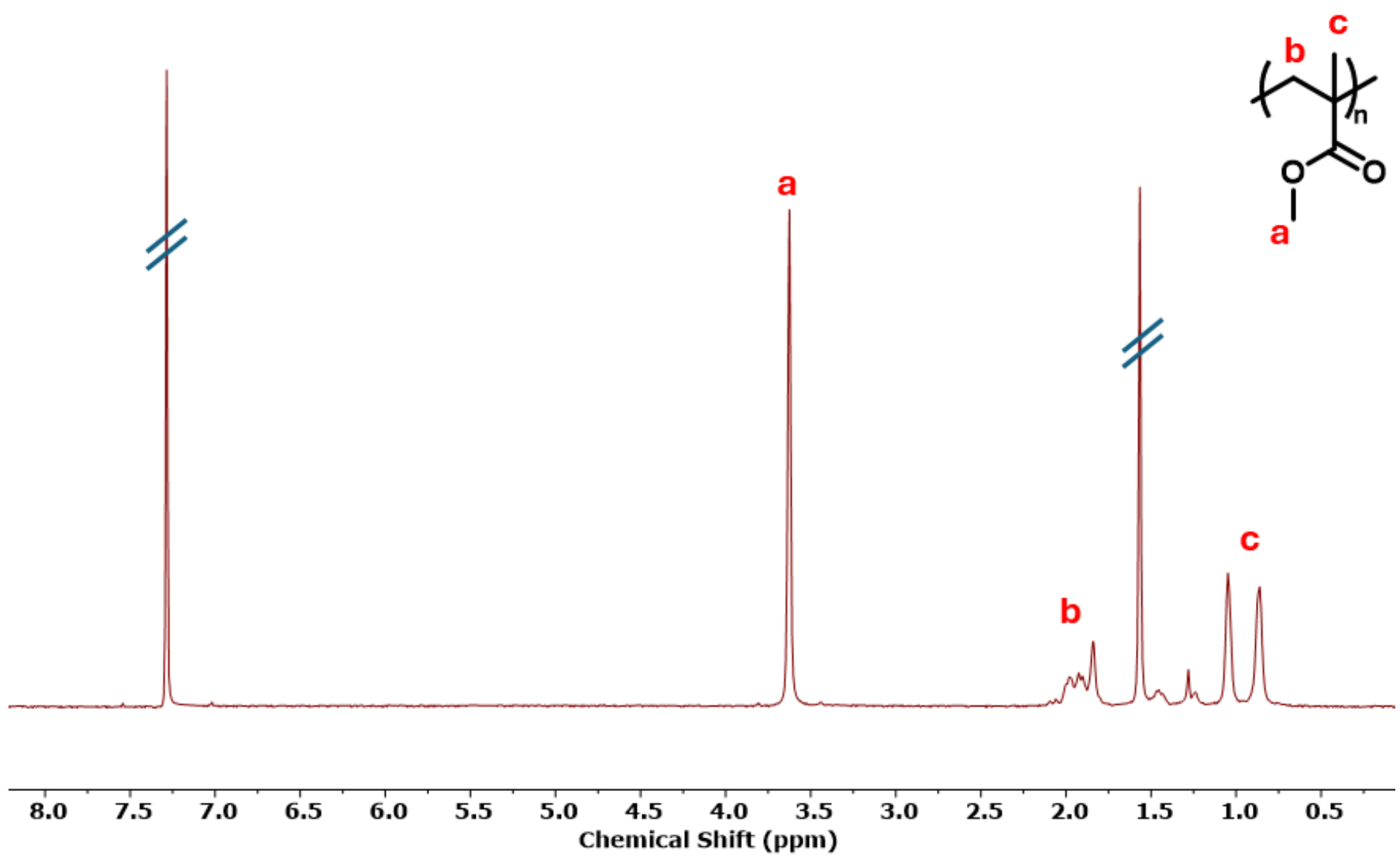

Supplementary Figure 52: ^{1}H NMR of the remaining polymer obtained after 96% of depolymerization of the 1.4MDa PMMA.

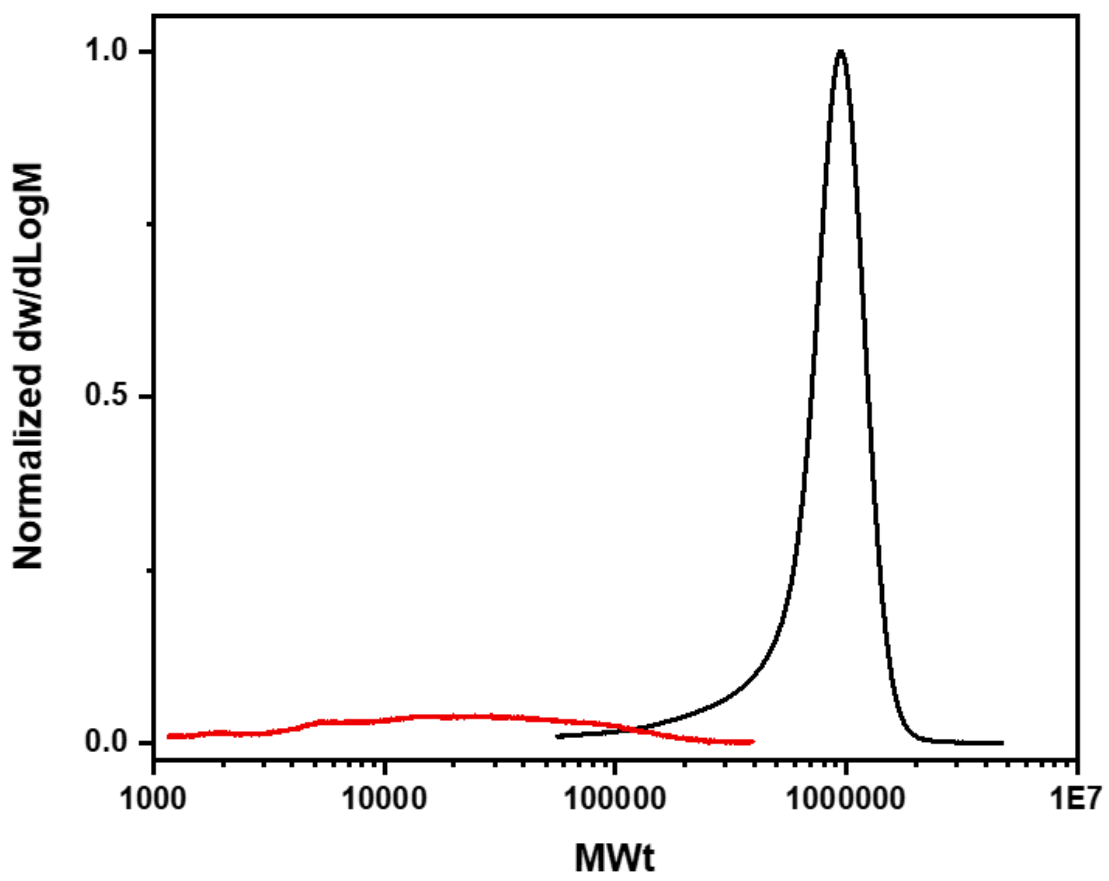

Supplementary Figure 53: SEC traces presented before and after the depolymerization of the 1.4MDa PMMA.

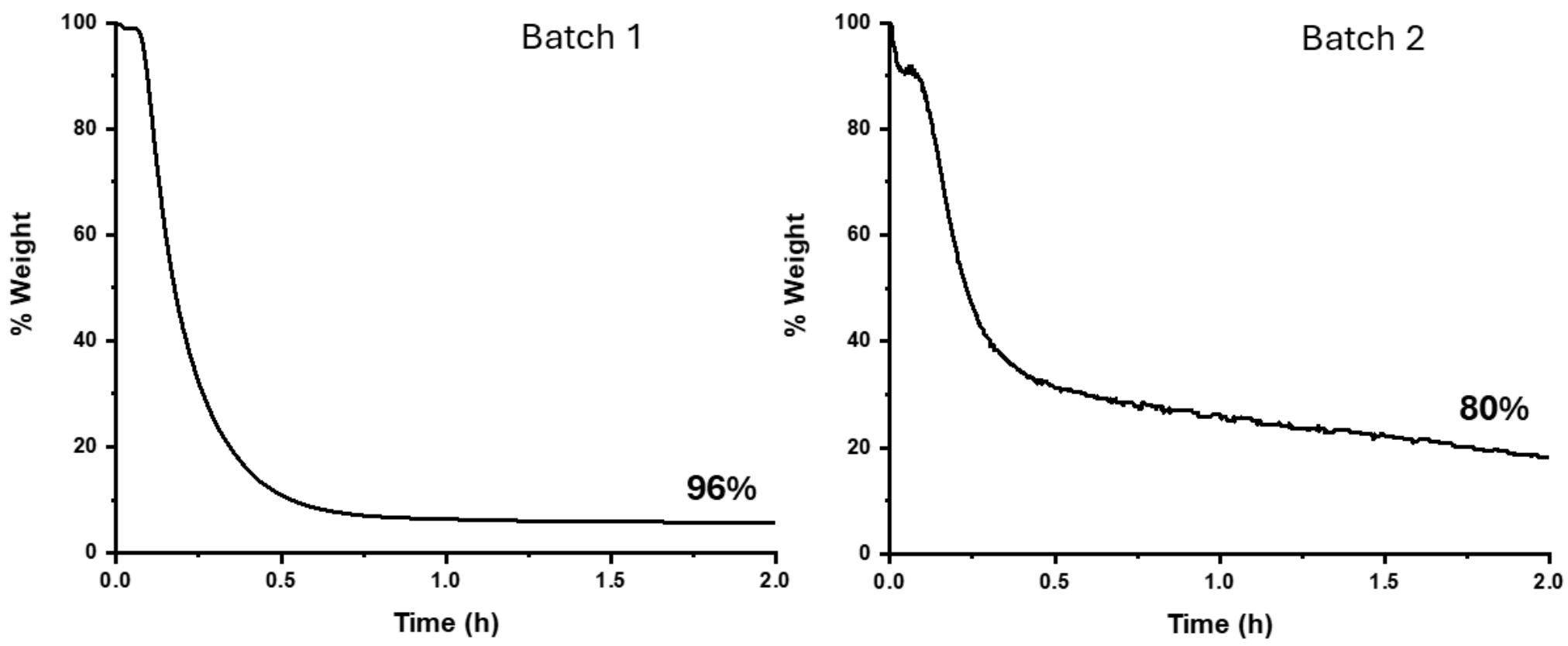

Supplementary Figure 54: TGA data showing the effect of adding a second batch of TCNHPI after depolymerization plateau. It was possible to depolymerize the unreacted 4% of material to 80%, giving an overall yield of >99%. Reactions were performed for 2 hours with 0.05 equivalents of TCNHPI w.r.t. polymer repeat unit concentration.

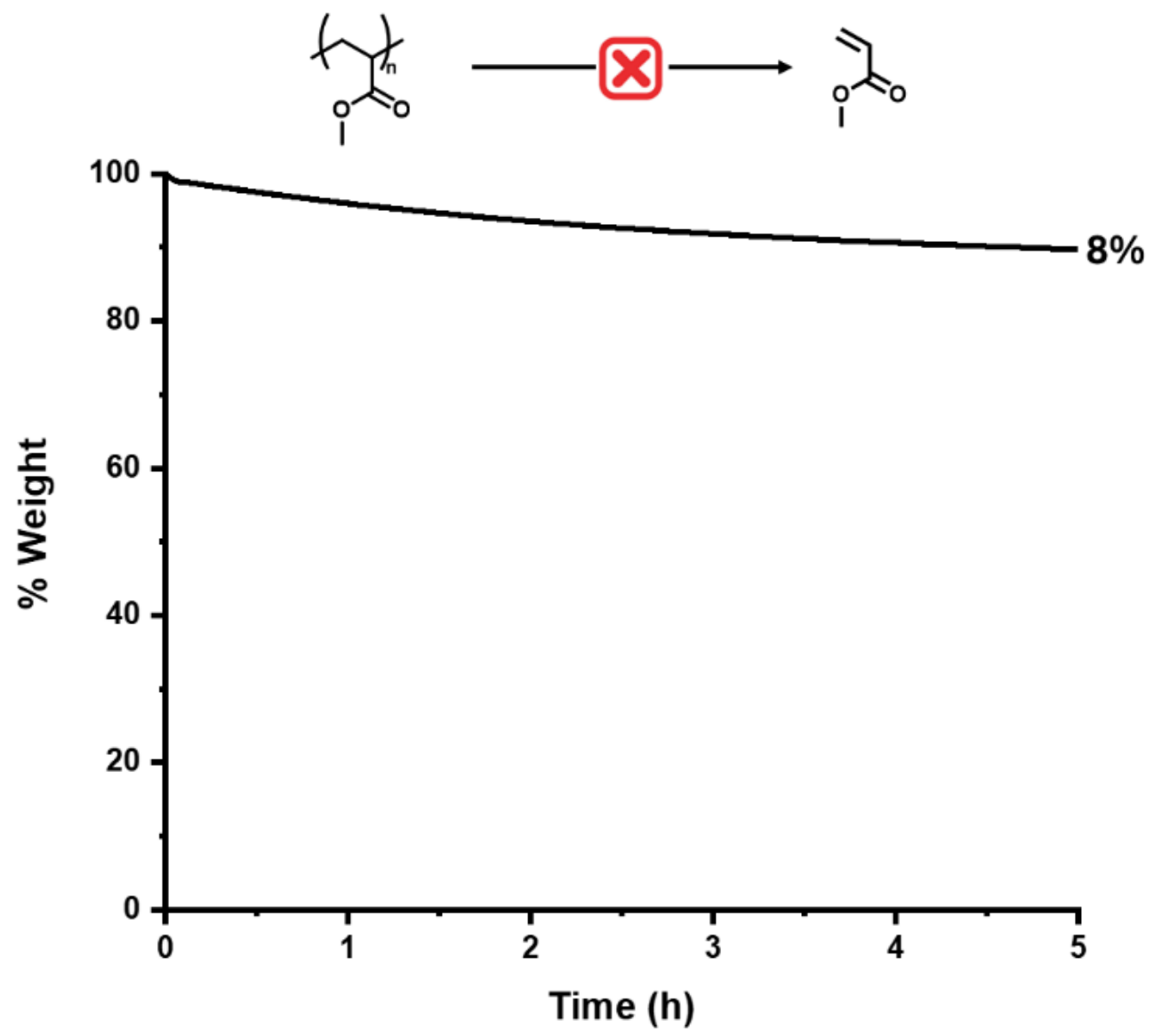

Supplementary Figure 55: TGA data showing the reaction of PMA with TCNHPI. Reaction was performed at 230 °C for 5 hours with 0.05 equivalents of TCNHPI w.r.t. polymer repeat unit concentration.

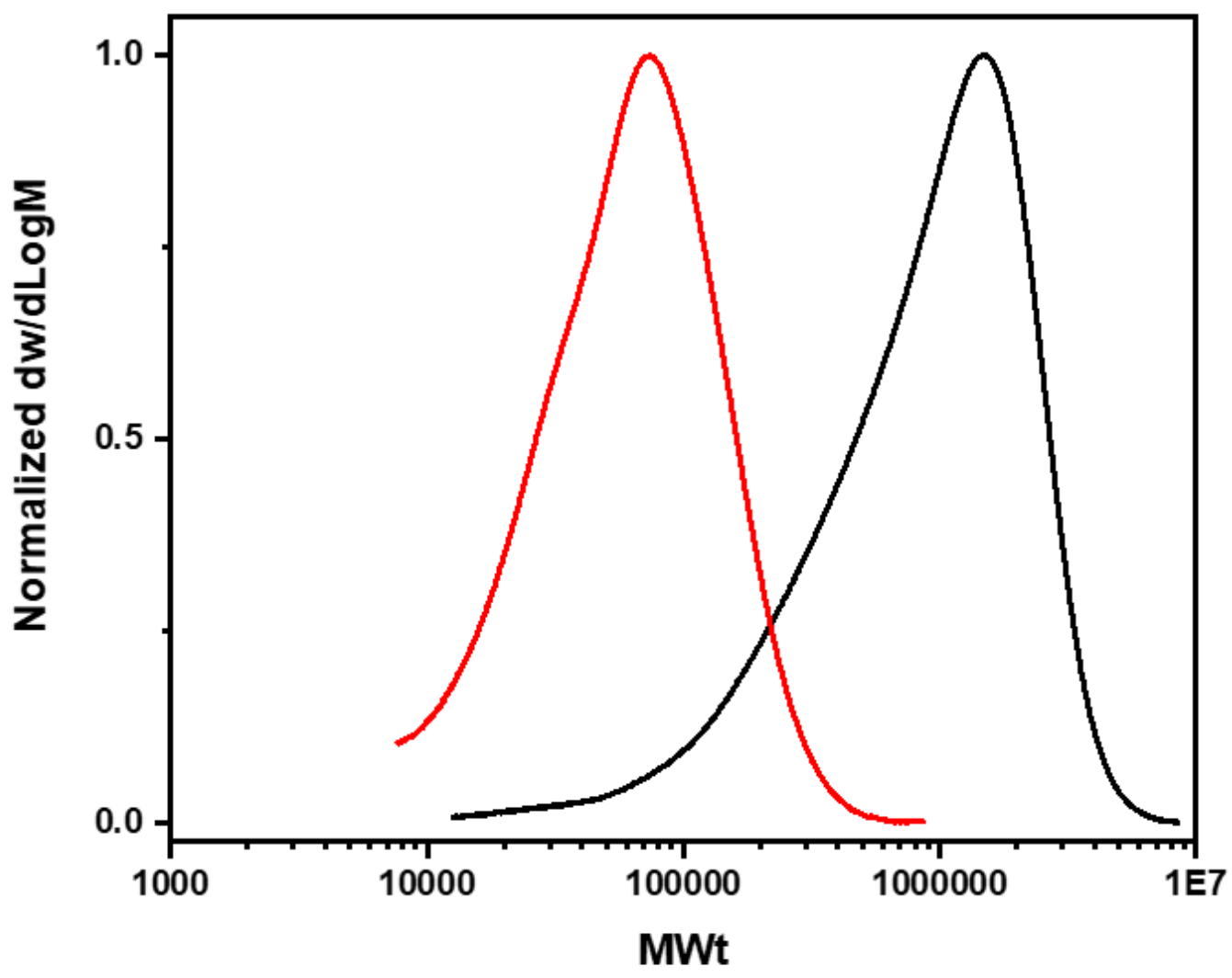

Supplementary Figure 56: SEC data showing the evolution of the PMA molecular weight distribution on heating to 230 °C with TCNHPI for 5 hours. The M_n dropped from 400kDa (black) to 43kDa (red), suggesting significant scission had occurred during the process.

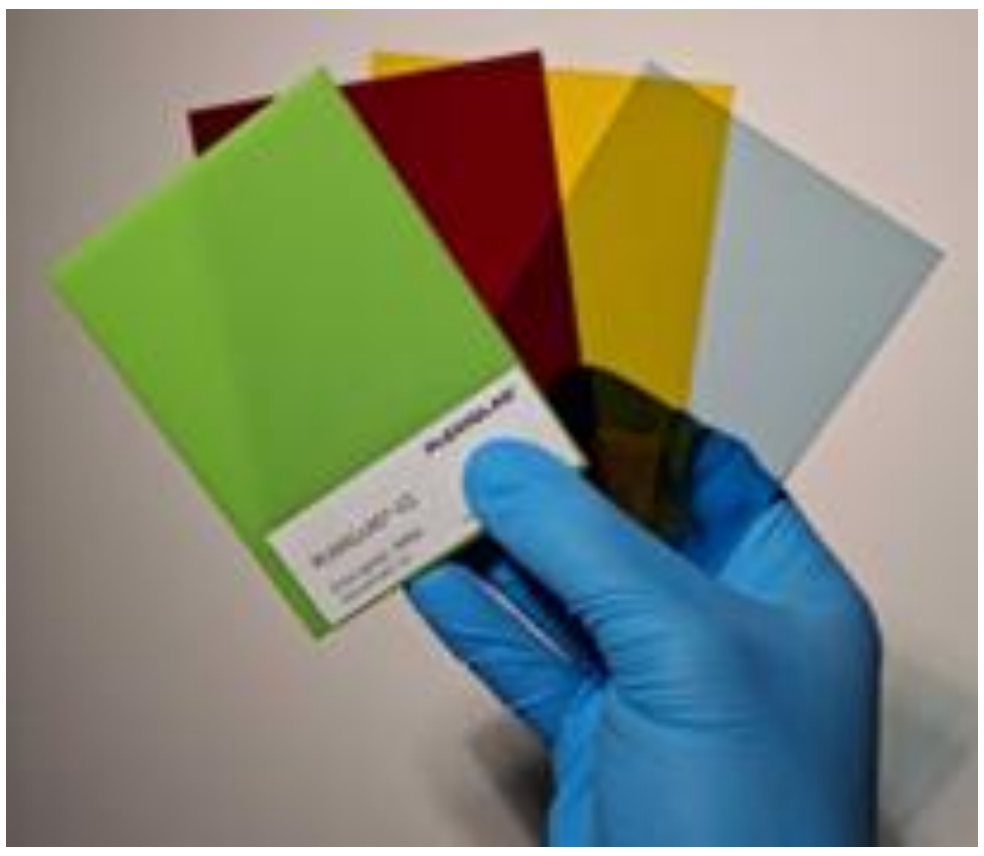

Supplementary Figure 57: Photographs of commercial Plexiglas. From left to right, green, red, yellow and blue sheets are shown.

Supplementary Table 13: The depolymerization of various colors of Plexiglas. Reactions were performed with 0.05 equivalents of TCNHPI with respect to polymer repeat unit at 230 °C for 2 hours.

Colour of Plexiglas	TCNHPI	Depolymerization %
Blue	No	13
	Yes	91
Yellow	Yes	85
Red	Yes	86
Green	Yes	91
Mixed	Yes	90

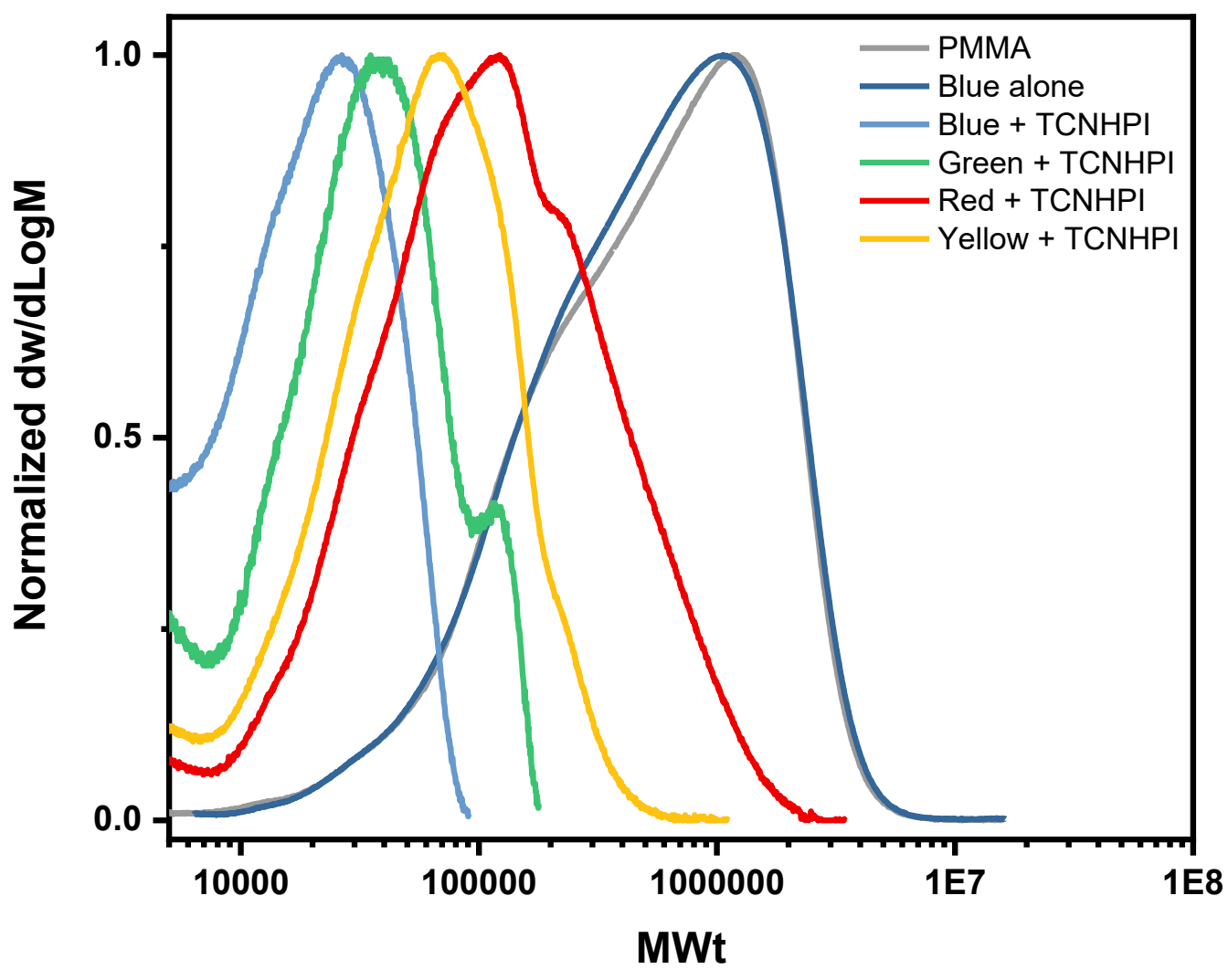

Supplementary Figure 58: SEC data showing the effect of depolymerization on the molecular weight distribution of various colors of Plexiglas. Reactions were performed with 0.05 equivalents of TCNHPI with respect to polymer repeat unit at 230 °C for 2 hours. The starting M_n of the Plexiglas was 173 kDa and all SECs are normalized by maximum intensity.

Supplementary Table 14: The high-scale depolymerization of PMMA performed in a distillation apparatus. The table summarizes the extent of depolymerization and the data corresponds to Figure 5d.

Time (h)	Mass of Polymer (g)	Mass of Monomer collected (g)	Depolymerization %
0	5.1	0	0
4	0.5	4.6	92

References

1 Zichittella, G., Polyhach, Y., Tschaggelar, R., Jeschke, G. & Pérez-Ramírez, J. Quantification of redox sites during catalytic propane oxychlorination by operando EPR spectroscopy. *Angewandte Chemie* **133**, 3640-3646 (2021).

2 Stoll, S. & Schweiger, A. EasySpin, a comprehensive software package for spectral simulation and analysis in EPR. *Journal of magnetic resonance* **178**, 42-55 (2006).

3 Frisch, M. gaussian 09, Revision d. 01, Gaussian. *Inc, Wallingford CT* **201** (2009).

4 Zhao, Y. & Truhlar, D. G. The M06 suite of density functionals for main group thermochemistry, thermochemical kinetics, noncovalent interactions, excited states, and transition elements: two new functionals and systematic testing of four M06-class functionals and 12 other functionals. *Theoretical chemistry accounts* **120**, 215-241 (2008).

5 Hohenstein, E. G., Chill, S. T. & Sherrill, C. D. Assessment of the performance of the M05− 2X and M06− 2X exchange-correlation functionals for noncovalent interactions in biomolecules. *Journal of Chemical Theory and Computation* **4**, 1996-2000 (2008).

6 Krishnan, R., Binkley, J. S., Seeger, R. & Pople, J. A. Self-consistent molecular orbital methods. XX. A basis set for correlated wave functions. *J. Chem. Phys.* **72**, 650-654 (1980).

7 Atkins, P. W., De Paula, J. & Keeler, J. *Atkins' physical chemistry*. (Oxford university press, 2023).

8 Ghosh, R. & Olofsson, B. Metal-free synthesis of N-aryloxyimides and aryloxyamines. *Org. Lett.* **16**, 1830-1832 (2014).

9 Pronkin, D. K. *et al.* Thermal Stability of Several Organic Hydroxylamine Derivatives and a Broader Look at Explosive Hazard Identification. *Ind. Eng. Chem. Res.* **64**, 1139–1151 (2025).
